# De novo genes with an lncRNA origin encode unique human brain developmental functionality

**DOI:** 10.1038/s41559-022-01925-6

**Published:** 2023-01-02

**Authors:** Ni A. An, Jie Zhang, Fan Mo, Xuke Luan, Lu Tian, Qing Sunny Shen, Xiangshang Li, Chunqiong Li, Fanqi Zhou, Boya Zhang, Mingjun Ji, Jianhuan Qi, Wei-Zhen Zhou, Wanqiu Ding, Jia-Yu Chen, Jia Yu, Li Zhang, Shaokun Shu, Baoyang Hu, Chuan-Yun Li

**Affiliations:** 1 Laboratory of Bioinformatics and Genomic Medicine, Institute of Molecular Medicine, Peking University, Beijing, China; 2 State Key Laboratory of Stem Cell and Reproductive Biology, Institute of Stem Cell and Regeneration, Institute of Zoology, Chinese Academy of Sciences, Beijing, China; 3 University of Chinese Academy of Sciences, Beijing, China; 4 Chinese Institute for Brain Research, Beijing, China; 5 State Key Laboratory of Medical Molecular Biology, Key Laboratory of RNA Regulation and Hematopoiesis, Department of Biochemistry and Molecular Biology, Institute of Basic Medical Sciences, School of Basic Medicine, CAMS and Peking Union Medical College, Beijing, China; 6 State Key Laboratory of Cardiovascular Disease, Fuwai Hospital, Chinese Academy of Medical Sciences and Peking Union Medical College, Beijing, China; 7 State Key Laboratory of Pharmaceutical Biotechnology, School of Life Sciences, Chemistry and Biomedicine Innovation Center (ChemBIC), Nanjing University, Nanjing, China; 8 Peking University International Cancer Institute, Beijing, China

**Keywords:** Molecular evolution, Evolutionary developmental biology

## Abstract

Human de novo genes can originate from neutral long non-coding RNA (lncRNA) loci and are evolutionarily significant in general, yet how and why this all-or-nothing transition to functionality happens remains unclear. Here, in 74 human/hominoid-specific de novo genes, we identified distinctive U1 elements and RNA splice-related sequences accounting for RNA nuclear export, differentiating mRNAs from lncRNAs, and driving the origin of de novo genes from lncRNA loci. The polymorphic sites facilitating the lncRNA–mRNA conversion through regulating nuclear export are selectively constrained, maintaining a boundary that differentiates mRNAs from lncRNAs. The functional new genes actively passing through it thus showed a mode of pre-adaptive origin, in that they acquire functions along with the achievement of their coding potential. As a proof of concept, we verified the regulations of splicing and U1 recognition on the nuclear export efficiency of one of these genes, the *ENSG00000205704*, in human neural progenitor cells. Notably, knock-out or over-expression of this gene in human embryonic stem cells accelerates or delays the neuronal maturation of cortical organoids, respectively. The transgenic mice with ectopically expressed *ENSG00000205704* showed enlarged brains with cortical expansion. We thus demonstrate the key roles of nuclear export in de novo gene origin. These newly originated genes should reflect the novel uniqueness of human brain development.

## Abstract (web summary)

Studying human-specific de novo genes originated from long non-coding RNA, the authors reveal molecular mechanisms that facilitate nuclear export of these young genes, and show experimental evidence for the role of one such gene in brain development.

## Main

Although gene duplication has been reported as the predominant mechanism of the origin of new genes^1–3^, recent studies have proposed that new proteins can also evolve de novo from non-coding DNA regions^4–12^. More specifically, we and others have found that de novo genes show expression and splicing profiles similar to their orthologues encoding long non-coding RNA (lncRNAs) in out-group species, indicating a ‘transcription-first’ model in which protein-coding genes may originate from ancestral lncRNA loci^4,7,13,14^. As lncRNAs enrich in the nucleus fraction relative to messenger RNAs in eukaryotes^15^, it is interesting to investigate whether a transition of subcellular localization occurs along with the origin of de novo genes from ancestral lncRNA loci. Moreover, if such a transition occurs, what molecular mechanism drives it? Notably, recent studies have implicated both *cis* elements^16–24^ and *trans* factors^25^ into the regulations of RNA nuclear export, providing clues to the molecular basis underlying such a transition. However, to fully clarify the origin of de novo genes from lncRNA loci, a genome-wide, hypothesis-free approach is still needed to systematically identify the predominant factors differentiating mRNAs from lncRNAs in terms of nuclear export activity.

It is also difficult to understand the process by which a de novo gene acquires its biological function. Two hypotheses have been proposed for this evolutionary transition: the continuum model, which claims a slow, step-like process^8^, and the pre-adaptation model, which proposes the existence of exaggerated gene-like characteristics in new genes and an all-or-nothing transition to functionality^26^. In the latter scenario, the precursors of the new genes were proposed to represent the ‘hopeful monsters’ after the imperative avoidance of the most toxic ‘hopeless monster’ in a pre-adaptive process^27^. Although the true process of function acquisition remains to be fully clarified, the findings of several pilot studies focusing on different features in de novo gene origin seem to support the involvement of both models^8,13,26,28–30^. Notably, we and others found that de novo genes in humans, fruit flies and yeast are selectively constrained in general^13,14,29^, supporting a pre-adaptation process that the de novo genes become functional along with their origination. However, it is still difficult to understand the molecular mechanisms underpinning these models, such as the boundaries for natural selection occurs to remove the toxic ‘hopeless monsters’ in the pre-adaptation model, and the selection and molecular basis underlying the optimization process in the continuum model.

Although these de novo genes in human are selectively constrained in general, their biological functions remain to be addressed. Recently, pilot studies have linked new genes arising through gene duplication^31–38^, new microRNAs^39–41^ and new regulatory mechanisms of old genes to distinctive human features of brain development^42^. Regulations before and after the onset of neurogenesis have also been proposed to explain the enlarged brain during primate evolution, from the perspectives of the expansion of the neocortical primordium through increased neuroepithelial cells^42–44^ and the thickening of the cortical layers through the expansion of radial glial cells (RGCs)^45–49^. Considering that de novo genes show brain-enriched expression profiles, particularly in human foetal brains^50^, it is plausible that these genes also play adaptive roles in brain development.

However, it is not straightforward to pinpoint the causal relationships between these genes and human-specific traits, as studies in cell culture can provide only limited insights into the higher-order functions at the cell type, organ or even whole animal level. Notably, recent advances in human cortical organoids provide a practical model to mimic human early brain development^51^. Using this system, pilot comparative studies in human and chimpanzee organoids have observed a lengthening of prometaphase–metaphase in human neural progenitors, a lower proportion of human neurogenic basal progenitors and a delayed maturation of the human brain^51–53^. However, these human-specific features identified by comparative genomics have remained correlative without being clarified through the manipulation of human-specific genetic elements. In this article, we identified 74 human/hominoid-specific de novo genes and addressed these key issues using human-macaque comparative genomics and experimental verification in cell lines and human cortical organoids. We clarified the process of de novo gene origination from lncRNAs, proposed RNA nuclear export as the selection boundary shaping the preadaptation pattern in de novo gene origination, and highlighted these de novo genes as a previously neglected source of human uniqueness in brain development.

## Results

### Key *cis* elements underpinning RNA nuclear export

**Fig. 1:**
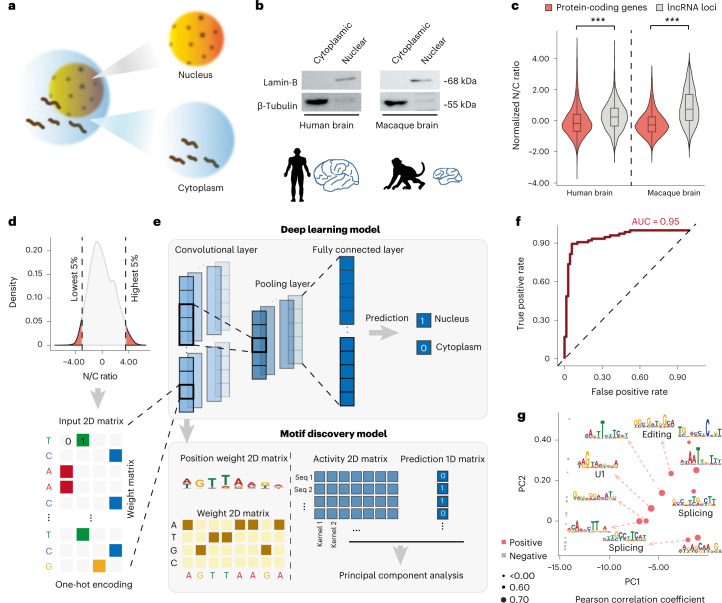
The U1 sequence as a key feature for predicting RNA nuclear retention. **a**, Overview of the experimental design. **b**, Western blots showing the protein expression of lamin-B and β-tubulin in nuclear and cytoplasmic fractions from human/macaque brain tissue. **c**, Distribution of normalized N/C ratios of mRNAs and lncRNAs in brain tissue. *n* = 15,734 mRNAs and 1,861 lncRNAs for human; *n* = 15,180 mRNAs and 2,719 lncRNAs for macaque; two-sided, unpaired Wilcoxon test, *P* < 2.2 × 10^−16^ and *P* < 2.2 × 10^−16^, respectively. The boxes represent interquartile range, with the line across the box indicates the median. The whiskers extend to the lowest and the highest values in the dataset. ***P ≤ 0.001. **d**, Sequences of transcripts excessively distributed in the nucleus or cytoplasm in HEK293T cells. **e**, Deep learning classification model to investigate the key *cis* elements underpinning the varied transcript nuclear export activity. **f**, Evaluation metrics, as well as features of prediction for the classification model, are shown. **g**, Key *cis* elements differentiating transcripts with varied nuclear export activity were identified by prioritizing the activation values in the first convolutional layer of this CNN network. Source data

**Extended Data Fig. 1:**
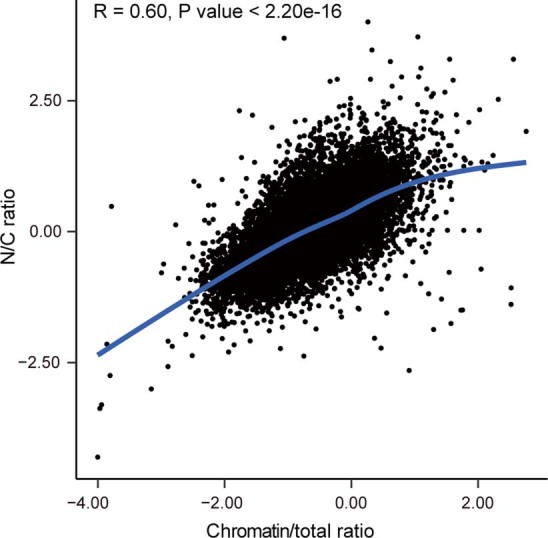
Comparison between Chromatin/total ratio and N/C ratio in quantifying levels of RNA nuclear export. Dotplot showing the correlation between the two parameters in quantifying the levels of nuclear export, on the basis of a public dataset (GSE100576). The Pearson correlation coefficient was shown with the P value. Two-sided, paired, t-test, P value < 2.2e-16.

**Extended Data Fig. 2:**
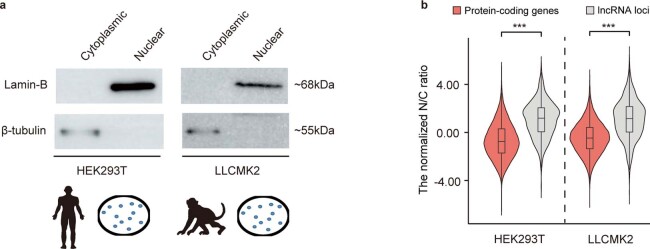
Subcellular distribution of mRNAs and lncRNAs in cell lines of human and rhesus macaque. **a**, Western blots showing the protein expression of lamin-B and β-tubulin in nuclear and cytoplasmic fractions from human and macaque cell lines. **b**, Distributions of the normalized N/C ratios of mRNAs and lncRNAs in HEK293T and LLCMK2 cells. Two-sided, unpaired Wilcoxon test, P value < 2.2e-16, < 2.2e-16, respectively. The boxes represent interquartile range, with the line across the box indicates the median. The whiskers extend to the lowest and the highest value in the dataset. ***P value ≤ 0.001.

While we and others have proposed the origination of human de novo genes from lncRNA loci, the detailed evolutionary process of this lncRNA–mRNA transition has yet to be fully delineated at the molecular level. Interestingly, although they share the similar transcript structure (the exon–intron structure, capped at 5′ ends and polyadenylated at 3′ ends) and transcriptional regulations, lncRNAs significantly enrich in the nucleus fraction relative to mRNAs^15,25^. Therefore, to address this issue from a spatial perspective, we first performed RNA sequencing (RNA-seq) studies on fractional brain tissues (Fig. 1a,b) from human and macaque, and introduced the N/C ratio (the ratio of reads in the nucleus to reads in the cytoplasm), a parameter showing efficiency comparable to the chromatin/total ratio in previous reports^54^ (Extended Data Fig. 1), to quantify the levels of nuclear export activity for each mRNA and lncRNA (Methods). Consistent with the previous findings, lncRNAs showed significantly higher N/C ratios than mRNAs in both species’ brain tissues, indicating the enrichment of lncRNAs in the nucleus (Fig. 1c; Wilcoxon test, *P* < 2.2 × 10^−16^). Similar results were found in cell lines of human and rhesus macaque (Extended Data Fig. 2).

On the basis of the sequences of transcripts excessively distributed in the nucleus or the cytoplasm (Fig. 1d), we then developed a deep learning classification model to investigate the key *cis* elements underpinning the nuclear export of transcripts (Methods). Briefly, a convolutional neural network (CNN) was developed with multiple convolutional/pooling layers and one fully connected layer (Fig. 1e), which efficiently predicted the nuclear export activity of transcripts with their sequences (area under the curve 0.95; Fig. 1f). We then extracted the key *cis* elements differentiating transcripts with varied nuclear export activity by prioritizing the activation values in the first convolutional layer of this CNN model. Notably, the existence of the U1 binding motif (recognized by the U1 small nuclear ribonucleoprotein) in the transcript was identified as the predominant element in predicting the localization of the transcripts (Fig. 1g). Some RNA splice-related sequences were also identified as informative *cis* elements in predicting the localization of the transcripts (Fig. 1g and Supplementary Table 1). These findings thus highlight the predominant contributions of these *cis* elements to the varied subcellular distributions of transcripts.

### Switching of key features in de novo gene origin

**Fig. 2:**
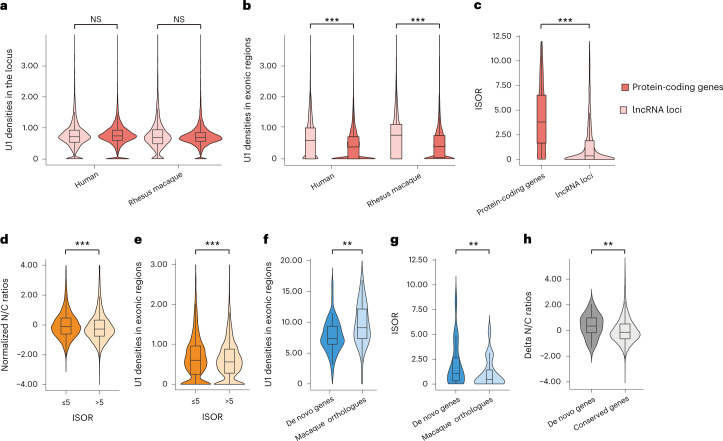
Switching of key features during the origin of human de novo genes. **a**,**b**, Box plots showing the density of strong U1 binding sites (in number of sites per kilobase) in the genic (**a**) and exonic regions (**b**) of genes encoding mRNAs and lncRNAs. *n* = 55,187 for human protein-coding genes; *n* = 2,615 for human genes encoding lncRNAs; *n* = 25,620 for macaque protein-coding genes; *n* = 616 for macaque genes encoding lncRNAs; statistics for **a**: one-sided, unpaired Wilcoxon test; statistics for **b**: one-sided, unpaired Wilcoxon test, *P* < 2.2 × 10^−16^ and *P* < 2.2 × 10^−16^, respectively. **c**, Distributions of ISOR scores for mRNAs and lncRNAs in the nuclear fraction of the human brain. *n* = 18,084 for mRNAs; *n* = 2,823 for lncRNAs; statistics for **c**: one-sided, unpaired Wilcoxon test, *P* < 2.2 × 10^−16^. **d**,**e**, Distributions of the normalized N/C ratio (**d**, *n* = 14,604 mRNAs; one-sided, unpaired Wilcoxon test, *P* < 2.2 × 10^−16^) and exonic U1 density (**e**, *n* = 14,604 mRNAs; one-sided, unpaired Wilcoxon test, *P* = 6.4 × 10^−6^) for mRNAs with different ISOR scores. **f**,**g**, Distributions of the density of all U1 binding sites (**f**, in number of sites per kilobase, *n* = 50 for de novo genes; *n* = 45 for their macaque orthologues encoding lncRNAs; one-sided, unpaired Wilcoxon test, *P* = 1.7 × 10^−3^) and ISOR scores (**g**, *n* = 19 pairs; one-sided, paired Wilcoxon test, *P* = 5.3 × 10^−3^), in de novo genes and their macaque orthologues encoding lncRNAs. **h**, Box plots showing the difference of N/C ratios between de novo genes and their macaque orthologues encoding lncRNAs in brain tissues. As we attempted to compare the de novo genes with the background, the differences of N/C ratios between orthologue pairs in macaque and human are shown. *n* = 32 for de novo genes; *n* = 12,210 for all orthologue pairs; one-sided, unpaired Wilcoxon test, *P* = 5.3 × 10^−3^. The boxes represent interquartile range, with the line across the box indicates the median. The whiskers extend to the lowest and the highest value in the dataset. **P ≤ 0.01; ***P ≤ 0.001; NS, not significant. Source data

We then investigated whether the distribution of these *cis* elements, especially the U1 binding sequences, could be used to explain the varied enrichment of subcellular localization of mRNAs and lncRNAs. Notably, although the density of U1 sequences was comparable between the loci encoding lncRNAs and the loci encoding mRNAs, the lncRNA transcripts showed a significantly higher exonic U1 density in both human (Wilcoxon test, *P* < 2.2 × 10^−16^) and rhesus macaque (*P* < 2.2 × 10^−16^) (Fig. 2a,b), indicating the involvement of RNA splicing in shaping this mRNA–lncRNA difference in the U1 density of the transcripts.

**Extended Data Fig. 3:**
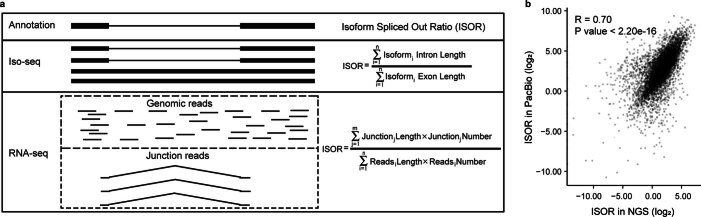
Quantifying the average splicing efficiency at whole-transcript level. **a**, Diagrams showing the formula in calculating ISOR according to RNA-seq and Iso-seq data, respectively. **b**, Dotplot showing the correlation between the ISOR scores calculated by the distribution of RNA-seq reads and those calculated by the distributions of Iso-seq reads, with the Pearson correlation coefficient shown with the P value. Two-sided, paired, t-test, P value < 2.2e-16.

To estimate the average degree of RNA splicing for each gene, we then defined the parameter isoform spliced-out ratio (ISOR) to calculate the ratio of the spliced out length to the exon length of the transcript from the RNA-seq data (Extended Data Fig. 3a and Methods). The ISOR score accurately quantified the average splicing efficiency at the whole-transcript level, as verified by comparison with the full-length transcript sequencing data of the same sample (Extended Data Fig. 3b and Methods). We then compared the ISOR scores for genes encoding mRNAs and lncRNAs and found that the protein-coding genes had significantly higher scores than those encoding lncRNAs, suggesting a higher splicing efficiency among mRNA transcripts in general (Fig. 2c; Wilcoxon test, *P* < 2.2 × 10^−16^). Notably, for mRNAs encoded by human protein-coding genes, the degree of RNA splicing efficiency is negatively correlated with the N/C ratio (Wilcoxon test, *P* < 2.2 × 10^−16^) and the exonic U1 density (Wilcoxon test, *P* = 6.4 × 10^−6^) for the corresponding transcripts (Fig. 2d,e). Taken together, it is plausible that RNA splicing could regulate the nuclear retention of the corresponding transcripts through the modulation of exonic U1 density, contributing to the differences between mRNAs and lncRNAs in nuclear export activity.

**Extended Data Fig. 4:**
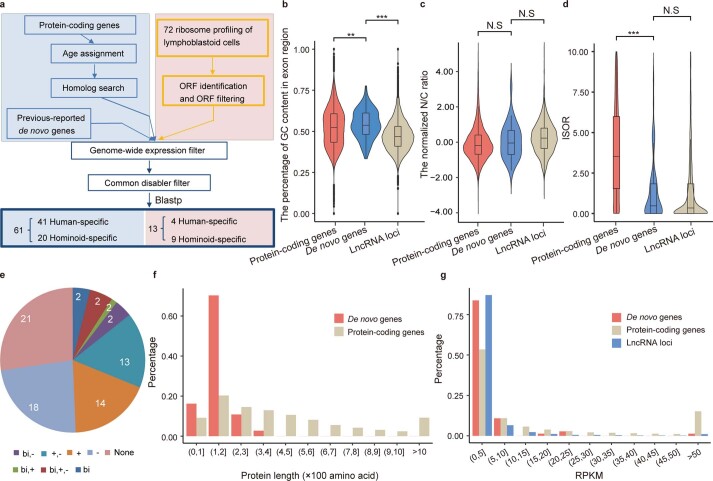
Identification of human-/hominoid-specific *de novo* genes. **a**, Computational pipeline for *de novo* gene identification. (**b-d**) The distributions of GC content (**b**, n = 21,392 for protein-coding genes; n = 74 for *de novo* genes; n = 7,109 for lncRNA loci; one-sided, unpaired Wilcoxon test, P value = 5.5e-3 and P value < 2.2e-16, respectively), N/C ratio (**c**, n = 15,734 for protein-coding genes; n = 33 for *de novo* genes; n = 1,860 for lncRNA loci; one-sided, unpaired Wilcoxon test, P value = 0.40, 0.16, respectively), and ISOR (**d**, two-sided, unpaired Wilcoxon test, P value < 2.2e-16, 0.94, respectively), for *de novo* genes (*De novo*, n = 61), protein-coding genes (Protein-coding, n = 17,990) and genes encoding lncRNAs (LncRNAs, n = 2,777). The boxes represent interquartile range, with the line across the box indicates the median. The whiskers extend to the lowest and the highest value in the dataset. **e**, The number of *de novo* genes co-opting with the transcriptional context of *cis*-NATs (Natural Antisense Transcripts) or bi-directional promoters were summarized and shown in pieplot. bi: bi-directional promoters; **+**: overlapping with known genes of same strand; **-**: overlapping with known genes of reverse strand. Here ‘overlapping a known gene’ means coordinate overlap, rather than an overlap with a known gene in the same frame. **f**, The distributions of the length of proteins, for *de novo* genes and protein-coding genes. **g**, The distributions of the expression levels for *de novo* genes, protein-coding genes and genes encoding lncRNAs. **P value ≤ 0.01; ***P value ≤ 0.001; N.S, not significant.

To investigate whether nuclear export activity increases along with the de novo gene origination from ancestral lncRNAs, we first updated the list of human- and hominoid-specific de novo genes on the basis of our previous studies^7,14^, by integrating additional de novo genes supported by new translational evidence from ribosome-profiling data and large-scale mass spectrometry (Extended Data Fig. 4a and Methods). Overall, 74 de novo genes were identified in human, including 45 genes encoding human-specific proteins and another 29 hominoid-specific genes encoding similar proteins in human and chimpanzee but not in rhesus macaque (Supplementary Table 2). The characteristics of these de novo genes, such as higher GC level, relatively smaller open reading frames (ORFs), lower expression levels and co-opting the transcriptional context of *cis* natural anti-sense transcripts (NATs) or bidirectional promoters, are consistent with previous reports^7,10,55^ (Extended Data Fig. 4b,e–g).

We then examined the degrees of RNA splicing, exonic U1 density and the nuclear export of the mRNAs encoded by these de novo genes, as well as those of the lncRNAs encoded by the macaque orthologues of these de novo genes, in the brain tissue of humans and rhesus macaques (Fig. 2f–h). Consistently, compared with the lncRNAs encoded by the macaque orthologues, the mRNAs encoded by these de novo genes showed significantly lower exonic U1 density (Wilcoxon test, *P* = 1.7 × 10^−3^; Fig. 2f) and higher ISOR values (Wilcoxon test, *P* = 5.3 × 10^−3^; Fig. 2g). Moreover, in contrast to the 12,210 conserved orthologue pairs between human and rhesus macaque as a background, transcripts encoded by the de novo genes showed a significantly decreased N/C ratio compared with those lncRNAs encoded by their macaque orthologues (Wilcoxon test, P = 5.3 × 10^−3^; Fig. 2h). Taken together, the switching of the degrees of RNA splicing, exonic U1 density and nuclear export appears to occur along with the process of de novo gene origination.

**Fig. 3:**
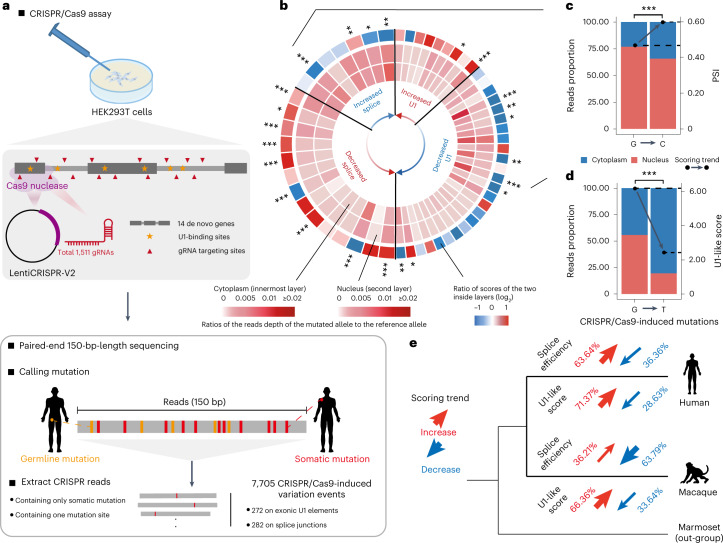
Exonic U1 elements directly regulate nuclear export in humans. **a**, Schematic of the CRISPR/Cas9 library design and targeted sequencing of de novo genes expressed in HEK293T cells. **b**, Mutations identified in U1/splice sites and their effects on the nuclear/cytoplasmic distribution of the corresponding transcript. The innermost layer and the second layer of the circular heat map show the ratios of the reads depth of the mutated allele to the reference allele in the nucleus (the second layer) and cytoplasm (the innermost layer), respectively. The ratio of scores of the two layers are shown on the outermost layer, in which the blue and red correspond to the odds ratio of N/C ratios between the mutants and the wild-type controls, respectively. According to the benchmark scale bar, the blue codes indicate that the mutation introduce a decreased N/C ratio for the corresponding transcript (increased nuclear export activity), while the red codes indicate that the mutations introduce an increased N/C ratio of the corresponding transcript (or decreased nuclear export activity). The mutations are ranked according to the differences in the U1 score (right part of the circular heat map) or the PSI value (left part) between the mutant and reference alleles (red arrow, mutants show higher U1 scores or lower PSI; blue arrow, mutants with lower U1 scores or higher PSI than the reference alleles). **c**,**d**, Proportions of reads in the nucleus and cytoplasm were shown in red and blue, respectively, for one mutation introducing a stronger splice site (**c**, two-sided, Fisher’s exact test, *P* < 2.2 × 10^−16^) and another mutation introducing a lower U1 score (**d**, two-sided, Fisher’s exact test, *P* < 2.2 × 10^−16^). **e**, The statistics of the segregating sites fixed after the divergence of human and rhesus macaques during the process of de novo gene origin. The effects of the segregating sites on the activity of RNA splicing and the affinity of U1 binding were predicted, and the proportions of sites with different effects are shown. *P ≤ 0.05; **P ≤ 0.01; ***P ≤ 0.001. Source data

**Extended Data Fig. 5:**
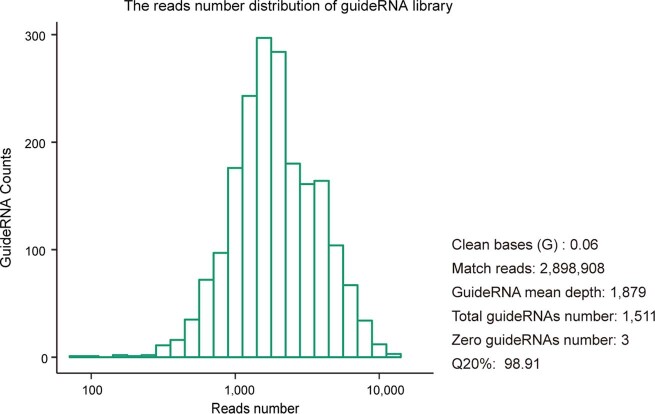
Reads dstribution of the gRNA library in this study. The gRNA library was subjected to deep sequencing for a quality control, with the statistics of the library, as well as the distribution of the coverage of the gRNAs shown.

To further clarify the causal relationship among RNA splicing, U1 regulation and nuclear export of these de novo genes, we designed a clustered regularly interspaced short palindromic repeats (CRISPR)/Cas9 library with 1,511 guide RNAs (gRNAs) to target splice junctions and exonic U1 sequences on 14 multiple-exon de novo genes moderately expressed in HEK293T cells (fragments per kilobase of transcript per million mapped fragments (FPKM) >0.5). These gRNAs introduced random mutations at these sites (Methods, Fig. 3a, Extended Data Fig. 5 and Supplementary Table 3). After transfection, the nuclear and cytoplasmic fractions of these de novo genes were then polymerase chain reaction (PCR) amplified and subjected to deep sequencing (Methods). Overall, we identified 7,705 CRISPR/Cas9-induced mutations: 272 were located on exonic U1 sites, and 282 on splice junctions.

We then characterized the effects of these mutations on the nuclear export of the corresponding transcripts, by comparing the distributions of the RNA-seq reads with the reference allele or mutation allele in the nuclear and cytoplasmic fractions (Methods and Supplementary Table 4). Generally, in contrast to the distribution of the reference alleles, mutations leading to weaker U1 sites increased the cytoplasmic localization of the corresponding transcripts, while mutations leading to stronger U1 sites increased the nuclear localization (Fig. 3b and Supplementary Table 5). Moreover, when calculating and comparing the splicing efficiencies (in per cent spliced in, PSI) of the reference and mutation-containing transcripts, we identified 7 mutations that increased and 14 that decreased the splicing activity of the corresponding transcripts (Methods). Among these sites, 13 sites significantly changed the localization of the corresponding transcripts, with 10 (or 76.9%) supporting the direct regulation of the splicing efficiency on RNA nuclear export, in that mutations leading to stronger splice sites had significantly increased their cytoplasmic localization, while mutations leading to weaker splice sites decreased their cytoplasmic localization of the corresponding transcripts (Fig. 3b and Supplementary Table 5). Two examples with a mutation-induced change in nuclear or cytoplasmic localization are shown in Fig. 3c,d.

Finally, to understand the genetic background underlying such a lncRNA–mRNA transition in de novo gene origination, we identified the segregating sites on their loci that were fixed after the divergence of humans and rhesus macaques, and predicted their effects on the activity of RNA splicing and the affinity of U1 binding (Methods). Interestingly, after the divergence of human and rhesus macaque, most of the segregating sites accumulated in the human lineage led to stronger splice sites (7 of 11, or 63.6%), while most of the segregating sites accumulated in the macaque lineage led to weaker splice sites (37 of 58, or 63.8%) (Fig. 3e). In addition, most of the segregating sites accumulated in human (177 of 248, or 71.4%) or macaque lineages (874 of 1,317, or 66.4%) led to stronger U1 binding, verifying the hypothesis that the U1 elements may function as an intermediate effector to differentiate mRNAs and lncRNAs in terms of subcellular localization, by connecting the regulation of RNA splicing with the regulation of RNA nuclear export (Fig. 3e).

Taken together, these findings suggest that RNA splicing can efficiently regulate the nuclear export of the corresponding transcripts, partially through the removal of intronic U1 sequences, a process contributing substantially to the origin of de novo genes during the recent human evolution.

### A selectively constrained boundary in de novo gene origin

To address whether the differences in sequence and expression profiles of lncRNAs explain why de novo genes originate from some lncRNAs but not others, we investigated the orthologous lncRNA loci of de novo genes in rhesus macaques as a proxy for determining ancestral status, assuming that the features of these loci have remained unchanged in the macaque lineage since their divergence from the human lineage.

**Fig. 4:**
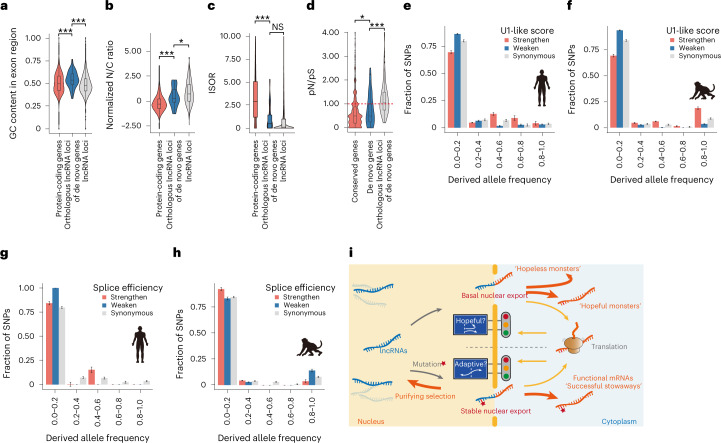
Nuclear export is a selectively constrained boundary differentiating mRNAs from lncRNAs. **a**–**c**, Estimated features of ancestral lncRNAs for the de novo genes identified using their macaque orthologues encoding lncRNAs. The distributions of GC content (**a**), N/C ratio (**b**) and splice efficiency (**c**) of these lncRNAs are summarized in box plots and compared with those of the genome-wide lncRNAs or protein-coding genes in macaques. Statistics for **a**: *n* = 25,211 for macaque protein-coding genes; *n* = 71 for macaque orthologues of human de novo genes; *n* = 4,370 for macaque lncRNAs; one-sided, unpaired Wilcoxon test, *P* = 2.5 × 10^−7^ and *P* = 3.0 × 10^−13^, respectively; statistics for **b**: *n* = 11,703 for macaque protein-coding genes; *n* = 34 for macaque orthologues of human de novo genes; *n* = 2,719 for rhesus macaque lncRNAs; one-sided, unpaired Wilcoxon test, *P* = 2.6 × 10^−5^ and *P* = 2.6 × 10^−2^; statistics for **c**: *n* = 13,463 for macaque protein-coding genes; *n* = 19 for macaque orthologues of human de novo genes; *n* = 3,702 for macaque lncRNAs; one-sided, unpaired Wilcoxon test, *P* = 8.0 × 10^−6^ and *P* = 7.4 × 10^−2^. **d**, pN/pS scores in human population are summarized in box plots, for de novo genes, the macaque orthologues of these de novo genes, as well as the protein-coding genes conserved in human and macaque as a control. *n* = 13,131 for conserved genes between human and macaque; *n* = 41 for de novo genes; *n* = 60 for macaque orthologues of human de novo genes; one-sided, unpaired Wilcoxon test, *P* = 4.7 × 10^−2^ and *P* = 8.4 × 10^−4^. The boxes represent interquartile range, with the line across the box indicates the median. The whiskers extend to the lowest and the highest value in the dataset. **e**,**f**, Derived allele frequency spectra for the human polymorphic sites that led to stronger (Strengthen, *n* = 78 SNPs) or weaker U1 sites (Weaken, *n* = 175 SNPs) in de novo genes (**e**), as well as the macaque polymorphic sites that led to stronger (Strengthen, *n* = 119 single-nucleotide polymorphisms (SNPs)) or weaker U1 sites (Weaken, *n* = 307 SNPs) in macaque orthologues of these de novo genes (**f**). **g**,**h**, Derived allele frequency spectra for the human polymorphic sites that introduce stronger (Strengthen, *n* = 10 SNPs) or weaker splice sites (Weaken, *n* = 28 SNPs) in de novo genes (**g**), as well as the macaque polymorphic sites that introduce stronger (Strengthen, *n* = 28 SNPs) or weaker splice sites (Weaken, *n* = 44 SNPs) in the macaque orthologues of de novo genes (**h**). Derived allele frequency spectra for synonymous sites (Synonymous, *n* = 130 SNPs and 268 SNPs) in human and macaque are also shown as neutral controls; error bars, standard deviations (s.d.) estimated by 1,000 bootstrap replicates. Data are presented as mean ± s.d. *P ≤ 0.05; ***P ≤ 0.001; NS, not significant. **i**, A ‘successful stowaway’ model for the origin of functional de novo genes from lncRNA loci.

Compared with the genome-wide lncRNA loci and protein-coding genes as the backgrounds, both the de novo genes and their macaque orthologues encoding lncRNAs displayed extreme features of high GC content (Wilcoxon test, *P* = 5.5 × 10^−3^, *P* < 2.2 × 10^−16^, *P* = 2.5 × 10^−7^ and *P* = 3.0 × 10^−13^, for de novo genes and their macaque orthologues in contrast to the genome-wide protein-coding genes or lncRNA-encoding genes, respectively; Fig. 4a and Extended Data Fig. 4b). This observation suggests the involvement of the pre-adaptation model in the origination process. On the other hand, both the de novo genes and their macaque orthologues encoding lncRNAs showed intermediate level of N/C ratio and ISOR in comparison with the genome-wide lncRNAs and mRNAs (Fig. 4b,c and Extended Data Fig. 4c,d), suggesting the involvement of the continuum model and the roles of splice-directed nuclear export as a key step in de novo gene origin. Moreover, we found that the ratios of pN to pS in these de novo genes were generally less than 1, which are significantly lower than those of the lncRNA orthologues of these de novo genes in rhesus macaque (Wilcoxon test, *P* = 8.4 × 10^−4^), and higher than those of the protein-coding genes conserved in human and macaque (Fig. 4d; Wilcoxon test, *P* = 0.047). It is thus plausible that these de novo genes are generally selectively constrained, with a few de novo genes under strong selection, a substantial proportion of these genes under relatively weaker selection and others under no selection.

Overall, it seems that the de novo genes acquired gene-like features and biological functions along with their origination. A selection boundary underpinning this pre-adaptive mode of origin should thus, in principle, exist. As we had linked nuclear export activity and *cis* elements such as RNA splice junctions and U1 sequences to the origin of de novo genes, we then performed a population genetics study in humans and macaques to investigate whether they may function as a selection boundary underlying these features of pre-adaptation. Interestingly, the polymorphic sites weakening the U1 binding sites had an excess of low-frequency variants in the orthologous lncRNA loci of de novo genes, showing a significantly left-skewed frequency spectrum of the derived alleles than that of the synonymous sites as a neutral control (Fig. 4e,f, Monte Carlo *P* = 9.2 × 10^−2^ and *P* < 1.0 × 10^−4^ for human de novo genes and macaque lncRNA loci, respectively). In contrast, the selection pressure on a splice site is directional according to the RNA species, in that the polymorphic sites weaken the splice junctions in mRNAs encoded by the de novo genes, or strengthening those in the lncRNAs encoded by the macaque orthologues of these de novo genes, had an excess of low-frequency variants (Fig. 4g,h, Monte Carlo *P* < 1.0 × 10^−4^ and *P* = 0.049, for human de novo genes and macaque lncRNA loci, respectively). These findings are in line with the model that the RNA nuclear export acts a selectively constrained boundary between mRNAs and lncRNAs. The new functional genes thus represent ‘successful stowaways’ actively passing through it, showing a mode of pre-adaptive origin in that they acquire functions along with the achievement of their coding potential (Fig. 4i and Discussion).

### A de novo gene regulates human cortical development

**Fig. 5:**
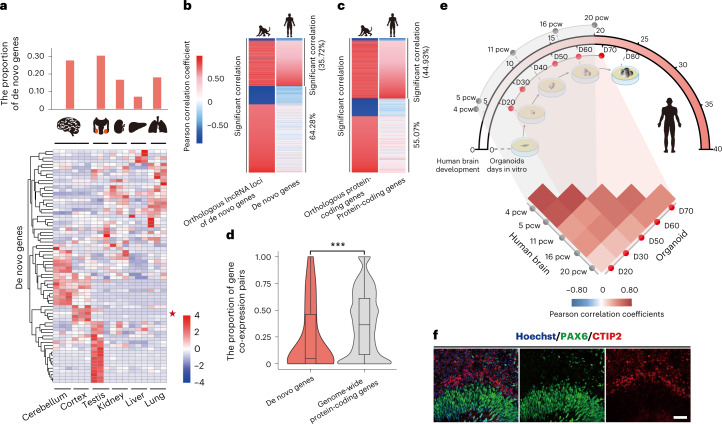
Newly originated de novo genes modulate the human brain transcriptome. **a**, Heat map showing the tissue expression profile of 72 de novo genes in six human tissues (brain, kidney, liver, lung and testis), with the case gene (*ENSG00000205704*) selected in the proof-of-concept study marked with a star. Two de novo genes (*ENSG00000233290* and *ENSG00000205056*) not expressed in all of the six types of tissue were not included. **b**–**d**, For each macaque gene co-expressed with the macaque orthologues of human de novo genes (**b**, left), we investigated whether such a co-expressed gene pair is conserved in human (**d**, right), with the Pearson correlation coefficient between gene pairs shown in the heat maps according to the colour scales. The list of genome-wide protein-coding genes was also shown as a control (**c**). For each de novo gene, the proportions of the co-expressed gene pairs conserved in human and rhesus macaque were also summarized in the box plot, which were compared with those of the control (**d**, *n* = 42 for de novo genes; *n* = 14,226 for conserved genes between human and macaque; two-sided, paired Wilcoxon test, *P* = 2.8 × 10^−4^), The boxes represent interquartile range, with the line across the box indicating the median. The whiskers extend to the lowest and the highest value in the dataset. **e**, Schematic plot showing the temporal relationship between the stages of human cortical organoid culture and human foetal brain development, with the correlations of the transcriptome summarized in the heat map. The Pearson correlation coefficients between the transcriptomes of brain tissues and cortical organoids at different stages are shown in the heat map according to the scales. The dotted line highlights the comparisons between pairs of tissue/organoid at comparable developmental stages. 4–20 pcw: post-conceptional weeks 4–20 in early human brain development; D20–70: RNA-seq assays were applied to samples of at four timepoints (protocol days of human cortical organoids 20–70). **f**, Immunofluorescence staining of PAX6 (green) and CTIP2 (red) in Hoechst-stained (blue) organoids generated from hESCs. Scale bar, 50 μm. The experiment was repeated three times independently with similar results. ***P ≤ 0.001.

Given the finding that these de novo genes are selectively constrained in general (Fig. 4d), we then investigated their definite functions. Consistent with previous reports, these de novo genes showed brain- and testis-enriched transcriptional expression (Fig. 5a). We then focused on the brain to investigate the effects of these new genes on the human transcriptome, by comparing the cross-species conservation of the correlated genes at the population level. Briefly, on the basis of the transcriptome data from the brains of 35 macaque animals, we identified genes with significant expression correlation with the macaque orthologues of the human de novo genes, and further developed a gene co-expression network. We then investigated the degree of conservation of the network in humans on the basis of transcriptome data from the brains of 134 human individuals. Notably, compared with the conserved genes showing high degrees of cross-species conservation in the gene co-expression network, the conservation level was significantly lower for the young de novo genes with recent lncRNA–mRNA switching after the divergence between human and rhesus macaque (Fig. 5b–d; Wilcoxon test, *P* = 2.8 × 10^−4^). It is thus possible that at least a portion of these de novo genes have acquired new regulatory functions to shape the gene network during the human brain evolution.

To further clarify the biological functions of these newly originated genes, especially in brain development, we employed neural differentiation and cortical organoid systems with human embryonic stem cells (hESCs) to determine whether the new gene could regulate human cortical development (Fig. 5e, top). Notably, the cortical organoids we developed (grown for 60 days) showed tissue-like structures of the developing brain, in that PAX6-positive RGCs and CTIP2-positive neurons could be clearly concentrated in two distinguishable layers, corresponding to the ventricular zone (VZ) and the cortical plate (CP) of a developing neocortex (Fig. 5f). In addition, we generated the transcriptomes of hESC-derived cortical organoids at different stages (days 20, 30, 50, 60 and 70), and compared them with the transcriptome data from the corresponding stages of human brain development (post-conceptional weeks 4, 5, 11, 16 and 20). The transcriptome data in the organoid stages and those of human brain development were well correlated (Fig. 5e, bottom). These findings thus suggest that the cortical organoid system could be used to adequately mimic the early development of the human neocortex and to clarify the functions of de novo genes through the gene knock-out approach.

**Fig. 6:**
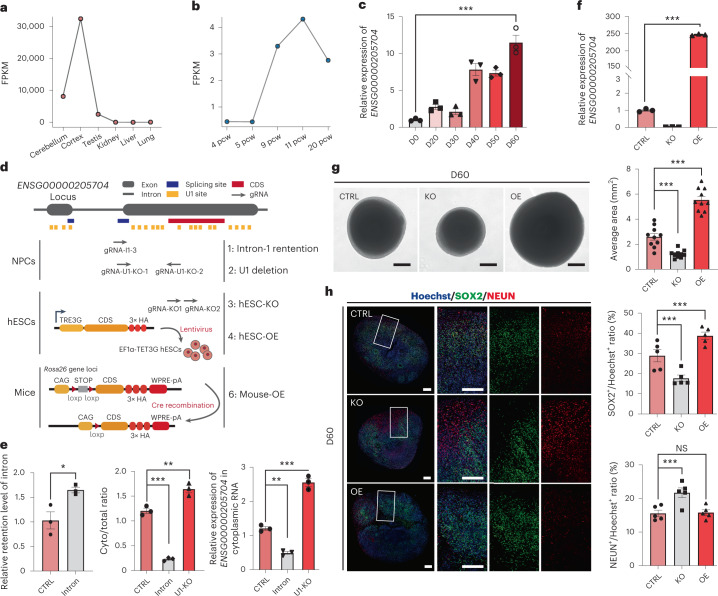
A hominoid-specific de novo gene regulates neuronal maturation. **a**–**c**, The expression profiles of ENSG00000205704 in human tissues (**a**, relative expression), human foetal brain at different developmental stages (**b**, FPKM) and human cortical organoids at different protocol days (**c**, relative expression, *n* = 3 biologically independent experiments, with a mixture of over ten organoids; data are presented as mean ± standard error of the mean (s.e.m.); two-sided, unpaired Student’s *t*-test, *P* = 5.0 × 10^−4^). 4–20 pcw: post-conceptional weeks 4–20 in early human brain development; D0–60: protocol days 0, 20, 30, 40, 50 and 60. **d**, Overview of the gene-editing studies on *ENSG00000205704* in hNPCs, hESCs and transgenic mice. The CRISPR/Cas9 gRNAs were designed to target the splice site (Intron-1 retention), U1 binding sites (U1 deletion) and the coding sequence (CDS) regions (hESC-KO) of ENSG00000204705. hESCs with the over-expression of ENSG00000205704 were also developed through lentivirus transfection in hESCs (hESC-OE). Transgenic mice with ENSG00000205704-knock-in were constructed by CRISPR/Cas9 and the ectopical expression was manipulated by Cre recombination (Mouse-OE). **e**, Left: the effects of the disruptions of the splice site of ENSG00000205704 (Intron) on the levels of intron retention were quantified, compared with the wild type (CTRL). Middle: the nuclear export efficiencies (Cyto/total ratio: the proportion of transcripts located in cytoplasm) of ENSG00000205704 were quantified in wild-type hNPCs (CTRL), in hNPCs with the disruption of the splice site of ENSG00000205704 (Intron) and in hNPCs with six U1 binding sites on the second exon of ENSG00000205704 removed (U1-KO). Right: the expression levels of ENSG00000205704 in cytoplasm for different samples. *n* = 3 biologically independent experiments, data are presented as mean ± s.e.m. Left: two-sided, unpaired Student’s *t*-test, *P* = 1.5 × 10^−3^; middle: one-way ANOVA, *P* < 1.0 × 10^−4^; Dunnett’s multiple comparisons test, Intron versus CTRL *P* < 1.0 × 10^−4^, U1-KO versus CTRL *P* = 2.1 × 10^−3^; right: one-way ANOVA, *P* < 1.0 × 10^−4^; Dunnett’s multiple comparisons test, Intron versus CTRL *P* = 1.0 × 10^−3^, U1-KO versus CTRL *P* < 1.0 × 10^−4^. **f**, The relative expression levels of ENSG00000205704 in hESC-KO and hESC-OE organoids cultured for 60 days (*n* = 3, data are presented as mean ± s.e.m.; two-sided unpaired Student’s *t*-test, *P* < 1.0 × 10^−4^; this experiment was repeated three times independently with similar results). **g**, Left: brightfield images showing the size of organoids generated from hESC (CTRL), hESC-KO and hESC-OE. D60: protocol day 60. Scale bars, 500 μm. Right: quantification of the average area of organoids, according to the brightfield images, *n* = 10, data are presented as mean ± s.e.m.; one-way ANOVA, *P* < 1.0 × 10^−4^; Dunnett’s multiple comparisons test, KO versus CTRL *P* = 5.6 × 10^−4^, OE versus CTRL *P* < 1.0 × 10^−4^. **h**, Left: immunofluorescence staining of SOX2 (green) and NEUN (red) in Hoechst-stained (blue) organoids, grown for 60 days from hESCs (CTRL), hESC-KO and hESC-OE. Scale bars, 200 μm. Right: quantification of the immunofluorescence stainings. *n* = 5 organoids, data are presented as mean ± s.e.m.; top: one-way ANOVA, *P* = 1.0 × 10^−4^; Dunnett’s multiple comparisons test, KO versus CTRL *P* = 8.1 × 10^−3^, OE versus CTRL *P* = 1.8 × 10^−2^; bottom: one-way ANOVA, *P* = 3.0 × 10^−3^; Dunnett’s multiple comparisons test, KO versus CTRL *P* = 4.0 × 10^−3^, OE versus CTRL *P* = 9.9 × 10^−1^. *P ≤ 0.05; ***P ≤ 0.001; NS, not significant.

**Extended Data Fig. 6:**
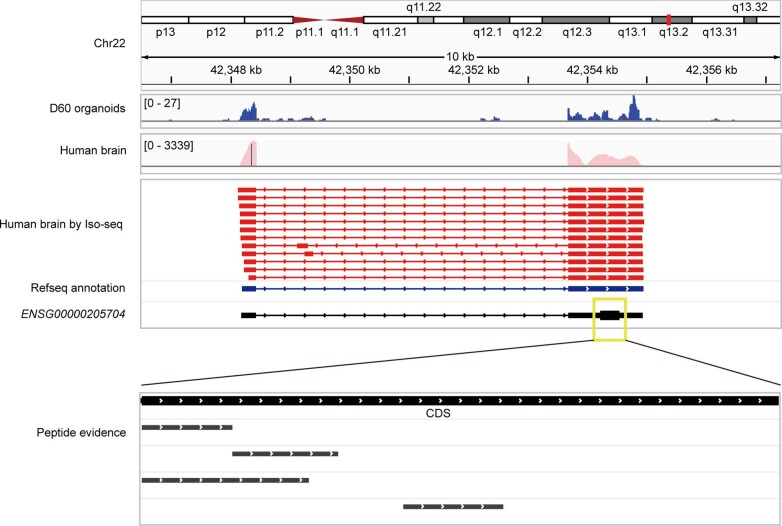
The expression of *ENSG00000205704* in human. The gene structure of *ENSG00000205704* was shown, with the items of evidence supporting the transcriptional and translational expression of this new gene aligned accordingly, including the reads density of RNA-seq data of the cortical organoids grown for 60 days (D60 organoids, this study), the reads density of cytoplasmic RNA-seq data of human brain (Human Brain, this study), the Iso-seq reads of human brain (Human Brain by Iso-seq, the same dataset used in validation of ISOR), and the peptides identified by large-scale mass spectrometry (Peptide Evidence, retrieved from PRIDE, PeptideAtlas, ProteomicsDB and Human Proteome Map database).

**Extended Data Fig. 7:**
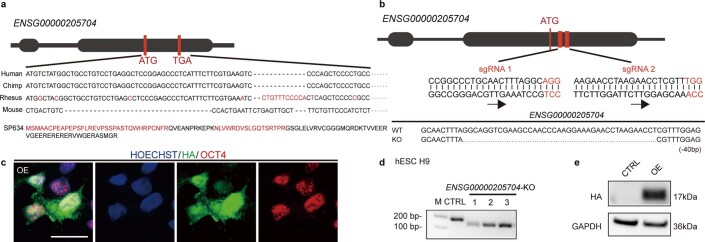
Functional study of *ENSG00000205704* in hESCs. **a**, Multiple alignment of the CDS region of *ENSG00000205704* in human with the orthologous regions in chimpanzee, rhesus macaque and mouse. The protein sequence encoded by *ENSG00000205704* in human was shown, with the start and stop codons highlighted in the diagram of the gene. **b**, The design of CRISPR/Cas9 assay to knock-out *ENSG00000205704*, with the sequences and the target regions of the gRNAs shown. The *ENSG00000205704*-KO hESC line was further verified by Sanger sequencing, as shown below the diagram. **c**, Immunofluorescence staining of HA tag (HA, indicative of the expression of ENSG00000205704 protein with HA tag) and OCT4 (OCT4) in hESCs with the overexpression of *ENSG00000205704* (OE). Scale bars, 20 μm. This experiment was repeated 5 times independently with similar results. **d**, PCR validation of the knock-out assay. CTRL, wild type hESCs; *ENSG00000205704-*KO, three replicates of *ENSG00000205704*-KO hESCs with the target region removed on both chromosomes. **e**, Western blots showing the expression of the HA-tagged ENSG00000205704 protein in wide type hESCs (CTRL**)**, and the hESCs with the over expression of the HA-tagged ENSG00000205704 protein (OE), This experiment was repeated 3 times independently with similar results.

As a proof of concept, we investigated the impact of RNA splicing and U1 recognition sites on one of these newly originated, hominoid-specific de novo genes, *ENSG00000205704*, which encodes a putative protein of 107 amino acids located in both cytoplasm and nucleus (Extended Data Figs. 6 and 7a). The gene is highly expressed in human brain tissues (Fig. 6a) and showed an increased expression during the development of both human brains and the cortical organoids (Fig. 6b,c). We first attempted to clarify the direct regulation of RNA splicing and U1 recognition sites on the nuclear export efficiency of this gene. To this end, we developed two CRISPR/Cas9 arrays to disrupt the splice site or U1 recognition sites on this gene in human neural progenitor cells (hNPCs, Fig. 6d). Notably, in hNPCs with disrupted splice sites of ENSG00000205704, the splice efficiency of ENSG00000205704 was significantly decreased (Student’s *t*-test, *P* = 1.5 × 10^−3^; Fig. 6e, left), and the nuclear export levels of the transcript was decreased accordingly (one-way analysis of variance (ANOVA) test, *P* < 1.0 × 10^−4^; Fig. 6e, middle), in that more transcripts expressed in mutant cells were nuclear localized (one-way ANOVA test, *P* = 1.0 × 10^−3^; Fig. 6e, right). Meanwhile, when one U1-enriched region at the second exon of ENSG00000205704 was removed via CRISPR/Cas9 assay (Fig. 6d), we detected a significantly increased nuclear export level for the corresponding transcript (one-way ANOVA test, *P* = 2.1 × 10^−3^; Fig. 6e, middle), and a subsequent increased cytoplasmic expression of this transcript (one-way ANOVA test, *P* < 1.0 × 10^−4^; Fig. 6e, right). These findings thus strengthened the direct regulation of these *cis* elements on the nuclear export efficiency of this gene.

**Extended Data Fig. 8:**
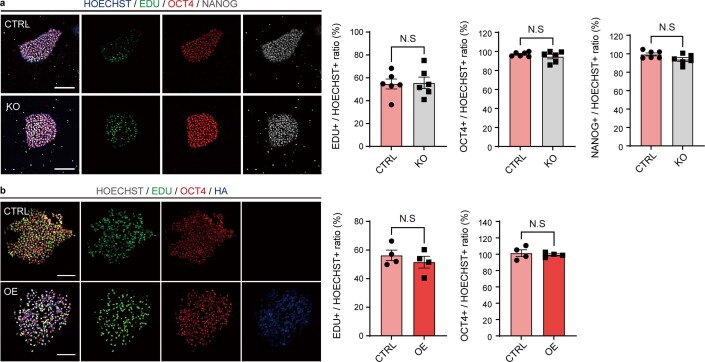
Effects of *ENSG00000205704* knock-out and over expression on hESC pluripotency. **a**, Left panel, Immunofluorescence staining of marker genes of hESC pluripotency for wild type hESCs (CTRL) and hESCs with *ENSG00000205704* knock-out (KO). Right panel, quantifications of the immunofluorescence staining. Scale bars, 100 μm; n = 6 biologically independent clones; data are presented as mean values + /- SEM. **b**, Left panel, Immunofluorescence staining of marker genes of hESC pluripotency for wild type hESCs (CTRL) and hESCs with *ENSG00000205704* over expression (OE). Right panel, quantifications of the immunofluorescence staining. Scale bars, 100 μm; n = 4 biologically independent clones; data are presented as mean values + /- SEM; two-sided, unpaired t-test. N.S, not significant.

We then clarified the regulatory roles of this new gene on neurogenesis and human brain development in the human cortical organoid system, showing a moderate expression of this gene (Fig. 6c). To this end, we developed hESCs with over-expression of *ENSG00000205704* (hESC-OE), and genetically engineered hESCs with CRISPR/Cas9-mediated knock-out of *ENSG00000205704* (hESC-KO) to investigate the effect of *ENSG00000205704* over-expression and depletion on the development of human cortical organoids (Fig. 6f, Extended Data Fig. 7b–e and Methods). While the over-expression and depletion of this new gene had no significant effects on the pluripotency of hESCs (Extended Data Fig. 8), the size of the organoids grown from hESC-KO was significantly decreased, in contrast to organoids grown from the wild-type hESCs at the corresponding development periods (Fig. 6g; one-way ANOVA test, *P* = 5.6 × 10^−4^). Meanwhile, the size of the organoids grown from hESC-OE was significantly increased, in contrast to the wild type (Fig. 6g; one-way ANOVA test, *P* < 1.0 × 10^−4^). We then performed immunofluorescence staining with cell-type-specific markers to investigate whether the changes in the cell composition of organoids could contribute to the varied organoid size grown for 60 days. Overall, compared with the organoids grown from wild-type hESCs, the proportion of SOX2-positive cells, indicative of RGCs, was significantly decreased in organoids grown from hESC-KO (Fig. 6h; one-way ANOVA test, *P* = 8.1 × 10^−3^), while the proportion of the cells marked by NEUN, a nuclear neuronal marker indicative of mature neurons, was significantly increased (Fig. 6h; one-way ANOVA test, *P* = 4.0 × 10^−3^). Meanwhile, the proportion of SOX2-positive cells was significantly increased (Fig. 6h; one-way ANOVA test, P = 1.8 × 10^−2^) in organoids grown from hESC-OE.

**Extended Data Fig. 9:**
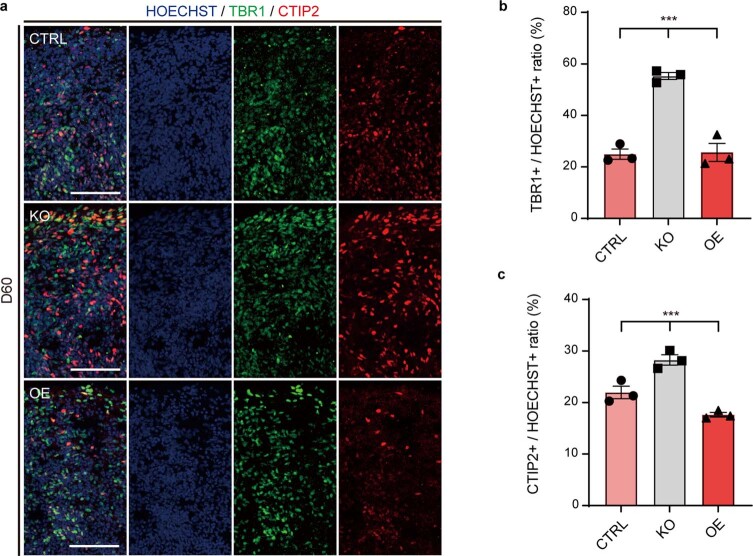
The distribution of cortical layer-specific neurons in organoids. **a**, Immunofluorescence staining of TBR1 (red) and CTIP2 (green) in Hoechst-stained (blue) cortical organoids grown for 60 days from wild type hESCs (CTRL), hESCs with *ENSG00000205704*-KO (KO), and hESCs with the over expression of *ENSG00000205704* (OE). Scale bars, 100 μm. **b**, Quantifications of the immunofluorescence staining for TBR1 in CTRL, KO and OE; n = 3 biologically independent organoids; data are presented as mean values + /- SEM; one-way ANOVA, P value = 1.8e-4; Dunnett’s multiple comparisons test, KO vs CTRL P value = 2.4e-4, OE vs CTRL P value = 9.7e-1. **c**, Quantifications of the immunofluorescence staining for CTIP2 in CTRL, KO and OE; n = 3 biologically independent organoids; data are presented as mean values + /- SEM. One-way ANOVA, P value = 6.2e-4; Dunnett’s multiple comparisons test, KO vs CTRL P value = 5.8e-3, OE vs CTRL P value = 3.1e-2. ***P value ≤ 0.001.

Moreover, as cortical neurons are located in six layers and emerged following a temporal order during the cortical development, we further investigated the changes of the proportions of neurons at different cortical layers. Notably, the proportions of both the layer VI neurons (as marked by TBR1) and the layer V neurons (as marked by CTIP2) changed significantly in organoids grown for 60 days from hESC-KO or hESC-OE, respectively, compared with the wild type, supporting the regulatory roles of *ENSG00000205704* in the maintenance of progenitor pool and the maturation of neurons at different cortical layers (Extended Data Fig. 9; one-way ANOVA test, *P* = 1.8 × 10^−4^ and 6.2 × 10^−4^, for TBR1 and CTIP2, respectively).

**Extended Data Fig. 10:**
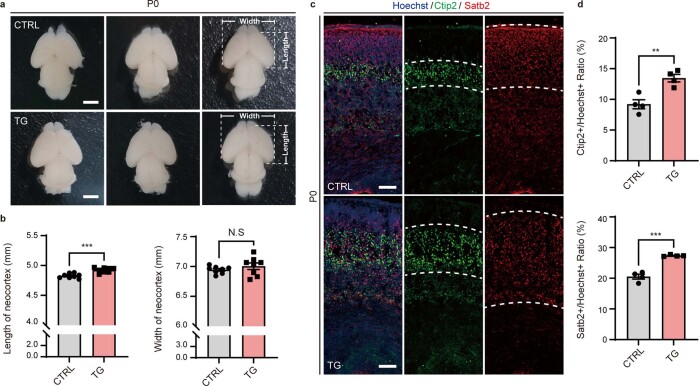
*In vivo* function of *ENSG00000205704* in cortical development. (**a-b**) Representative pictures of the brains from wild-type mice (CTRL) and the transgenic mice with the overexpression of *ENSG00000205704* (TG) were shown (**a**), with the length and the width of the neocortex in CTRL and TG quantified and compared (**b**, n = 8 mice, data are presented as mean values + /- SEM; left, two-sided, unpaired t-test, P value = 2.9e-4; Right, two-sided, unpaired t-test, P value = 3.2e-1). **c**, Immunofluorescence staining of Ctip2- and Satb2-marked regions, indicative of regions with deep-layer neurons and upper-layer neurons as marked in the figure, respectively. **d**, The proportions of Ctip2-positive (upper panel) and Satb2-positive cells (lower panel) in CTRL and TG were quantified, normalized, and compared. n = 4 mice; data are presented as mean values + /- SEM; upper panel, two-sided, unpaired t-test, P value = 4.7e-3; lower Panel, two-sided, unpaired t-test, P value = 1.4e-4. **P value ≤ 0.01; ***P value ≤ 0.001; N.S, not significant.

To further investigate the in vivo functions of *ENSG00000205704* in cortical development, we generated transgenic mice with ectopical expression of the ORF of *ENSG00000205704* (Fig. 6d). Notably, the transgenic mice showed significantly enlarged brains than the wild types in the length of neocortex (P0 stage, Extended Data Fig. 10; Student’s *t*-test, *P* = 2.9 × 10^−4^) but not in the width of neocortex (Extended Data Fig. 10; Student’s *t*-test, P = 3.2 × 10^−1^), and significant cortical expansion was detected, by the immunofluorescence stainings of Ctip2- and Satb2-marked regions indicative of regions with deep-layer neurons and upper-layer neurons, respectively (Extended Data Fig. 10; Student’s *t*-test, *P* = 4.7 × 10^−3^ and 1.4 × 10^−4^).

Taken together, the organoids grown from hESC-KO appeared to develop and mature at a quicker pace, leading to a significantly decreased size of the organoids during the same developmental periods, while both the organoids grown from hESC-OE and the transgenic mice with ectopically expressed *ENSG00000205704* exhibit a delayed neuronal maturation and a subsequent cortical expansion, substantiating the direct contribution of this newly originated protein to human brain development^53^.

## Discussion

Although recent studies have reported the enrichment of lncRNAs in nucleus^15,56–59^, the involvement of U1 elements in RNA nuclear retention^16,24^ and the possible contributions of RNA splicing to the nuclear export of transcripts^18–20^, the general mechanisms underpinning the varied subcellular localization of mRNAs and lncRNAs remains largely elusive. To this end, Gudenas and Wang^60^ reported a computational model and proposed the contributions of *cis* elements to the subcellular localizations of lncRNAs, while no significantly enriched *cis* element was reported. Here we performed a more comprehensive study to investigate the correlations and causal relationships among these regulations, on the basis of combined strategies of subcellular fractional experiments, deep learning classification model, unbiased identification of *cis* elements accounting for RNA nuclear export and experimental verification of the causal relationship with CRISPR/Cas9. On the basis of this robust model, we identified the dominant *cis* elements, those of U1 and RNA splicing-associated elements, underpinning the varied subcellular localization of transcripts. Notably, Azam et al.^24^ and Yin et al.^16^ also implicated the U1 elements in the regulation of the subcellular localization of transcripts from the perspective of molecular experiments, independently supporting the performance of the deep learning approach in this study. The identification of de novo genes with a recent lncRNA–mRNA transition and the key *cis* elements underpinning RNA subcellular localization constitutes a basis for further investigating the driver mechanisms underlying the de novo gene origin.

In this study, we focused on the changes of *cis* regulations in driving the origin of de novo genes via actively passing through the nuclear export boundary. Notable, some cytosol-localized lncRNAs with non-coding functions should also have evolved nuclear export capabilities, possibly through the mechanisms of both *cis* regulations and *trans* regulations by RNA-binding proteins. When investigating the N/C ratio and translational efficiency for the functional lncRNAs as summarized in Statello et al.^25^, we found cases of lncRNAs with relatively active nuclear export but low translational efficiency (for example, *NORAD* and *TINCR*). Several pilot studies reporting contributions of specific sequence features to polysome binding may provide evidence to explain such a phenomenon^61–63^. These mechanisms represent a possible translational boundary against the translation of these cytosol-localized lncRNAs with non-coding functions. However, aside from these lncRNAs with specific non-coding functions in cytoplasm, RNA-binding proteins such as NXF1 and CRM1 have been reported to facilitate the nuclear export for other lncRNAs^25,64–66^, and more than 70% of cytoplasmic-existing lncRNAs are reported to be available to the translation machinery for protein translation at relatively lower rates^25,30,61^. These findings thus indicate the relatively weaker specificity for such a translational boundary, and suggest that the pervasive, low-rate translation of lncRNAs may possibly provide raw materials for selection to act on.

As for the mode for de novo gene origination, the continuum model proposes a series of intermediate processes^8^, while the pre-adaptation model predicts the existence of exaggerated gene-like characteristics in new genes and an all-or-nothing transition to functionality^26,27^. On the basis of the findings in this study, it is possible that both models may be at play in de novo gene birth, and that the *cis* elements directed lncRNA–mRNA transition demonstrated here could provide evidence to reconcile both models (Fig. 4i). First, the low-level, pervasive translation of sequences not under strong constraints may provide raw materials for selection to act on, which is a common basis for both the pre-adaptation and the continuum model (Fig. 4i). Second, compared with genome-wide protein-coding genes and lncRNA loci, both the de novo genes and their macaque orthologues encoding lncRNAs showed extreme features of higher GC content, suggesting the involvement of the pre-adaptation model. In this context, the precursor lncRNAs of de novo genes may represent the ‘hopeful monsters’ after the avoidance of the most toxic ‘hopeless monsters’. On the other hand, both the de novo genes and their macaque orthologues encoding lncRNAs showed intermediate level of ISOR and N/C ratio in contrast to genome-wide lncRNAs and protein-coding genes, suggesting the involvement of the continuum model. To this end, the precursor lncRNAs of de novo genes may represent ‘proto-genes’ with intermediate levels of ISOR and N/C ratio, a finding also suggests the roles of splice-directed nuclear export as a key step in de novo gene birth. Third, for neutral lncRNAs, the mutations contributing to active nuclear export, and further abundant translation, are typically removed by purifying selection, which may represent one of the key steps to remove the toxic ‘hopeless monsters’ as proposed in the pre-adaptation model (Fig. 4g,h). The process of nuclear export thus represents a coarse boundary to control for subcellular spatial separation for mRNAs and lncRNAs. With such a boundary, a de novo gene could acquire gene-like features and biological functions along with its origination, becoming the ‘successful stowaway’ actively passing through this boundary only when mutations that contribute to stable nuclear export activity maintained when the protein it encodes is adaptive (Fig. 4i).

Although these de novo genes are selectively constrained, indicating their functions in general, it is difficult to clarify their definite biological functions. In previous studies, we and others have linked de novo genes to human brain development and functions. However, these studies are largely speculative, owing to the correlation data such as the differential expression in temporospatial regulations and diseases. Theoretically, two strategies of transgenic and phenotypic study could be deployed to study the functions of human-specific genes, including the knock-out design to remove the gene from human systems, or the knock-in design to include the human-specific gene in other model animals who normally lack it. In this study, we introduced both of the knock-out strategy in human cortical organoids and the knock-in strategy in transgenic mice to clarify the functions of one hominoid-specific de novo gene. Strikingly, we found that even the manipulation of one de novo gene could significantly change the size of the organoids by modulating the speed of the neuronal maturation, recapitulating the human–macaque difference in the size and cell type composition of the foetal brains. Notably, although new genes typically interact with other coevolved genetic elements to produce a specific new phenotype^67,68^, we found that the ectopical expression of even one de novo gene in mice could induce enlarged brain and the cortical expansion, suggesting that the de novo genes could quickly obtain their functions through the establishment of new interactions with pre-existing genetic elements.

## Methods

### Identification of hominoid-specific de novo genes

We previously identified 64 hominoid-specific de novo genes on the basis of mass spectrometry data^7^. As ribosome profiling data can provide additional translation evidence for candidate new genes, we expanded this list by integrating 72 ribosome profiling datasets of human lymphoblastoid cells from GSE61742 (ref. ^69^). We also downloaded and analysed the RNA-seq data of lymphoblastoid cells from GSE19480 as the input^70^. The reads aligned to ribosomal RNAs, as defined by SILVA^71^ and Ensembl (version 75), were removed. The remaining reads were then aligned to the human genome (hg19) by TopHat2 (version 2.1.1) to obtain the read coverage for each locus. We then used Ribotaper (version 1.3.1a) to define the P-site and identify novel ORFs with default parameters. To control for false positives, only candidate ORFs >50 bp supported by five or more independent datasets were retained.

To identify additional de novo genes, the ORFs were also required to meet the criteria following our previous experience^7^: (1) the candidate protein sequences were not detected in out-group species. We used BlastP and BlastX (E-value cut-off of 1.0 × 10^−6^) to search for similar sequences in eight species (chimpanzee, orangutan, rhesus macaque, mouse, guinea pig, dog, hedgehog and armadillo) to ensure the absence of this protein in the out-groups; (2) the candidate gene did not originate through gene duplication. We performed sequence alignments against all annotated human proteins (BLAST E-value cut-off of 1.0 × 10^−6^) to remove candidates resulting from gene duplications; (3) we retained only genes with ancestral common disablers shared in multiple out-group species to ensure that the gene are newly originated in humans rather than being an old gene lost in the out-groups. The alignments of the coding regions in these de novo genes with the orthologous sequences in rhesus macaque are summarized in Supplementary Fig. 1.

### Subcellular fractionation

For HEK293T and LLCMK2 cells, RNAs and proteins from the cytoplasm and nuclei were fractioned using a PARIS kit (Life Technology, AM1921). The frozen brain tissues from macaque and human were fractioned using hypotonic buffer (HEPES 20 mM; KCl 10 mM; EDTA 1 mM; glycerol 10%; NP-40 0.2%). An RNase inhibitor (Invitrogen, RNaseOUT) was added throughout the RNA fractionation procedure, and PMSF (Sigma) and proteinase inhibitors (Roche) were added during the protein fractionation procedure. The quality of the subcellular fractionation was evaluated by western blotting assays, with lamin-B (BioWorld, MB2029) and β-tubulin (KBQBIO, RLM3139) used as the marker for the nuclear and the cytoplasmic fraction, respectively.

### Library preparation and deep sequencing

Total RNA was extracted from subcellular fractions of the cell lines and tissues, as well as the human cortical organoids, following the TRIzol RNA isolation procedure. The quality of the input RNA was evaluated using an Agilent 2100 Bioanalyzer. Total RNA samples were then subjected to strand-specific, rRNA-depleted RNA-seq or strand-specific, poly(A)-positive RNA-seq, following the previously described pipelines^72^. Deep sequencing was then performed on an Illumina XTen sequencing system.

### Calculation of RNA N/C ratio

We used the ratio of RNA abundance in the nucleus and cytosol (N/C ratio) to estimate the level of nuclear export for a specific transcript. Briefly, RNA-seq reads from cytoplasmic and nuclear fractions were aligned to the reference genome (hg19 for humans and rheMac2 for macaques) by HISAT2 (version 2.0.5) (ref. ^73^). Owing to the lack of lncRNA annotations, we first identified the list of macaque lncRNAs following a previously reported method^72^. The expression levels of the annotated genes and lncRNAs were then calculated by Stringtie (version 1.3.4d) (ref. ^74^). Weakly expressed genes ((FPKM_nucleus_ + FPKM_cytoplasm_) <0.2) were discarded^21^. The N/C ratio was then log_2_ transformed after adding a pseudocount of 0.1 to each FPKM score. For cross-species N/C ratio comparisons, the raw score of the N/C ratio was normalized using a *z*-score approach.

### Key *cis* elements for RNA nuclear export

To identify key *cis* elements regulating RNA nuclear export, we developed a deep learning classification model on the basis of a CNN with multiple convolutional and pooling layers, as well as one fully connected layer^75,76^. The information about the subcellular localization of transcripts was extracted from the subcellular fractionation data of HEK293T cells (Extended Data Fig. 2), with the transcripts showing the highest and lowest N/C ratio scores defined as the nuclear (highest 5%)- and cytosol (lowest 5%)-located sequences, respectively. The sequences were labelled according to the subcellular localization of the transcripts they encoded and were encoded as binary matrix^77^. The labelled and encoded sequences were split by 11 bp sliding windows and then input to the convolutional-pooling layers of a deep learning model with automatic learning and weight updating. We constructed the training, testing and validation sets in a proportion of 8:1:1, for nuclear- or cytoplasmic-localized RNAs with a length of <5,000 bp. The performance of the model was then evaluated with the testing set.

To identify the key *cis* elements for RNA nuclear export, we then dissected and annotated the key sequence motifs in the above model. Briefly, sequences informative in differentiating RNAs with different subcellular localizations were extracted and ranked according to their activation values in the first convolutional layer of the model. The motifs of the informative sequences were then visualized using WebLogo^78^ and were further annotated with Tomtom (version 5.0.5) (ref. ^79^) using annotation from the JASPAR database^80^ and a manual curation of motifs based on literature.

### Calculations of the U1 density and ISOR

Genome-wide U1 small nuclear ribonucleoprotein recognition sites in humans and rhesus macaques were defined with a previously reported pipeline^16,81^, in which only U1 sites defined as ‘strong’ were used in the calculations for protein-coding genes and lncRNAs. Notably, all of the U1 binding sites were used in the U1 density calculations for de novo genes and their macaque orthologues encoding lncRNAs, considering the relatively smaller datasets. We also defined ISOR, the ratio of spliced-out length and the exon length of the transcript, to quantify the average splicing level of each gene locus. To evaluate the efficiency of ISOR in quantifying splicing levels, we used the transcriptomes of one human brain sample with both RNA-seq and Iso-seq dataset published in previous report^17^, and used Iso-seq data to estimate the splicing levels of full-length transcripts directly.

### CRISPR/Cas9 editing on de novo genes

To obtain mutant HEK293T cells with mutations in de novo genes, we first identified 14 de novo genes moderately expressed in these cells (FPKM >0.5) and designed a gRNA library to cover all potential U1-binding sites and splice sites on these genes (–100 bp to +100 bp, Supplementary Table 3). A total of 1,511 gRNAs were designed by E-crisp (one to three gRNAs for each site), which were inserted into the LentiCrispr-V2 plasmid to develop an gRNA library. A cell library harbouring these gRNAs was then constructed through lentiviral delivery at a suitable multiplicity of infection (MOI), in which one or more viruses could infect one cell, while the infection did not affect the survival of the cells. After viral infection with puromycin selection, the cells were kept in culture for 20 days and then collected for subcellular fractionation. Fractional complementary DNA from the nuclei and cytoplasm was then synthesized from 1 μg of total RNA, and the cDNA sequences encoded by the 14 target de novo genes were amplified with the primers listed in Supplementary Table 4. All PCR products were then combined and purified, and the library was prepared for deep sequencing.

### CRISPR/Cas9-induced mutations and RNA nuclear export

The reads of the 14 PCR-amplified de novo genes were mapped to the human genome (hg19) using HISAT2 (version 2.0.5) with default parameters. Mutation sites on these genes were then identified with a previously published pipeline^72^. To characterize the effect of each CRISPR/Cas9-induced mutation on the nuclear export activity of the corresponding transcript, we compared the number of reads with the reference allele or mutated allele in the nuclear and cytoplasmic fractions. Reads carrying more than one mutation were discarded to avoid uncertainty in explaining the compound effects of multiple mutations. For each CRISPR/Cas9-induced mutation, with the number of reads carrying the mutated allele in the nuclear fraction, the reference allele in the nuclear fraction, the mutated allele in the cytoplasmic fraction and the reference allele in the cytoplasmic fraction, Fisher’s exact test was applied to test whether this mutation significantly increased or decreased the RNA nuclear export activity, with a corrected *P* value threshold of 0.05. The effect of each exonic mutation on U1 activity was quantified by a maximum entropy model^16,81^, and only mutations with the most dramatic changes of U1 scores (the top 50%) were included in the following analyses. Mutations that significantly changed the splice efficiency were defined by comparing the ratio of junction reads and genomic reads in the reference and mutation-containing transcripts (Fisher’s exact test, Benjamini–Hochberg corrected *P* value < 0.05, with the difference of ratios greater than 5%). These were further compared with the effect on the nuclear export activity of the corresponding transcript to clarify the relationship between U1 activity/RNA splicing and RNA nuclear export.

### Genetics analyses in human and macaque populations

To investigate whether purifying selection was involved in maintaining polymorphic sites in de novo genes, we applied population genetics analyses in a population of 652 humans^82^ and 572 macaques^72^. We calculated the pN/pS ratios for these de novo genes, the protein-coding genes conserved in human and rhesus macaque, and the orthologues of these de novo genes in macaque, in which the pseudo-nonsynonymous and pseudo-synonymous sites in macaque orthologues were determined by codon-level alignment with human de novo proteins^83^.

We then focused on the human polymorphic sites located near the splicing junctions or U1 binding sites of these de novo genes, as well as the macaque polymorphic sites located on the orthologous regions of the above human regions. For each of these sites, the derived allele was defined following the Enredo–Pecan–Ortheus pipeline with six species^84^ (human, gorilla, chimpanzee, orangutan, macaque and marmoset). The site frequency spectra of derived alleles were then estimated for different groups of sites, with 1,000 times of bootstrap to deduce the confidence intervals. For each site frequency spectrum, the level of skewness was calculated with R package (version 4.1.2), which was compared with that of the background as estimated by 10,000 times of bootstrap of the synonymous sites.

### Analyses on the segregating sites

To understand the genetic elements driving the lncRNA–mRNA transition in de novo gene origination, we identified the segregating sites on these loci that were fixed after the divergence of human and macaque, which were defined following the Enredo–Pecan–Ortheus pipeline. The effects of the segregating sites on the splice efficiency and the affinity of U1 binding were further estimated. Briefly, the effect of each segregating site on the affinity of U1 binding (U1-like score) was quantified by a maximum entropy model using FIMO (version 5.0.5) (refs. ^16,81^), and the effect of each segregating site on splice efficiency was calculated by MaxEntScan^85^. On each lineage, the proportions of segregating sites increasing or decreasing the splice efficiency or the affinity of U1 binding were then calculated.

### Genes co-expressed with de novo genes

To identify the expression profiles of de novo genes across tissues and individuals, processed RNA-seq datasets from six human tissue types (cerebellum, cortex, kidney, liver, lung and testis) were downloaded from the GTEx project^82^. The normalized gene expression levels in FPKM were calculated with StringTie (version 2.1.5) (ref. ^74^). On the basis of the brain expression data in 134 human individuals, as well as in 35 macaque animals, the within-population Pearson correlation coefficients between each de novo gene and other genes were calculated and compared to identify the co-expressed human genes for each de novo gene in humans, or the co-expressed macaque genes for the macaque orthologues of human de novo genes. Only genes with the expression of FPKM ≥0.2 were included in the calculations, and a pair of co-expressed genes was defined with a corrected *P*-value cut-off of 0.05 in the calculation of the Pearson correlation coefficient.

### Generation of human cortical organoids

The human ES cell line H9 (WA09) were purchased from WiCell and cultured feeder-free on Matrigel (Corning)-coated six-well plates with Essential 8 medium (Thermo Fisher Scientific, A1517001). Cortical organoids were generated from human pluripotent stem cell (hPSC) lines using a reported protocol with some modifications^86^. Briefly, hPSCs were dissociated into single cells with Accutase (Thermo Fisher). Then, 10,000 cells were plated in each well of a PrimeSurface 96V Plate (Sumitomo Bakelite, MS-9096VZ). Embryonic bodies (EBs) were cultured in organoid-induction medium (Supplementary Table 6) for the first 6 days. The medium was then changed on day 6 to organoid-proliferation medium (Supplementary Table 6). To promote growth and differentiation, organoids were transferred into ultralow-attachment 10 cm dishes on day 7 using a cut 1,000 μl pipette tip (Nunc) and spun with a Sunflower Mini-Shaker (Grant-Bio) with medium changes every other day (day 8 to day 25). To promote neural differentiation, organoids were subsequently cultured in organoid-maturation medium (Supplementary Table 6) starting at day 25. The medium was changed every 2 days.

RNA-seq assays were applied to samples of human cortical organoids at four timepoints (protocol days 20, 30, 50, 60 and 70), and the expression profiles of 74 de novo genes were calculated with StringTie (version 2.1.5) (ref. ^74^). The correlations of temporal expression profiles were then compared between the human cortical organoids and the four corresponding stages in early brain development^87^ (Supplementary Tables 7and 8), according to previous reports^86,88^.

### Generation of hNPCs

Differentiation of neural progenitor cells (NPCs) was based on a protocol as reported previously with some modifications^89^. Briefly, hPSCs were detached with dispase (Gibco) to form EBs for the first 4 days in neural induction medium (Supplementary Table 6). At day 4, the EBs were plated onto GFR-Matrigel (Corning)-coated plates and then cultured in neural proliferation medium (NPM; Supplementary Table 6). From day 4 to day 9, the cells were cultured in NPM supplemented with LDN-193189 and SB-431542 at same concentration; and from day 9 to day 11, the cells were cultured in NPM without supplementing any small molecules. At day 11, the cells were dissociated by Accutase and replated onto GFR-Matrigel-coated plates at a density of 2 × 10^5^ cells cm^−^^2^, and then cultured in NPM supplemented with 10 ng ml^−1^ bFGF for the next 3 days. At day 14, the NPCs were ready to conducted the following gene-editing experiments.

### Gene editing in hNPCs

We developed two sets of CRISPR/Cas9 targeting constructs to disrupt the splice sites or U1 recognition sites on *ENSG00000205704* in hNPCs (Fig. 6d). To disrupt the splice sites of *ENSG00000205704*, an single guide RNA was designed to target the first splice site of *ENSG00000205704* (gRNA-I1-3: ACGCTGTGTCTCCGCAGCCAAGG). To disrupt the U1 recognition sites on *ENSG00000205704*, a pair of single guide RNAs were designed to induce a 454 bp deletion of U1-enriched region at the second exon of this gene (gRNA-U1-KO-1: CTGGTTCGTGCCGGTCTACTTGG, gRNA-U1-KO-2: CTGCGCAGCGCAAAGGCACTGGG).

When editing the RNA splice sites and the U1 recognition sites in NPCs, we performed nucleofection with LONZA 4D-Nucleofector. A 3 μg pmax-Cas9-GFP plasmid and a 3 μg pmini-gRNA plasmids (gRNA-I1-3), or two 3 μg pmini-gRNA plasmids (gRNA-U1-KO-1 and gRNA-U1-KO-2), were used for a single nucleofection event and nucleofected by program CU-133. After 40 h, GFP-expressing Cas9-transduced cells were selected by FACS (BD Biosciences, FACSAria Fusion) and replanted. The knock-out of U1-enriched region was genotyped with PCR assays (primers: U1-KO-GT-F: TGCTTGGGCCTCGGGCTCTG, U1-KO-GT-2: GCCGGTGCCATTGAGTGGAGG), while the retention level of intron was conducted by real-time PCR assays, with primers across the splicing sites (primers: Intron-retention-F: GCTGATTGGCTGAGACAGGT, Intron-retention-R: CCCTACCTCCCAAGCCATTG).

For the cultured cells with or without the gene editing, the total RNAs from the cytoplasm and nuclei were then fractioned as described above, and the expressions of *ENSG00000205704* in the cytoplasmic fraction and the nuclear fraction were quantified by real-time PCR assays, with a pair of primers targeting the ORF of *ENSG00000205704* (ORF-RT-F: CGGAGCCCTCATTTCTTCGT, ORF-RT-R: TGGCTTCGACCTGCCTAAAG). The levels of the nuclear export of *ENSG00000205704* in different samples were then estimated on the basis of the RNA expression levels in the nucleus and cytosol.

### hESC lines with *ENSG00000205704* knock-out

A pair of gRNAs were designed to remove 40 bp of the ORF of *ENSG00000205704*, inducing a frameshift mutation (Fig. 6d; gRNA-KO-1: CCGGCCCTGCAACTTTAGGCAGG, gRNA-KO-2: AAGAACCTAAGAACCTCGTTTGG). We then performed nucleofection with LONZA 4D-Nucleofector as described above. A 1.5 μg pmax-Cas9-GFP plasmid and two 1.5 μg pmini-gRNAs (hESC-KO-1 and hESC-KO-2) were used for a single nucleofection event and nucleofected by program CB-150. Individual colonies were then picked and genotyped with primers (KO-GT-F: ATGTCTATGGCTGCCTGTCCTG, KO-GT-R: TCATCACCCCATGCTTGCCC) to select positive colonies.

### hESC lines with *ENSG00000205704* over-expression

The ORF sequence of *ENSG00000205704* and the human codon-optimized HA × 3 sequence were cloned into pLVX-TRE3G-IRES (Clontech, 631362), which was subjected to the production of the lentivirus stock. For transduction, concentrated viruses were added into the hPSC medium (MOI 10) and incubated with polybrene (Sigma, 8 μg ml^−1^) and Y-27632 (10 ng ml^−1^) for 24 h. The virus packaged with pLVX-EF1a-Tet3G constructs (Clontech, 631359) were also co-tranducted into hESCs to built the Tet-on system. After selection with G418 (Sigma, 400 μg ml^−1^) and puromicin (Sigma, 0.5 μg ml^−1^) for 2 weeks, individual colonies were picked and further genotyped with real-time PCR assays under 2 days of doxorubicin (Sigma, 1 μg ml^−1^) treatment (primers: ORF-RT-F and ORF-RT-R).

### Construction of *ENSG00000205704* knock-in mice

Mouse lines were on a C57BL/6 background. The R26-e (CAG-LSL- ENSG00000205704-3xHA-WPRE-polyA) mice and Dppa3-IRES-Cre mice were constructed by Shanghai Model Center. Briefly, a DNA fragment of CAG-LSL-ENSG00000205704-3xHA-WPRE-polyA was inserted into Rosa26 locus in mouse embryo by CRISPR/Cas9 editing. To induce the expression of ENSG00000205704 in mice, the transgenic mice were generated by hybridization of homozygous R26-e (CAG-LSL-ENSG00000205704-3xHA-WPRE-polyA) mice and heterozygous Dapp3-IRES-Cre mice, and genotyped by PCR assays (primer: TG-mice-1: TGGGTTGGGTGTCTGTTTCATTGT, TG-mice-2: GATCCACCTGTCTCTGCCTTCC, and TG-mice-3: GACCTTGCATTCCTTTGGCGAGAG).

### Immunofluorescence staining

Samples of organoids and mice brains were subjected to a standard pipeline for immunofluorescence staining, with the following primary antibodies: anti-PAX6 (Thermo Fisher, 42-6600, 1:400), anti-CTIP2 (Abcam, ab18465, 1:200), anti-SOX2 (R&D, AF2018, 1:400), anti-NEUN (Abcam; ab177484, 1:200), anti-TBR1 (Abcam, ab31940, 1:200), anti-CTIP2 (Abcam, ab18465, 1:200) and anti-SATB2 (Abcam, ab92446, 1:200). Images were taken under a confocal microscope (Carl Zeiss LSM880) and processed using ZEN 2012 (version 1.1.0.0). Cells were manually counted using ImageJ.

### Reporting summary

Further information on research design is available in the Nature Research Reporting Summary linked to this article.

### Supplementary information

Supplementary Information Supplementary Fig. 1.

Reporting Summary

Supplementary Table 1 Supplementary Tables 1–9.

### Source data

Source Data Fig. 1 Unprocessed western blots for Fig. 1.

Source Data Fig. 2 Unprocessed western blots for Extended Data Fig. 2.

Source Data Fig. 3 Unprocessed western blots for Extended Data Fig. 7.

## Data Availability

High-throughput sequencing data from this study have been submitted to the NCBI Sequence Read Archive (SRA) (https://www.ncbi.nlm.nih.gov/sra/) under accession number PRJNA750575. For details, please see Supplementary Table 9. Source data are provided with this paper.

## Code availability

The codes in this study can be found at GitHub via URL: https://github.com/ZhangJiePKU/DenovoProject
