# Supplementary material for: De novo genes with an lncRNA origin encode unique human brain developmental functionality
Source: Nat Ecol Evol. 2023 Jan 2;7(2):264–78. doi: 10.1038/s41559-022-01925-6 (PMC9911349; doi:10.1038/s41559-022-01925-6)
Supplement: Supplementary file 1 — Supplementary Fig. 1. [file 41559_2022_1925_MOESM1_ESM.pdf]

nature ecology & evolution

Article

https://doi.org/10.1038/s41559-022-01925-6

De novo genes with an lncRNA origin encode unique human brain developmental functionality

In the format provided by the authors and unedited

Supplementary Figure 1. The alignments of the CDS regions in *de novo* genes with the orthologous regions in rhesus macaque. The disruptive mutations or indels in rhesus macaque were highlighted with red boxes.

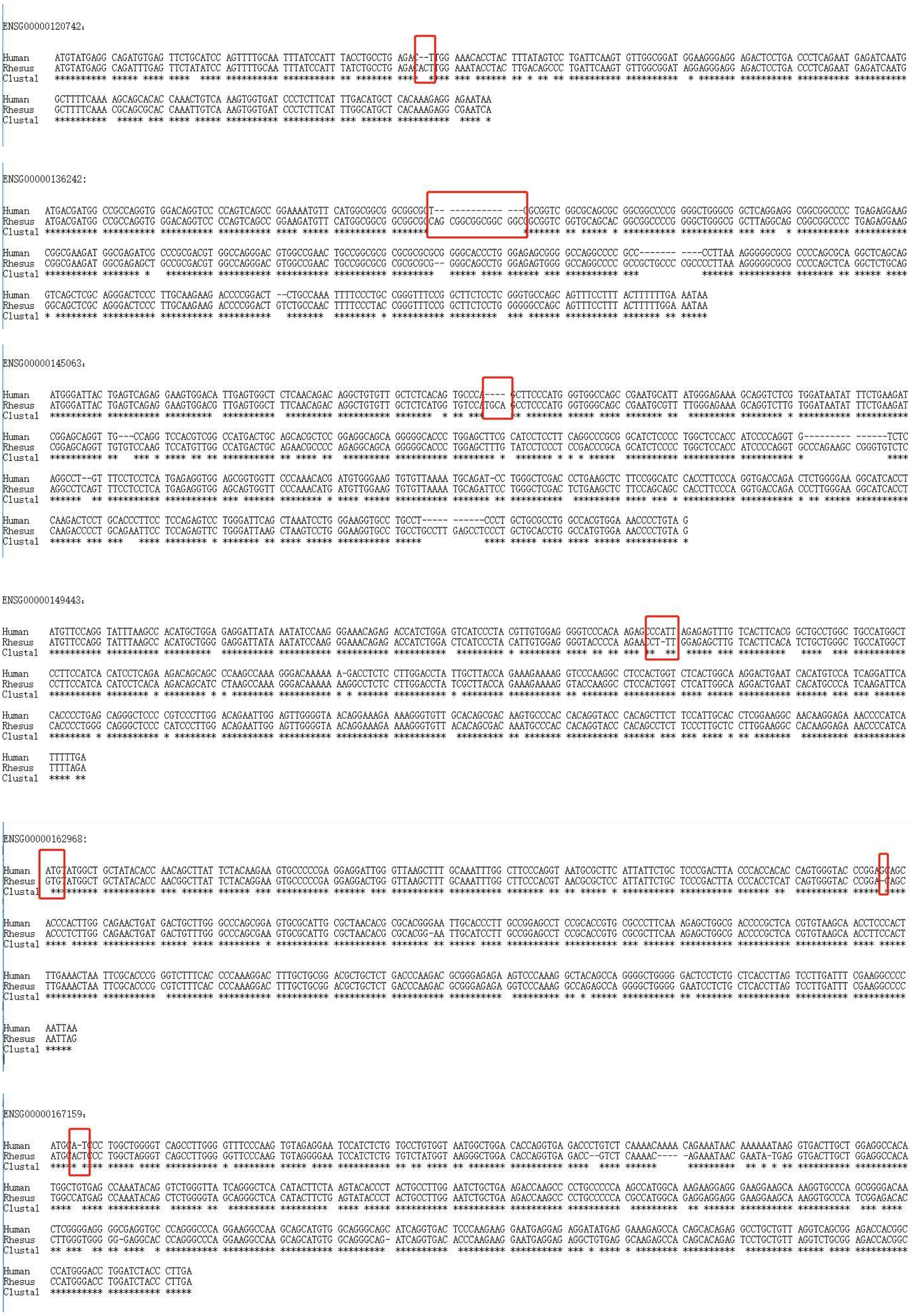

ENSG00000167747:

```
Human   ATGACAGTGC TGGAGGCCGT CTTGGAGATC CAGGCCATCA CTGGCAGCAG GCTGCTCTCC ATGGTGCCAG GGCCCGCCAG GCCACCAGGC TCATGCTGGG ACCCAACCCA GTGCACAAGG ACTTGGCTGC TGAGCCACAC ACCCAGGAGA
Rhesus  ATGACGTTGT TCTAGGCCGT CCTGGACATC CAGGCCATTG CCGACAGCAG GCTGCTTTCC ATGGAGCCAG --------AG GCTGCCAGGC TCACGCTAGG ACCCAACCCA GTGCACAAGG ACCTGGCTGC TGAGCCGCAC ACCCTGGAGA
Clustal *****  **  *  ******* * **** *** ********    * * ****** ****** *** **** *****         ** **  ****** *** *** ** ********** ********** ** ******* ****** *** **** *****

Human   AGGTGGATAA GTGGGCTACC AAGGGCTTCC TGCAGGCTAG GGGAGGAGCC ACCCCCGCTT CCCTATTGTG ACCAGGCCTA TGGGGAGGAG CTGTCCATAC GCCACCGTGA GACCTGGGCC TGGCTCTCAA GGACAGACAC CGCCTGGCCT
Rhesus  AGGTGGATGA GTGAGCTACC AAGGGCTTCC TGCAGGCTAG GGGAGGAGCC AGCCT-GCTC CCCTGTTCTG ACCAGGCCTG CGGGGAGGAG CTGCCCATAT GCCACCATGA GACCTGGGCC TGGCTCCCGA GGACAGATAC CGCCTGGTCT
Clustal ******** * *** ****** ********** ********** ********** * **   ***   **** ** ** *********   ********* *** *****   ****** *** ********** ****** * * ******* ** ******* **

Human   GGTGCTCCAG GGGTGAAGCA GGCCAGAATC CTGGGGGAGC TGCTCCTGGT TTGA
Rhesus  GGTGCTCCAG GGGTGAAGCA GGCCAGAATC CTGGGGGAGC TGCTCCTGGT TGCC
Clustal ********** ********** ********** ********** ********** *
```

ENSG00000172927:

```
Human   ATGCCCTCA GAATCTGCGT CACATACACC CCAGCTCTCC CGATAGGTCT CTGCACTCGC TGTTGCCTCT GCCTGGAACA GTCTCCCTCC TGGTGTCATT GTCTCCGTGG TGTGTCCTTC CTGACCTTCC ACCTCCACCA GTCTGTCCCC
Rhesus  GTGCCCTCA GGGTCCGCGT CACACACACC CCAGCTCTCG TGACGGGTCT CTGCACTCGC TGTTGCCTCC GCCTGGAACA GTCTTCCTCC TGGTGTCATC ATCTCCGTGG TGCGTCCTTC CTGACCTTCC ACCTCCACCA GTCTGACCCC
Clustal  ********* *  ** **** **** ***** *********    **   ***** ********** *********  ********** **** ***** *********   ********* ** ******* ********** ********** ***** ****

Human   CTTGGGGACA GGGACTCGTT GCTCATGTTC ACCCGGCAGG CTGGACACTT CGTGGAGGGC TCCAAAGCCG GCAGATCCCG GGGCCGCCTC TGTCTCTCCC AGGCCCTGCG TGTTGCGGTG AGAGGAGCAT TTGTGTCTCT GTGGTTTGCT
Rhesus  ACCGGGGACA GGGACTGGTC ACTCATGTTC ATCCGGCAGA CCAGACACTT CGTGGAGGGC TCCAAAGCTG GCACATCCCG GGGCCTCCTC TCTCTCTCCC AGGCCCTGTG TGTTGCGGTG AGAGGAGCAT TTGTGTCTCT GTGGTTTGCT
Clustal ******* ****** **   ********* * *******   *   ******* ********** ******** * *** ****** ***** **** * ******** ******** * ********** ********** ********** **********

Human   GCTGGAGCTG GTGACCGGGA GAGAAACAAG GGAGACAAGG GTGCCCAGAC AGGTGCGGGG CTCAGCCAGG AGGCAGAAGA CGTGGACGTG TCCCGGGCCA GGAGGGTCAC AGATGCACCA CAAGGCACTC TGTGTGGCAC TGGGAACAGG
Rhesus  GCTGGAGCTA ATGACCTGGA GAGAAACAAG GGAGACAAGG GTGTCCAGAC AGGTGCGGGG CTCAGCCAGG AGGCAGAAGA CGTGGATGTG TCCCAGGCCA G--------- -----CGCCA CCAGGCACTC TGTGTGGCAC TGGGAACAGG
Clustal *********    ***** *** ********** ********** *** ****** ********** ********** ********** ****** *** **** ***** *                * *** * ******** ********** **********

Human   AATTCTGGGA GTCAGTCTGC AAGGGTGGTG GGCGTTGCTC ACCTGGGAGA AGCCTTTAGA GTGGGCGTTG AGCAGGCCAT TAGCTCGTGC CCTGAGGAGG TGCATGGGCG GCATGGGCTC TCCATGGAAA TTATGTGGGC GCGAATGGAT
Rhesus  AATTCTGAGA GTTAGTCTGT AAGGGTGGCG GGCATTGCTC CCCTGGGAGA AGCCTTTCCA GTGGGCGCTG AGCAGGCCAT TAGCTCGTGT CCTGAGGAGG TGCATGGGTG GCCTGGGGTC TCCACGGAAA TTATGTGGGC GCGCATGGGT
Clustal ******* ** ** ******   ******** * *** ******  ********* *******   * ******* ** ********** *********  ********** ******** * ** **** ** **** ***** ********** *** **** *

Human   GTGGCTCTGC GCTCACCTGG GCGAGGACTT CTGGCCGGTG CCGGGGCACT CTGCATGACC CTGGCAGAAT CGAGCTGCCC TGACTATGAA AGGGGAAGAA GAGCATGCCT GACCCTCCAC CGGCACCCCA CCCCTCACTG CTCCACCTGG
Rhesus  GTGGCTCTGT GTTCACGTGG GCGAGGACTT CTGACCGGTG CCAGGGCACT CTGCATGACC CTGGCAGAAT CAAGCTCTCC CGACTGTGGA AGGGGAAGAA GAACATGCGT GACCCCCCAC CAGCACCGCA CCCCTCACTG CTCCACCTGG
Clustal *********  * **** *** ********** *** ****** ** ******* ********** ********** * ****   **   **** ** * ********** ** ***** * ***** **** * ***** ** ********** **********

Human   GGCCTGCCTC TGCGGGTGGC TGGGTCCTGG CTGACTGTTG TGACTGTTGA GGCCCTGGG- GGGGTGGCGC ATGGGAGTTA GGAGGACTGG CCAGGTGGGG CCCACTATGC ACCCACCCCC AGTGTCAGGT GCTTCTCCTC TCCTCCTCCA
Rhesus  GGC-TGCCTC TGGGGGTGGC TGGGTCCTGG CTGACTGTTG TGACAGTTGA GGCCAGGGGT GGGGTGGGGC ATGGGAGTTA AGAGGACTGG CCAGGTGGGG CACACCGTGT GCCCAGCCCC AGTGTCAGGA ACTTCCC--- -CCTCCTCCT
Clustal *** ****** ** ******* ********** ********** **** ***** ****   ***   ******* ** **********  ********* ********** * ***   **    **** **** *********    **** *      ********

Human   CCACCTCCTC CTCCTCCTCC TCATCATCAT CCTCACTTGT TGA
Rhesus  CCACCCCCTC GTCCTCCTCC TCCTCATCAT CCTCACTTGT TGA
Clustal ***** ****  ********* ** ******* ********** ***
```

ENSG00000174407:

```
Human   ATGGATACGG GGTCCCGGGA GGCAGCCTTC ACCAGGGACC TGCCCAGGAA ACCAGCAGAT GCCCAGGTCA GCCCTGAAAT GTCCATTACT CACAAAGAAA AGGAAAATGC TCATTTGAAA GA-AATTCTT CTCTTTGTCA ATGCTGAGGC
Rhesus  AGGGACACAG CATCCCGGGA GGCAGCCTTC ACCAGGGACC TGCCCAGGAA ACCGCCAGCT GCCCAGGTCA GCCCTGAAAT GTCCATTACT CACAAAGAAA AGGAAAATGC TCATTTGAAA GACAATTCTT CTCTTTGACA ATGCTGAGAC
Clustal * *** ** *    ******** ********** ********** ********** ***   *** * ********** ********** ********** ********** ********** ********** ** ******* ******* ** ********* *

Human   ATTCTCTCAG CCCCAGCCCC ACAGTGCTCC AGTGTGTGAA GGGCAACAGC TGACGGGGAA GTTCAGCACA TCTGTGCTGA CCAGGGCTGG AGGCGATGCC AGTCCGTGCA GCTGGGAACG CCTCCTCTGC TATGGCTGGA GCCACTGCTG
Rhesus  ATTCTCCCAG CCCCAGCCCC ACAGTGCCCC AGTGTGTGAA GGACAACAGC TGTTGGGGAA GTTCAGTGCA TCTGTCCTAA CCAGGGCTGG AGGTGGTGCC AGTCTGTGCA GCTGGGAACA CCTCCTCTGC TGTGGCTGAA GGGGCTCCAC
Clustal ****** *** ********** ******* ** ********** ** ******* **  ****** ******  ** ***** ** * ********** *** * **** **** ***** *********  ********** * ****** * *   ** *

Human   A
Rhesus  C
Clustal
```

ENSG00000175913:

```
Human   ATGCAGCCTT CCTGGACTCC CGCACCGGTA CAGCGCACTG CATGTAATAT TACAGCCTGG GGCGGCGAAT TTGGGAAGGA GGGAGAGGGT CGTTGTGAGC AGGTTGCACT CAGCTCTGGC CCTCCGGAGG GTGCACTTCA CGCCTCCAGA
Rhesus  ACGCAGCCTT CCTGGACTCC CGCACCGGTA CAGCGCGCTG CATGTAATAT TACAGCCTGG GGCGGCGAAT TTGGGAAGGA GGGAGAGGGT CGTGGCGAGC AGGTTGCACT CAGCTCTGGC CCTCCGGAGG GTGCACTGCA CGCCTCCAGA
Clustal * ******** ********** ********** ****** *** ********** ********** ********** ********** ********** *** * **** ********** ********** ********** ******* ** **********

Human   GAAGGTCCAC AGCCTCCTGG TGCTGAAAAC CTACGTCCCT CCACTGGCGA AACATTTGTG CAGAGCGGAC GATGGGATGG GGGCTGGAGA GGAGCAATGA AAGGGAGGAG ACACAGGCAA GCCTCAACTC CCCCAACTCG TCCCGAATCG
Rhesus  GAAGGTCCAC AGCCTCCTGG TGCTGAAAAC CTACGTCCCT CCACTGGCGA AACATTTGTG CAGAGCGGAC GATGGGATGG GGGCTGAGGG GGAGCAGTGA AAGGGAGGAG ACACAGGCAA GCCCCAACTC CCCCAACTCG TCCCGAATCG
Clustal ********** ********** ********** ********** ********** ********** ********** ********** ******   *  ****** *** ********** ********** *** ****** ********** **********

Human   ATCTTCGTTC CAACCGCGCA GGATGGGGCC CAGATGGTTT GCAAAGCGCA CACTCGCACA ACGCAATACA CAGAACAAGA TTCCGTTGTT ACAGCGCGTG GTTTACTAGA TGCTAAAAGG GTGGGAGTAG CAGGAGGGTC TTAA
Rhesus  ATCTTCGTTC CAACCGCGCA GGATGGGGCC CAGATGGTTT GCAAAGCGCA CACTCACACA ACGCA-TACA CAGAACAAGA TTCCGTTGTT ACAGCGCGTG GTTTACTAGA TGCTAAAAGG GTGGGAGTAG CAGGAGGGTC TTAA
Clustal ********** ********** ********** ********** ********** ***** **** ***** **** ********** ********** ********** ********** ********** ********** ********** ****
```

ENSG00000176833:

```
Human   ATGCAAATGA CACAAAGATC CCATCACTTT CTAGTTTGGG GAGGGGGCGG GCGCAAGGGG AAGGAAGGAG GGAAGAAAGA CATCTTCCAA CTGTTATTAT CTAGTCTCTG CAGGCTTAGA TTGGTGCTCT GCCTACATTG GAGGTGTTCT
Rhesus  ATGCAAATGA CACAAAGATC CCATCACTTT CTAGTTTGAG GAGGGGGCA- ---CAAGGGG AAGGAAGGAG GGAAAGAAGA CATCTTCCAG CTGTTCTTGT CTAGTCTCTG CAGGCTTAGA TTGGTGCTCT GCCTATATTG CAGGTGTTCT
Clustal ********** ********** ********** ******** * ********      ******* ********** ****   **** *********   ***** ** * ********** ********** ********** ***** ****   *********

Human   GCAAACCGGA GTCTTCCTCG CCAGAGTCCC ATAAACGTGC CTCAGTCAGA TGCTGGAGAG ACAGAGAAGG GAAGAGTCCA TCAGAGCCTC CTGATTCGAG CATCACACAG CAACCCTTCT GAGCTCGGTG GTCCACACAG CGCAGGGTTC
Rhesus  GAAAACCGGA GTCCTTCTCA CTAGAGTCCC AAAAACATGC CTCAGTCAGA CGCCGGAGAG ACAGAGAAAG GAAGAGTCCA TCAGAGCCTC CTGATTCTAG CGTCACACAG CAACCCTTCT GAGCTCGGTG GTCCACTCAG CACGGGGTTC
Clustal * ******** *** * ***   * ******** * **** *** **********   ** ****** ******** * ********** ********** ******* ** * ******** ********** ********** ****** *** * * ******

Human   TATGAACTCC TGAGGAGAGC CAGGAGTGCT GCTAAGCTTT CCCAGGTATG CGTGACCATG TGTGCCTTCA TCTGTGTGTG A
Rhesus  TGTGAACTTC TGCAGAGAGC AAGGAGTGCT GCTAAGCTTC CCCAGGTGTG CGTGACCATG TGTGCCTTCA TCTGTGTGTG A
Clustal * ****** * **  ******  ********* *********  ******* ** ********** ********** ********** *
```

ENSG00000176911:

```
Human   ATGACGCCCC TTCCTTATCG TTCTAGAGTC AAGCCTAGAT CCCCTACAAA TACCCATGCA CACTTAGAGC TCACTCTGGT TAAATGTTCC AAGAAGGATG TTGGATGGTC ACTTGGTATC ATCCCATGGA GTTCAGGATA CCCCCGCCCT
Rhesus  ATGACGACCC TTCCTTAT-- -TCTAGAGTC AAGCCTGGAT CCCCTACAAA TACCCACGCA CACTTAGAGC TCACTCTGGT TAAATGTTCC AAGAAGGACG TTGGATGATC ACTTGGTATC ATCCCATGGA GGTGAGGATA TGCCCGCCCT
Clustal ****** *** ********    ********* ****** *** ********** ****** *** ********** ********** ********** ******** * ******* ** ********** ********** * * ******    *********

Human   CTGAGCAGCC ATTCCTCTCC CGAGCACATT CCCCTAGAGA AGCTCTGCAC GTGTGCACAG GCACCAGCTG CAGGAGTGCC ATCCACAGCA CAGCTGTTTA TGAAAAACCG TAATCAACCC AAACATCTGT ATGAAGAAAA ACGAAGAAAT
Rhesus  CTGAGCAGCC ATTCCTCTCC CGAGCACATT CCCCTAGAGA AGCTCTGCAC GTGTGCACAG GCGCCAGCTG CAGGAGTGCC ATCCACAGCA CAGCTGTTTA TGAAAAACCA TGATCAACTC AAACG--TGT ATGGAGAAAA ATGAAGAAAC
Clustal ********** ********** ********** ********** ********** ********** ** ******* ********** ********** ********** *********  * ****** * ****    *** *** ****** * *******

Human   GCAGAGTGGC ATGTTCACAT GATGGAATAC TATGCAGCTG AGAACATGAA CAATTGGAGC CAT------- GGCATGAATG AAGCTGAGAG ATTTGAAGCG TCAACCAAGT AA
rhesus  GCATAGTGGC ATGTTCACAT GATGAAATAC TATGCAGCTG AGAACTTGAA CAATTGGAGC CATTAGCTAT AGCATGAATG AAGCTGAGAG -TTTGAAGGG TCAACCAAGT AA
Clustal *** ****** ********** **** ***** ********** ***** **** ********** ***         ********* **********  ******* * ********** **
```

```
ENSG00000177822:

Human   ATGGGCGACC TGAGCCGAAC CGCTCTCTGG CTGGGTGGTC GGCGGGATCC TTCAGGCTCC TTTCGGGTCC GGGACGGGGC GGCGGCAGAG GTGGGGCGTG CGTGCTTGGG GCTGCCGAGT GAGTGCGGCT CCCTGGTGCG CGCCCGAGCA
Rhesus  ATGGGCGACC TGAGCAGAAC CGCTCTCTGG CTCGGCGGTC CGTGGGATCC TTCAGGCTCC TTTCGGGTCC GGGACGGGAT GGTGGCAGAG GTGGGGCATG CGCGCTTGGG ACTGCCGAGT GCGTGCGACT CCCCCGTGGG CGCCCGAGCA
Clustal ********** ***** **** ********** ** ** ****  * ******* ********** ********** ********   ** ******* ******* ** ** *******  ********* * ***** ** ***  *** * **********

Human   TCTCCCCGCC CTCTCCTGGG ACCAGCAGTC TGCCCGTGGA GGGCTAGGAG CCCGCGACCA GGCCTGCCCC GGCCGTCGCC AACCTGTCCT TGGAAGCGTG GCGGAGACTG GTGCGCTCTC AGCCTCGTCG CGCCAGCCGC CTCGCGCCAC
Rhesus  TCTCCCCGCC TTCCCCTGGG ACCTGCAGTC CGCCGGTGGA GGGCTGAGAC CCGGCGACCA GGCCTGCCCC GGCCGCCGCC GACCTGTCCT TGGAAGCGTG GCGGAGACTG GTGCGCTCCC AGCCTC---- ---------- CTCGCGCCAC
Clustal **********  ** ****** *** ******  *** ***** *****  **   ** ******* ********** ***** ****  ********* ********** ********** ******** * ******                ********** 

Human   CCTCCACTTT CAGCTTCAGA GCAGAGGATT CCGCCGACCT CCACCTGTAC CTGTACTCGG CCGTTAGGAA ATCCACTTGC ATTTTTAACT ACCGTGCAGA ACCAGAAACA GATCGAGCAC TGGGAGAACA AGGACGACGC CTCCTAG
Rhesus  CGTCCACTTT CAGCTTCAGA GCGGAGGATT CCGCCG---- --ACCTGTAT CTGTACTTGG CCGTTAGGAA GTCCACTTGC ATTTTTAACT GCCGTGCAGA ACCAGAAACA GATCGAGCAC TGGGAGAACA CGGACGACGC CTCCTAG
Clustal * ******** ********** ** ******* ******       *******  ******* ** **********  ********* **********  ********* ********** ********** **********  ********* *******

ENSG00000178803:

Human   ATGGAGCAGG ACTGGCAACC TGGAGAGGAA GTCACTCCTG GTCCTGAGCC CTGTTCAAAG GGCCAGGCTC CCCTCTACCC CATTGTCCAT GTGACAGAGC TCAAACACAC AGACCCCAAC TTTCCCTCCA ACTCCAATGC TGTCGGCACC
Rhesus  ACGGAGCAGG ATTGGCAACC TGGAGAGAAA GTCAGTCCTG GTCCTGAGCC CTGTTCAAAG GGTCAGGCTC CCCTCTACCC CACTGTCCAT GTGACGGAGC TCAAACA--- -GACCCCAAC TTTCCCTCCA ACTCCAATGC TGTCAGCACC
Clustal * ******** * ******** ******* ** **** ***** ********** ********** ** ******* ********** ** ******* ***** **** *******     ********* ********** ********** **** *****

Human   TCAAGTGGCT GGAACAGGAT TGGCACGGGC TGCAGCCATA CCTGGGACTG GAGGTTCTCC TGCACCCAGC AGGCCCTTTT GCCCCTACTA GGAGCCTGGG AATGGAGCAT TGACACAGAA GCAGGAGGAG GAAGGAGAGA GCAGAG-CCA
Rhesus  TCAAGTGGCT GGAACAGGAT TGGCACAGGC TGCAGCCATA CCTGGGACTG GAGGTTCTCC TGCACCCAAC AGGCCCTTTT GCGTCTACTA GGAGGCTTGG AATGGAGCAT TGACACAGAA GCAGGAGGAG GAAGGAGAGA GCAGAGACCA
Clustal ********** ********** ****** *** ********** ********** ********** ******** * ********** **  ****** **** ** ** ********** ********** ********** ********** ****** ***

Human   GAAACCCTGC AGCAACGGAG GGCCTGCAGC AGCTGGAGAG GGCCGAGTCC TCCCAAGCCC CTGCTTTCCA TGGAGCACTT GCCAGGCAGC CATTCACAAA GTGTGTCGTT GGCAGGGATG CACCAGACCA GCTCTCCTGG CACCATCCCT
Rhesus  GAGACCCTGC AAAAATGGAG GGCCTGCAGC AGCTGGAGAG GGCTGAGTCC TCCCAAGCCC CTGCTTTCCA TGGGGCACTT GCCAGGCAGC CACTCACAAA GCATGTCGCT GGCAGGGATG CGCCAGACCA GGTCTCCTGG CACCATCTCT
Clustal ** ******* *  ** **** ********** ********** *** ****** ********** ********** *** ****** ********** ** ******* *  ***** * ********** * ******** * ******** ******* **

Human   GGCCACACTC AAGGAACACA GTTATCCCTG A
Rhesus  GGCCACACTC AAGGAACACA GTTATCCCTG A
Clustal ********** ********** ********** *

ENSG00000179522:

Human   ATGGATGTGG GCTTCCAGGG AAGGTGCTCG CGCTGGTCCC GAGCCCTCCG GGGAAGATAT TCGAGCGCGG AGCGTAAGCG CAGGGCACGC CAGCCCCGGG AGCCGCGGGA GCAGGCGCCG CGCGTCTCTG CACCACCGGG CCGCCTCCCA
Rhesus  ATGGATGTGG GCTTCCAGGG AAGGTGCTCG CGCTGGTTCC GAGCCCTCCG GGGAAGATAT TCGAGCGCGG AGCGTAAGCG CAGGGCACGC CAGCCC-GGG GGCCGCGGAA GCAGGCGCTG CGCGTCTCTG CACCACCGGG CCGCCTCCCA
Clustal ********** ********** ********** ******* ** ********** ********** ********** ********** ********** ****** ***  ******* * ******** * ********** ********** **********

Human   GCCTTCTTTC CCCAGTTTGC CCTCCTGCCG CAGTCCGGGC CGAGATTAAT TCTCTGCACT TGTGAGTGGG CACACACA-- AGTTCTCCGG GCACGATCCT TTCATCTATT TCCCTGGGGG AGTCCACCTT TTTAACGATT AACCTCCTAG
Rhesus  GCCCTCTTTC CCCAGTTTGC CCTGCTGCCG CAGTCCGGGC CCAGATTAAT TATCTGCACT TGTGAGTGGG CACACACACA AGTTCTCCGG ACACGATCCT TTCATCTGTT TCCCCGGCGG AGTCCACCTT TTTAAAGATT AACCTCCCAG
Clustal *** ****** ********** *** ****** ********** * ******** * ******** ********** ********   **********  ********* ******* ** **** ** ** ********** ***** **** ******* **

Human   CTACCGCGGG CAAGGTGGCA GGATGCGAGT GGGGCGGGGA GGGGCGTTTC ACACGTTCAG AGGCACCAAA ATTAGCTGCC AGTGCTAAAA GGCTTTGCTT TCTTCGGTTT TTGACAAATA AATGGGGTGG GATGCTTGCT TGGCCGCCCG
Rhesus  CTACCGCGGG CAAGGGGGCA GGATGCGAGT GAGGCGGGGA GGGGCGTTTC ACACGTTCAG AGGCACCAAA ATTAGCTGCC AGTGCTAAAA GGCTTTGTTT TCTTCGGTTT TTGACAAACA AATGAGGTGG GATGCTTGCT TGGCCGCCCG
Clustal ********** ***** **** ********** * ******** ********** ********** ********** ********** ********** ******* ** ********** ******** * **** ***** ********** **********

Human   CTGCCCCAGC CCGAGCCCTG GGCTCACTTA GCAGCCTGAT GCCGAGTTTC AGACGCAGTC CTGCTGCGCT TACACCCGGG CTTCTTCGCC CCCTTGCCAA AGTCTGCAGC CCGATGGATG CTGGGCGCGG GCTTTCCCTG AGCGCTTTAA
Rhesus  CTGCCCCAGC CCGAGCCCTT GGCTCACTTA GCAGCCTGAT GCCGACTTTC AGACGCAGTC CTGTTCTGCT TACACCAGGG CTTCTTCGCC CCCTTGCCGA AGTCTGCAGC CCGGTGGATG CTGGGCGCGG GCTTTCCCTG GGCGCTTTAA
Clustal ********** *********  ********** ********** ***** **** ********** *** *  *** ****** *** ********** ******** * ********** *** ****** ********** **********  *********

Human   CGCAGCTTAG GCTAAAGCCC CAGAGCTCCC ACCTTCT-AC CTCCTGTTTA TCCGCCCGCC CCTCTACCAC CGCCAAAGGA CGTGCCCCTT CAGTAG
Rhesus  CGCGGCTTAG GCTAAAGCCC CAGAGCTCCC ACCTTCTCAC CTCCTGTTTA TCGGCCCGCC CCTCTACCAC AGCCAAAGGA CGTGCCCCTT CAGTAG
Clustal *** ****** ********** ********** ******* ** ********** ** ******* **********  ********* ********** ******

ENSG00000182457:

Human   ATGGACTCCA GATTTCCAGA TTTTCAAGAC CTGGACCTGG AACCCGAAAG AGCTTGTCAC GATGCGGCAG GAACACTGGA GGTAGAT-TT TTTTTTATTT TTGAATTTTG GGACTGTTGA CCTTGCTGTG AGAAAAGAGA CAACGACTGA
Rhesus  GTGGACTCCA GATTTCCAGA TTTTCAAGAA CTGGACTTGG AACCCGAAAG AGCTTATCGG GATGTGGCAG GAACACTGGA GGTAGATATT TTTTTTATTT TTGAATTTTG GGACTGTTGA CCTTGCTGTG AGAAAAGAGA CAACGACTGA
Clustal  ********* ********** *********  ****** *** ********** ***** **   **** ***** ********** ******* ** ********** ********** ********** ********** ********** **********

Human   GCAAGCACTA CCACCAGCAC TGTTACTGGG AATTAGAAGA CCTGAGTTTC TGTCCAGACC CTCAGTGCAA ACTGAGGATG CTCCATCCAA AGTGAATTAT GTCCTGTGCC TCCTGATTGC TGAGTGTTCA CCTGGACCTT CTGACTACCT
Rhesus  GCAAGCACTA CCACCAGCAC TGTTACTGGG AATTAGAATA CCTGAGTTTC TGTCCAGACC CTCAGTGGAA ACTGAGGACA CTCCATCCAA AGTGAATTAT GTCCTGTGCC TCCTGATTGC TGAGTGTTCA CCTGGACCTT CTGACTACCT
Clustal ********** ********** ********** ******** * ********** ********** ******* ** ********   ********** ********** ********** ********** ********** ********** **********

Human   TCCCTGTGCT ATTCCATCAG CCTACAGACC TGGTACCTGG ATTTTTGCCC GAGATGATTC CTACCACCTT ACTACTGACG AAGACACCCA TTCCAGTGGA CCACTGTGA
Rhesus  TCCCTGTGCT ATTCCATCAG CCTACAGACC TGGTACCTGG ATTTTTGCCC AAGATGATTC CTACCACCTT ACTACTGAAG AAGACACCCA TTCCAGTGGA CCACTGTGA
Clustal ********** ********** ********** ********** **********  ********* ********** ******** * ********** ********** *********

ENSG00000183250:

Human   ATGGGGTGGG ACTGCAGAAG GACCACAGTA GAGAATCCCT CACCAATAAG GAACTGTGTT AATCAGGAGT GGCCAGAAGG CTCCTCTCCA GGCCTCACAG AGGGAAACAC TGGTCTAGTG AGAGACCTGC GTCCTGCCCA TCAGGACCGA
Rhesus  ATGGGGTGGC GCTGCGGAAG GACCACTGTA GAGAATCCCT CACCAGTAAG GAACTGTGTT AATCAGGAGT GGCCAGAAGC CTCCTCTCCA GGCCTCAT-G GGGGAAACAC TGGTCTCATG AGAGACCTGG GTCCTGCGCA TCAGGACCAA
Clustal *********   **** **** ****** *** ********** ***** **** ********** ********** *********  ********** *******  *  ********* ******  ** *********  ******* ** ******** *

Human   AGCGGCACGC GTGAGGATCC TGCAGGCCAA GAGACAACAG CGATTACAAA CCCCAGTCCC AGCCTTGCAG CTGACCTTGC AGGTGACGCC CTGCCTGGGT GTCTCGGTGC AGCTGCACAT CAGGGGCCTC TGCTGGACAG AAGCTCTGAG
Rhesus  AGCGGCATGT GCGAGAACCC TGCGGGCCAA GAGATGACAG CGATTACAAA CCCCAGCCCC AGCCTTGCAG CTGACCTTGC AGGAGACACC CTGCCCGGCT GTCCCGGTGC AGCTGAACAT CAGGGGCCTC TGCTGGACAG AAGCTCCGAG
Clustal ******* *  * *** * ** *** ****** ****  **** ********** ****** *** ********** ********** *** *** ** ***** ** * *** ****** ***** **** ********** ********** ****** ***

Human   TCCACACTTG GCCCCCAGGC GCTGGAGCTG GAGCACTGCC ACGAGAGGGG ATGCTGCCGT GGCTGCGCCA GCTTCAGCCC TTTCCCTGCA CCGAGATGTC CCAGTGAGCG GCTGGGTGCT CACAGTTCCC GTTGGGCCAT CAGAGGAAGA
Rhesus  TCCACACTCT AACCCCAGGG GCTGGGGCTG GACCACTTCT ACGAGAGGGG ATGCTGCCGT GGCTGCATCA GTTTCAGCCC TTTCCCTGCC CCGAGACGTC CCAGAGAGCG GCTGGGTGCT CACAGTTCCT GTTAGGCCAT CAGAGGAAGA
Clustal ********     *******  ***** **** ** **** *  ********** ********** ******  ** * ******** *********  ****** *** **** ***** ********** *********  *** ****** **********

Human   TCAAAGATCA ACCCTCCCCC ATGGGCTCCT GCCTGCCTGC CAGGTGGGTT TCCTGCCTGT CTCCCAGCCC CCAAGAGCTC CACGGACTCT GCCAGTAGCT GCTTTAAAGG AGGGAGGGAG TTTTCTGATC CTCTGGACAT CCCTGGGGCT
Rhesus  TCAAAGATCA ACCCTCCCCC ATGGGCCTCT GCCTG----C CAGGTGGGCT CCCTGCCTGT CTCCCGGCCC CCAAGAGCTC CACGGACTCT GCCAATAGCT GCTTTAAAGG AGGGAGGGAG TTTTCTGATC CTCTGGATAT CCCTGGGGCT
Clustal ********** ********** ******  ** *****    * ******** *  ********* ***** **** ********** ********** **** ***** ********** ********** ********** ******* ** **********

Human   GGAGCTATGG GCTGA
Rhesus  GGGGCTGTGG GCTGA
Clustal ** *** *** *****
```

ENSG00000187488:

```
Human   ATGTGTCCTC ATCTCAGGCC CGTGCCTGGG ACCCCGTGTC TGCCCAGGTG GGCAGCCTTG AGCCCAGGGG ACTCAGTGCC CTCCATGCCC TGGCTGGCAG AAACC-CTCA ACAGCAGTCT GGGCACTGTG GGGCTCTCCC CGCCTCTCCT
Rhesus  ACGTGTCCTC ATCTCAGGCC AGGTCCTGGT ACCCCATGTC TGCCCAGGTG GGCAGCCCTG AGCCCAGGGG ACTTAGTCCC CTCCATGACC AGGCTGGCAG AAACCTCCCA ACAGCGGTCT GGGCACCGTG GG-CTCTCCC CACCTCTCCT
Clustal * ******** **********  *  *****  ***** **** ********** ******* ** ********** *** *** ** ******* **  ********* ***** * ** ***** **** ****** *** ** ******* * ********

Human   GCCTTGTTTG CCCCTCAGCG TGCCAGGCAG ACTGGGGGCA GGACAGCCGG AAGCTGAGAC CAAGGCTCCT CACAGAAGGG CCCAGGAAGT CCCCGCCCTT GGGACAGCCT CCTCCGTAGC CCCTGCACGG CACCAGTTCC CCGAGGGACG
Rhesus  GCCTTGTTTG CCCCTCAGCG TGCCAGGCAG ACTGGGGGCA GGACAGCCAG AAGCTGAGAC CAAGGCTCCT TGAAGAAGGG CCCGGGAAGG CCCCGCCCTT GGGACAGCCT CCTCTGAAGC CC-TGCACGG CACCAGCTCC CCAGCAGATG
Clustal ********** ********** ********** ********** ******** * ********** **********    ******* *** *****  ********** ********** **** * *** ** ******* ****** *** **    ** *

Human   CAGCAGGCCG CCTCCCGCAG CGGCCGTGGG TCTGCACAGC CCAGCCCAGC CCAAGGCCCC CAGGAGCTGG GACTCTGCTA CACCCAGTGA AATGCTGTGT CCCTTCTCCC CCGTGCCCCT TGATGCCCCC TCCCCACAGT GCTCAGGAGA
Rhesus  CAGCAGGCCG CCTCCTGCAG CGGCTGTGGG TCTGCACAGC ACGG-----C CCGAGACCAC CAGGAGCTGG GACTCTGCTA CACGCAGTGA GACGCCATGT CCTTTCTCCC CC-TGCCC-- -------CCC TCCCCACAGT GCCCAGGAGA
Clustal ********** ***** **** **** ***** **********  * *     * ** ** ** * ********** ********** *** ******  * **  *** ** ******* ** *****           *** ********** ** *******

Human   CCCGTGGGGC ACGGAACAGG AGGGTCTGGA CCCTGTGGCC CAGCCAAAGG CTACCAGACA GCCACAACCA GCCCAGCCAC CATCCAGTGC CTGGGGCCTG GCCACTGGCT CTTCACAGTG GACCCCAGCA CCTCGGGGTG GCAGAGGGAC
Rhesus  CCCGAGGGGC ACGGTACAGG AGGGCCTGGA CCGCGTGGGC CAGCCAACAG CTACCAGACA GCTACAACCA GCCCAGCCAC CATCCAGTGC CTGGGGCCTA GCCACTGGCT CTTCACAGTG GACCCCAGCA CCTCGGGGTG GCAGAGGGAC
Clustal **** ***** **** ***** **** ***** **  **** * *******  * ********** ** ******* ********** ********** *********  ********** ********** ********** ********** **********

Human   GGCCCCCACG GCCCAGCAGA CATGCGAGCT TCCAGAGTGC AATCTATGTG ATGTCTTCCA ACGTTAA
Rhesus  GGTCCCCACT GCCCGGCAGA TGCACGAGCT TCTGGAGGGC AACCCATGTG ACATCTTCCA ACATTAA
Clustal ** ******  **** *****     ****** **  *** ** ** * ***** *  ******* ** ****
```

ENSG00000189223:

```
Human   ATGGCGAAGG GTACACATAT ACTCACAGAA ATGAGTTATT TAAAGAAGAC GGTCCAGATT CCACAGTCCA AAAAAAAAA- ---------- ---------- ---GAGGTTT GCCCTGCTTC TGAAGGAGTC AGGGTATGAA TGCAGAGGTC
Rhesus  ATGGCGAAGG ----ACATAT ATTCATAGAA ACGAGTTACT TAAAGAGGAT GGTCCAGATT CCACAGTCCA AAAAAAAAAA AAAAAAAAAA AAAAAGAGAG AGAGAGGTTT GCCCTTCTTC TGAAGGAGTC AGGATATGAA TGCAGAGGTC
Clustal **********     ****** * *** **** * ****** * ****** **  ********** ********** *********                                 ******* ***** **** ********** *** ****** **********

Human   TTCCTGTTGG CTCACATATC AGATCTTCAA CGTCAACATT AAATGCGTGA GTGATGCTTC CCCCTCTGTG AATGTGGCTT TGGGTATGGC TAATCCACAC CCTGCACTCT GGCCTTCAGA AGCCCAGGGA GAGAAGCCTC CCAGAAGCAA
Rhesus  TTCCTGTTGG CTTACATATC AGACCTTCAA TGTCAACAAT AAA--CGTGA GTGATGCT-C CCCCTCTGTG GATGTGGCCT TGGGTATGGC TACTCCACAC CCTGCACTCT GGCCTTCAGA AGCCCAGGGA GAGAAGCCAA CCAGGAACAA
Clustal ********** ** ******* *** ******  ******* * ***  ***** ******** * **********  ******* * ********** ** ******* ********** ********** ********** ********   **** * ***

Human   CCTTCCAGAA CCTTCTCCAT CCTCCCACCG ACCTCCCCAG CCCCTGTCCT CTCTTTGA
Rhesus  CCTTCCAGAA CCTTCTCCAT CCTCCCACCG TCCTCCCCAG CCCCTGTCCT CTCTTT--
Clusta  ********** ********** **********  ********* ********** ******
```

ENSG00000196273:

```
Human   ATGGGCATAG GTACTGGGCA CACCTCGATG AACAAGGGAG GTAAGGATGT GACTCTCCTA GAACTTTCTG TCGAGAAGAG GAGATGGAGA ATAAACATGG AAACAAGCAA GATCATTCTG GAGAAAATGC AATCAGATGA TGTGCTAGAT
Rhesus  ATGGGCGTAG ATGCTGGGCA CACCTCGATG AACAAGGGAG GTAAGGCTGT GAATCTCCTA GACCTTTCTG TCTAGAAGAG GAGTTGGAGA ATAAACGTGG AAACAAGCAA GATCATTCTG GAGAAAATGC AATCAGATGA TGTGCTAGAT
Clustal ****** ***  * ******* ********** ********** ****** *** ** ******* ** ******* ** ******* *** ****** ****** *** ********** ********** ********** ********** **********

Human   GGTAACAGAG AGAGGTCGAA TGAGAGAGAG GGCCGTGACA GCCTCTCTGA GAAGCTGAAA TCCAAGCAGA ACCTGAAAGA CGAGGAGAAA CTCAGATACA TAAAGACTGG GAAAAGCATT CAAGTAGAGG GAACAGTGCG TGCAAAGGCC
Rhesus  GGTAACAGAG AGAGGTTGAA TGAGAGAGAG GGCTGTGACA GCCTCTCTGA GAAGCTGAAA TCCAAGCAGA ACCCGAAAGA CGAGGAGAAA CTCAGATACA TAAAGACTGG GAAAAGGATT CAGGTAGAGG GAACAGTGCG TGCAAAGGCC
Clustal ********** ****** *** ********** *** ****** ********** ********** ********** *** ****** ********** ********** ********** ****** *** ** ******* ********** **********

Human   CTGAGGTGGG TGCAGTAG
Rhesus  CTGAG-TGGG TGCAGTAG
Clustal ***** **** ********
```

ENSG00000197916:

```
Human   ATGCAGCCCT GTACTCCTGG CCTCAAGCCA TCCTCCCACC TCAGCCTCCA GAGTAGCTGG GAGCACAAGT GTGCACCATC ACACCCAGCT AACGTTTATT TTTTTGTAGA GAGAGTCTCA CTCACTATGT TGCCCAGGCT GGTCTTAAAA
Rhesus  ATGCAGCCTT GACCTCCTGG CCTCAAGCCA TCCTCCCACC TCGGCCTCCA GAGTAGCTGG GAGCACAAGT GTGCACCGTC ACGCCCAGCT AATGTTTATT TTTTTGTAGA AGAGATCTCA CT----CCGT TGCCCAGGCT GGTCTTAAA-
Clustal ******** * *  ******* ********** ********** ** ******* ********** ********** ******* ** ** ******* ** ******* **********      ***** **      ** ********** *********

Human   CTCCTGGCCT CAACTGATCC T---CCTCCT TTGGCCTCCC AAAGTGCTGG GACCATAGGC ATGAGCTGCC ATTGCCAGCT AATTGTTTTA TTTTTACATT TGCTGAGGCA GGGTCTCGCT -TGTGGCC-G TGCAGATCTT GAACTCCTGG
Rhesus  CTCCTGGCCT CAACTGATCC TGTCCCTCCT TTGGCTTCCC AAAGCTCTGG GACCATAGGC CTGAGCTGCC ATTGCCGGCT AATT----TA TTTTTACATT TGCTGAGGTG GGGTCCCACT GTGTGGCCCA GGCAGATCTT GAACTCCTGG
Clustal ********** ********** *   ****** ***** **** ****  **** **********  ********* ****** *** ****    ** ********** ********   ***** * **  *******    ********* **********

Human   CCTTGAGCAG TACCTCCCTC CTGGGTCTCT TACAGTGCTG GGATGATAGG GGTGAGCCGC TGTGCCCGGC CCGGGGTTTC TTTTTGAAGT GGTGA
Rhesus  CCT------- ---------C CTCGATCTCT TAAAGTGTTG GGATGACAGG TGTGAGCCAC TGTGCCCGGC CCAGGGTTTC TTTCTGAAGT GGTGA
Clustal ***                 * ** * ***** ** **** ** ****** ***  ******* * ********** ** ******* *** ****** *****
```

ENSG00000198547：

```
Human   ATGTTTCCTA GGCCTGTCTT GAACTCCCGG GCTCAAGCGA TTCTCCTGCC TCAGCCTCCC AACATGCTGG ATCACAGGCA GTGGCCACCT CGCCTGGCCA GTTTTCCCTT TACAAAAACT GGAATGCTGA GCCGGGCCAC ATCAGTCTTG
Rhesus  GTGTTTCCTA GGCCTGCCTT GAACTCCCAG GCTCAAGTGA GTCTCCTGCC TCAGCCTCCC AGCATGCTGG GTCACAGGCA GTAGCCACCT TGCCTGGCCA GTTTTCCCTT TACAAAGACT GGAATGTTGA GCCCGGTCAC GTCAGCCTTG
Clustal  ********* ****** *** ******** * ******* **  ********* ********** * ********  ********* ** *******  ********* ********** ****** *** ****** *** *** ** ***  **** ****

Human   GCTGGACTCA CTGCCCACCT CTGGGACCTT GGCGGTGGGG CGGGAAGGAG GACCTCAAAG GCTCAGCGAG TCCACCCCCA GCCCAGCCAC CAGCGCCAGC CTCCTCCTCC GCAACACCCA GGCCCTTATC AGGAAAGGAT TTGGGTTGGT
Rhesus  ACTGGACTCA CTGCCCACCT CTGGGACTTT GGCGGTGGGG TGGGAAGGAG GACCTCAAAG GCTCAGCGAG TCCACTCCCA GCCCAGCCAC CAGAGCCAGT CTCCTCCTCC GCAACACCCA GGCCCTTATC AGGAAAGGAT TTGGGTTGGT
Clustal  ********* ********** ******* ** **********  ********* ********** ********** ***** **** ********** *** *****  ********** ********** ********** ********** **********

Human   GGGGAGGGGT -GGGGGGAAG TTGGAGGCCT CAGGCTCAGC AAGGTGGGTC GGAGAGATAG GGAAGTGGGG AGGGGGCTGC GGGCCCCTGC TGGGCGGGGG AGGGCAATGG GAGGGATGCC CAGAATGGGC ACGGTGGGGG ACTTTGGCCA
Rhesus  GGGGAGGGGC AGGGGGGAAG TTGGAGGCCC CAGGCTTGGC AAGGTGGGTC GGAGAGATAG GGAAGTGGGG AGGGGGCTGC GGGCCCCTGC TGGGTGGGGG AGGGCAATGG GAGGGATGCC CAGAATGGGC ACGGTGGGGT GCTTTGGCCA
Clustal *********   ********* *********  ******  ** ********** ********** ********** ********** ********** **** ***** ********** ********** ********** *********   *********

Human   AGCTCTGTCC TCGCTGGCCT GGACCTCAAC ATGTTTCCAG GACTTCTGCC TCCCATCCTT GCCAGGCAAG TTGCCTGCAC CTTTAATTAG CAAGCAGCAG TTTCTCTCCA ATTCATCACG GTCGCTGTTT AATTAG
Rhesus  AGCTTTGTCC TCGCTGGCCT GGCCCTCAAC ATGGTTCCAG GACTTCTGCC TCCCATCCTT GCCAGGCAAG TTGCCTGCAC CTTTAATTAG CAAGCAGCAG TTTCTCCCCG ATTCATCACG GTCGCTGTTT AATTAG
Clustal **** ***** ********** ** ******* *** ****** ********** ********** ********** ********** ********** ********** ****** **  ********** ********** ******
```

ENSG00000203930：

```
Human   ATGCCACTGG AAAAATTCGT AGACATGGAA TTCCTGGATC AAATGATACT AAGGGGGACT TGCCTGGAAG CAGACTTGTG TCCGCAGTAT ACAAACTCAC CCTTTAATGC ACACACTGAC CAGATAACAC AAACACA--T TTACCCCCAC
Rhesus  GTGCCGCTGG AAAAATTCAT AGACATGGAA TTCGTGGATC AAATGATACT AAGGGAGACT TGCCTGGAAG CAGACTTGTA TCCGCAGTGT ACAAACTCAT CCTTTAGTGC GCACACTGGC CGGATAACAC AAACACACAT TTACTCCCAC
Clustal  **** **** ******** * ********** *** ****** ********** ***** **** ********** *********  ******** * *********  ****** ***  ******* * * ******** *******  * **** *****

Human   AGCACCTAGA CTCTTCTCAC AGTACACACA CTTGCCTTCC AGTACACAGA CATATCCAAA GGCACACCGA CAAGCACCAC AGCAAAAAGA TGTTCCCCAG AACACATGGA CACCCCAATC CCCGTATCAT TAGACTCTCC CAGAGGCACG
Rhesus  AGCACACAGA CACTTTCCAC AGTACACACA CTTGCCTTCC AGTACACAGA CATATCCAAA GGCACACCGA CAAACACCAC AGCAGAAAGA TTTTCCCCAG AACACACGGA CACCCCACTC CCCGTATCAT TAGACTCTCC CAGAGGCACA
Clustal *****  *** * ***  *** ********** ********** ********** ********** ********** *** ****** **** ***** * ******** ****** *** ******* ** ********** ********** *********

Human   TATGTACTGC TTAA
Rhesus  TATGTACTCC TTCA
Clustal ******** * ** *
```

ENSG00000204091:

```
Human     ATGAAGAGGA GGGAGGCAGT CTGCGCGCAC CGCCATTTT- CTAGGAACTG GGAAGC---- -----CCCCC CACCCCTTAG GAAGATCCAT CCCTGTGGAA CCTTGCCCAG GCTTACCAGC CTTTGCTGAG GTTGATCTAT T-------GT
Rhesus    ATGAAGAGGA GGGAGGCAGT CTGCACGCAC CGCCATTTTT CTAGGAACTG GAAAGCGCCT CCCGCTCCCC CCGCCCTTAG GAAGATCCAT CCTTGTGGAA CCTTACCCAG GCTTACCAGC CTTTGCTGAG CTTGATCTAT TTGGACTAGT
Clustal   ********** ********** **** ***** *********  ********** * ****           **** *  ******* ********** ** ******* **** ***** ********** **********  ********* *       **

Human     ---------- ---------- ---------- ---------- --CCCTCCTT GTCCCCATCA AAATATCCAG CACTCCACCT TCAGGGAGTA GACTTGACCC TCAAATAGCA AGTTCAGCCT TCCCAGGTCT AGGTTCCCTG GGAGGTCAAG
Rhesus    ATCAGTCAGT AGGACTTGAG ACGTTGGTTC TCTACACTTG CACCCTCCTT GTCCCCATCA AAATATCCAG TACTCTACCT CCAGGGAGTA GACTTGACCC TCAAATAGCA AGTTCAGCCT TCCCAGGTCT AGGTTCCCTG GGGGTTTAAG
Clustal                                                 ******** ********** **********  **** ****  ********* ********** ********** ********** ********** ********** ** * * ***

Human     ATTCGTCTGG TTCCTTAGTA CAGAGGGCTA GCTGTGAGTT GGAATCCCCC TATGAGCTTT AG
Rhesus    ATTCGTATGG TTCCTTAGTA TAGAGGGCTA GCTGTGAGAT GGAATCCCAC TATGAGCTTT AG
Clustal   ****** *** **********  ********* ******** * ******** * ********** **
```

ENSG00000204292:

```
Human     ATGACCTTTT TCTCAACCAT GCGCAAGTGG TATTTCTGCG TGCTTCAAAT ATCACAGGCC GGATTGTCAG GAAACCAAGG AGGGGCCTTT TGGAAGAGAA TGATGCTGAG C-ATCCGGGT CCCTGGACAG CCAGCAGCCC TGAGGCAGAC
Rhesus    ATGACCTTTT TCTCAACCAT GTGCAAGTGG TATTTCTGCG TGCTTCAAAT ATCACAGGCG GGATTGTCAG GAAGCCGAGG AGGGGCCTTT TGAAAGAGAA TAATGCTGAG CCATCCGGGA CCCTGGATGA C-AGAAGCCC TGAGGCAGAC
Clustal   ********** ********** * ******** ********** ********** *********  ********** *** ** *** ********** ** ******* * ******** * *******  *******    * ** ***** **********

Human     AGAAGTTTGG ACAACTCAGG ACAGGGAGCT TGGAGGGCAG CCCCTACATG TCGGAGTGGT CCCTACCTGT GTTGCCTCAA CTCTTCCTTC AAGGCAAGAG CCGCTAAGGA CAG--TCAGG ACATCCTACC GCCCAAGGCT GTTGAGCTCG
Rhesus    AGAAATTTGG ACAACTCAGG ACGGGGAACT TGGAAGGCAG CGCCTACAGG TCTGAGTGGT CCCTGCCTGT GTTGACTCAA TTCTTCCTCC AAGGCAAGAG CCGCTAGGAA CAGAGTCAGG ACATCCTACT GCCCAAGGCT GTTGAGCTCG
Clustal   **** ***** ********** ** **** ** **** ***** * ****** * ** ******* **** ***** **** *****  ******* * ********** ****** * * ***  ***** *********  ********** **********

Human     GCCGGCCAAG GGCTACAGGA AGGATGGCTG GGGAGGCCTC AGGAAACTTA CAGTCACAG- ---------- ------CGGA AGGCAAAGAG GAAGCAGGCA GCGGCTTCGT GGCAGGAGCA GGAGGAAGGC GGTGGGGAGG TGCTACATAC
Rhesus    GCCA--CGGG CTGTACAGGA AGGATGACTG GGGAGGCCTC AGGAAACTTA CAATCACAGG AAACTTACAA TCACGATGGA AGGCAAAGTG GAAGCAGGT- GTGGCTTCAT GGCAGGAGCA GGAGGAAGGT GG-GGGGAGG TGCTACATAC
Clustal   ***   *  *    ******* ****** *** ********** ********** ** ******                           *** ******** * ********   * ****** * ********** *********  ** ******* **********

Human     TTTTAAACAA CTGGATCTTG TGA
Rhesus    TTTTAAACAA CTGAATCTTG TGA
Clustal   ********** *** ****** ***
```

ENSG00000204380:

```
Human     ATGGCGGCTC TCATCTGCCC TGCCTCTCCG CAGCACTTTC CTTTTCTCCA C-AGCTTCTG GGACCCCACC TGGCTTCTCT CTCACCTTGC TACTTCTCAG ACTCATCTGC CCATGGGCAC CTCCAGGAGT GCCCCAGGTC CTGTCTTGTC
Rhesus    ACGGCGGCTC TCATCTGCCC TGCCTCTCTG CACCACCTTC CTTTTCTCCA CTGGCTTCTG GGACCCCACC CGGCTTCTCT CTCATGTCGC -GCTTCTCAG ACTCATCTGC CCAAGGG--- -------AGC ACCCCAGGTC CTGTGTTGTC
Clustal   * ******** ********** ******** * ** *** *** ********** *  ******* **********  ********* ****  * **   ******** ********** *** ***           **   ********* **** *****

Human     TTCATCTTTG CACTCTCCAA GGTGCCTTCT ---GCTCCTT GTCTTTAATA CAACCTATGG ACACAGGGCC ATAGGTTGGC ACACATCTGC CTTTAGCCCT GACTGCTCTC TAGAATTGCG GATTCTTTTC TCCAAT-GCT TTCTTGACAC
Rhesus    TTCATCTTAG CACTCTCCGA GGTGCCTTCT TCTGCTTCTT GGCTTTAATA CAACCTATGG ACCCATGGCC ATAGGCTGGC ACACATCTGC CCTTAGCCCT GACTGCTCTC TAGAATTGCA GATTCTTTCC TCCAATTGCT TTCTTGACAC
Clustal   ******** * ******** * **********    *** *** * ******** ********** ** ** **** ***** **** ********** * ******** ********** *********  ******** * ****** *** **********

Human     TGGCACATAG ACAGCTAATT AGACTTCTCA AACTGGACAT TGTCAAAACT CTGAGCTGCT CACC------ --CTTCCAAG CATTCCTGTC CCTTCCCCCC ATCAACAGCA CTTCTGTGCT TGCAGCTGAT CCAGCCAAAG ATCTAGGTGT
Rhesus    CGGAACGTAG ATAGCTAATT AGACTTCTCA AACTGGACAT TGTCAAAACT CTGAGCTGCT CACCTGCTCA CCCTTCCAAG CATTCTTCTC CCTTACCCCC ATCAA--GCC CTTCTGTGCT TGCAGCTGAT CCAGCCAAAG ATCTAGGTGT
Clustal    ** ** *** * ******** ********** ********** ********** ********** ****         ******** ***** * ** **** ***** *****  **  ********** ********** ********** **********

Human     ATCCTTATTT CCCCCCTTTC CTCGCTCTTA A
Rhesus    ACCCTTATTT CCCCCCTTTC CTCACTCCTA A
Clustal   * ******** ********** *** *** ** *
```

ENSG00000204626:

```
Human     ATGCACAGCC TGCCACGGAG TGGCTCTATC AGGCGCACAC ACAGCGACAC ACAGGCCACT GGCTGGCCTC CTCCCCAGCG CATTGGGGAC AGCCCAGGCC CTTCTCCAGC ATTTCTGTCC TGCCCACCTT CCCTCTGTGG AGGAGCAGCC
Rhesus    ATCCACAGCC TGCCA----- --------TC AGGCGCACAC AGCGACACAC ACAAGCCACT GGTTGGCCTC CTCCCCAGCG CCCTGGGGAC AGCCCAGGCC CTTCTCCAGC GCTTCTGTCC TGCCCACCTT CCCTCTGTGG AGGAACGGCT
Clustal   ** ******* *****              ** ********** *  *  **** *** ****** ** ******* ********** *  ******* ********** **********   ******** ********** ********** **** * ** 

Human     CAGACAGGAG ACCCTGTGGC CCTGCCCCAT GGCCCAGAGA AATGGGTGTG GGGCGGTGGC CTCTCCCCCA GGAATCCTCA TTCCTGGGGC ATCAAGGCCC ACGGACTTAG ACCACCCTGG GCCCCCAGGC TAGAAAGATG CATGGTCCCA
Rhesus    CAGACAGGAC ACCCCGTGGC CCTCCCCCAG GGCCCAGAGA AGTGGGTGCG GGGCGGTGGC CTCTCCCCCA GGAAT----- -----GAGGC ATCAAGGTCG ACGGACTTAG ACCACCCTGG GCCCCCAGGC TAGGAAGATG CATGGCCCCA
Clustal   *********  **** ***** *** *****  ********** * ****** * ********** ********** *****           * *** ******* *  ********** ********** ********** *** ****** ***** ****

Human     GAGTCAGAAT GGGCACCATG GCAACCCCAG CTACCCTGTG AGCCGAAGTG GCTGGGGAGC AGGAAGTCGA AGCCTCACAG AGAAAGTGGT CTCCGGGGAG GAGGACCCAG CAGATGTGCA AAGAG----- ---AGGAACA CACTCCTGTG
Rhesus    GAGTCAGAAT GGGCACCATG GCAACCCCGG CTACCCTGGG AGCCGAAGTG GCTGGGGAGC AGGAAGTAGA AGCGTCACAG AGAAAGCGGC CCCCGGGGAG GAGGACCCAG CAGACATAGA AAGGGGAGAA GACAGGAGCA CACGCCTGTG
Clustal   ********** ********** ******** * ******** * ********** ********** ******* ** *** ****** ****** **  * ******** ********** ****  *  * *** *          **** ** *** ******

Human     GCCCCAGAGA GAGTGGTGGC CCGGACACCT GCCACCTCCC CTGCCACTGA
Rhesus    GCCCCAGAGA GAGTGGCAGC CCAGACACC- GCCAGCTCCC C-GCCACTGA
Clustal   ********** ******  ** ** ******  **** ***** * ********
```

ENSG00000204666:

```
Human     ATGTCACTAG ACAGTGGGGC CCTCAAGGAC CTGGCAAATG GGGGCTTGGA CCTCGGATCG GCCTCCCACG CGAAGCTTGC TCCCCACCAG CATCCCCACG TTGGTGGCGA CGCTGCCCCG GCCCCACGGA TACTTCCGCG CCTGTCAGAC
Rhesus    ATGTCACTAG ACAGTCGGGC CCTCAAGGAC CTGGCAACTG GAGGCTTGGA CCTCGGGTCG GCTTCCCACG CGAAGCTTGC TCCCCACCTG CATCCGCACG TTGGTGGAGA CGCTGCCCCG GCCCCACGGA TGCCTCTGCG CATGTCAGAC
Clustal   ********** ***** **** ********** ******* ** * ******** ****** *** ** ******* ********** ******** * ***** **** ******* ** ********** ********** * * ** *** * ********

Human     TCCCTGATGA ACTACCCTTC CCAGAGTACC GCGGGAGCTC GGGCTCCTGA GGGCGACGGT CCTCTGATGG CAGATGCGGG AGAAACTCTG GCGTCAGGCG GCCCTCGCGT GGAGCACACG AAGTCGTGGC TTATTCTGGC TTCAGTATGT
Rhesus    TCCCTGATGA ACTACCCTTC CCAGAATCCC GCGGGAGCT- -----GCTTA GGGCGACGGT CCTCTGATGG CCGATGCGGG AGAAACCCTG GCGTCAGGCG GCCCTCGAGT GGGGCACACG AAGTCGTGGC TTATTCTGGC TTCAGTATGC
Clustal   ********** ********** ***** * ** *********       ** * ********** ********** * ******** ****** *** ********** ******* ** ** ******* ********** ********** ********* 

Human     GGGGTGGAGA AGGCGATCCA CGCAGCTGCG TCTATTTCCT GTGGATCAAT CGCAAAATAC GTTCTGTAA
Rhesus    GGGGTGGAGA AGGCGATCCA CGAGGCTGCC TCTGTTTCCT ATGGATCAAA CGCAGAAATC GTTCTGTAA
Clustal   ********** ********** **  *****  *** ******  ********  **** **  * *********
```

ENSG00000204674:

```
Human     ATGCCCCTCC CAGGAACCCC AGGACCTGTC ACCACCTCCC CACAGACCCC CACCCCCCGC CCCCTCACCA CCGACTGGCG CATCTTGTCC GGCAAGGGGT CCGGGGGCTC GGCCCGGGCT GTCTCCAAGC TGCGCTCCTC CAGCTCAGGG
Rhesus    ATGCCCCTCC CAGTACCCCC AGAACTTGTC ACCACCTCCC CACAGAACCC CACCCCCCGC CCCCTCACCA CTGACTGGCG CATCTTGTCC GGCAAGGGGT CCGGGGGCTC GGCCCGGGCT GTCTCCAAGC TGCGCTCCTC CAGCTCGGGG
Clustal   ********** *** * **** ** ** **** ********** ****** *** ********** ********** * ******** ********** ********** ********** ********** ********** ********** ****** ***

Human     AACAGCTTGC TCCGGATCAG AGACCTGGGG GTGAGGAAGA GTCAGGAGGA GGCCGCCCCT CCTTCCCCCC GGCCCCAGTC ACGGGCACAT GCACAGACCA CAAACCCCTA CTGGGCAGAC ACAAACACAC GACCCGGAGC ACAC------
Rhesus    AAGAGCTTGC TCCGGATCAG AGACCTGGGG GTGAGGAAGA GTCAGGAGGA GGACGCCCCT TCTTCCCC-- GGCCCCAGTC ACAGGCACAT GCACAGACCA CAAACCCCTA CTGTGCAGAC ACAAACACAC GACCCGGAGT ACACACACCA
Clustal   ** ******* ********** ********** ********** ********** ** *******  *******   ********** ** ******* ********** ********** *** ****** ********** *********  ****

Human     ---------- ---------- ------CCAC CAGAGCACGG TGGGGTCGCT GCTCTGCCGG CTCAGGTGGT AGGACAGTGC CACCAGGAGG CCACAGAAGA CTGA
Rhesus    GCGCGAGCAC AGGCGCGTGC GGGAACCCAC CAGAGCACGG TGGGGTCGCT GCTCTGCCGG CTCAGGTGGT AGGACAGCGC CACCAGGAGG CCACAGAAGA CTGA
Clustal                                 **** ********** ********** ********** ********** ******* ** ********** ********** ****
```

ENSG00000205056：

```
Human    ATGTTCAACA AATGCTCCTT TCATTCCTCT ATTTACAGAC CTGCCGCAGA CAATTCTGCT AGCAGCCTTT GTGCTATTAT CTGTTTTCTA AACTTAGTAA TTGAGTGTGA TCTGGAGACT AA-CTCTGAA ATAAATAAGC TGATTATTTA
Rhesus   ATGCTCAATA AATGCTCCTT TCATTCCTCC ATTTACAAAC TTGCCGCAGA CAATTCTGCT AGCAGCCTTT GTGCTATTAT CTGTTTTCTA AACTTAGTAA TTGAGTGTTA TCTGGAGACT AAACTCTGAA ATAAATAAGC TGATTATTTA
Clustal  *** **** * ********** *********  ******* **  ********* ********** ********** ********** ********** ********** ******** * ********** ** ******* ********** **********

Human    TTTATTTTCT CAAAACAACA GAATACGATT TAGCAAATTA CTTCTTAAGA TATTATTTTA CATTTCTATA TTCTCCTACC CTGAGTTGAT GTGTGAGCAA TATGTCACTT TCATAAAGCC AGGTATA--- -CATTATGGA CAGGTAAGTA
Rhesus   TTTATTTTCT CAAAACAACA GAATATGATT TAGCAAATTA CCTCTTAAGA TATTATTTTG CACTTCTATA TTCTCCTACC CTGAGTTGAT GTGTGAGCAA TATGTCACTT CCACAAAGCC AGGTATATAT ACATTACGGA CAGGTAAGTA
Clustal  ********** ********** ***** **** ********** * ******** *********  ** ******* ********** ********** ********** **********  ** ****** *******     ***** *** **********

Human    AAAAACATAT TATTTATTCT ACGTTTTTGT CCAAAAATTT TAAATTTCAA CTGTTGCGCG TGTGTTGGTA A
Rhesus   AAAA-CATAT TATTTATTCT AGGTTTTTGT CCAAGAGTTT TAAATTTCAA CTGTTGTGCA TGTGTTGGTA A
Clustal  **** ***** ********** * ******** **** * *** ********** ****** **  ********** *
```

ENSG00000205056：

```
Human    ATGGAGAGGA GGAAAAGTTT TGCATTGGAC CAAATCACTG CCTTAGAAGG AAAGAGATTG CAATCCCATT CATTTTTAAG ATCTGCAATG GGTTGGGATG ATACCCATTT A--------- ------AAAA AGAAAAAAAA AGAGGTTACC
Rhesus   ATGGAGAGTA GGAAAAGTTT TGCATTGGAC CAAATCACTA CCTTAGAAGG AAAGAGATTT CAATCCCATT CATTTTTAAG ACCTGCAATG GGTTGGGATG ATACCCACTG AGGTCTCTTG GCAGTTAAAA AAAGAAAAAA AGAGGTTACC
Clustal  ******** * ********** ********** *********  ********** *********  ********** ********** * ******** ********** ******* * *               **** * * ****** **********

Human    TCTTTGGCCT CTTTGGCAAA GGATATGAAC TAG
Rhesus   TCTTCGGCAT CTTTGGCAAA GGATATGAAC GAG
Clustal  **** *** * ********** **********  **
```

ENSG00000205373：

```
Human    ATGACCGCAA CCCTCGGGTC CCGAGGCGCC GCTCCAGGCG GGAGCGCCAA CTCCCACGGC CCTTCCGACC CCCGCGTCCC ATTGTTCCGA ATCTCACTCA CCTCGGAGGA TGCCGGCGCA GGCCTGGCTG GGGCGCTCGC CGCGAGCTCC
Rhesus   AAACCCGCAG CCCTCGGGTC CCGAGGCGCC GCTCCAGGCG GGAGCGTCAG CTCATCCGAC CCTTCCGACC CCCACGTCCC ATTGTTCCGA ATCTCACTCA CCTCGGAGGA TGCCGGCGCA GGCCTGGCTG GGGCGCTCGC CACGAGCTCC
Clustal  *   ***** ********** ********** ********** ****** **  ***    ** * ********** *** ****** ********** ********** ********** ********** ********** ********** * ********

Human    CGGCAGCCCA GAAAGCTAGG TCGGTGTCGG CAGGATCCGC GAAGGCGTAC CCGGCGACTT CTCCGCCTAC CCTCCGAAGC GGGAGCAGGC GGGGGCGCTT CACCCTCCTC GCGAGACAGC GGAGACCCCA GCGGCGGCTG CGACCCCTGT
Rhesus   CGGCAGCCCA GAAAGCTAGG TCGGTGACGG CAGGATCCGC GAAGGCGTAC CCGGCGACTT CTCCGCCTAC CCTCCGAAGC GGGAGCAGGC GGGGGCGCTT CACCCTCCTC GCGAGACAGC GGAGACCCCA GCGGCGGCTG CGACCCCTGT
Clustal  ********** ********** ****** *** ********** ********** ********** ********** ********** ********** ********** ********** ********** ********** ********** **********

Human    GACGCAAAGG GCTCATCACC AATGGCAGCT CGGAAGCAGG GCAGAGAGCG AGACCCACCC CCCAGCTCCC G-CGCATCAA AACACAACAC CCGCCTTCCT CACGCATGCG CAGCAGATGG ACGCCGGCCT CGGGGGCGGG GCTCAGCGCA
Rhesus   GACGCAAAGC GCTCATCACC AATGGCAGCT CGGAAGCAGG GCAGAGAGTG AGACCCACCC ACCAGCTCCC GGCGCGTCAA AACACAACAC CCGCCTTCGC CACGCATGCG CAGGAGATGG GCGCCGGCCC CGAGGGCGGG GCTCAGCGCG
Clustal  *********  ********** ********** ********** ******** * **********  ********* * *** **** ********** ********   ********** *** ******  ********  ** ******* *********

Human    CCGGCCCAAA TGCGGCCCCT CCCCCGCCGC TCCAGACCCC GGAGGAGGGC AGCGCGACCC TCCCCATCTT GTTACTGGTA CTCAGCCACT GGGGACACTG ACTGAATGTT CGCCCCTAAC TTACCCGGAA AACACGAGAA CTAGTTCGAT
Rhesus   CCTGCCGAAA CGCGGCCCCT CCCCCGCAGC TCCAGACCCG GGATGAGGGC AGTGCGACCC TCCCCATCTT GTTACTGGTA CTCAGCCGCT GGGGACACTG ACTGAATGTT CGCCCCTAAC TTACCCGGAA AACACGACAA CTAGTTCGAT
Clustal  ** *** ***  ********* ******* ** *********  *** ****** ** ******* ********** ********** ******* ** ********** ********** ********** ********** ******* ** **********

Human    GTCGTTTCAA CCTTACCAAA CTCTGCGGAA ACTTAGGAAA TTATCTTTAG TGTCTAGATA G
Rhesus   GTCGTTTCAA CCTCACCAAA CTCTGCGGGA ACTTACGAAA TTGTCTCTAG TGTCTAGATA A
Clustal  ********** *** ****** ******** * ***** **** ** *** *** **********
```

ENSG00000205557：

```
Human    ATGTTTCGAT TTCACACAAA GAAAGAGCAC ACGTCCACCA TCTTCAGTGG GGGCTGTCTT TTGCTTCACT GGC------- --AAGCAGGC ACTGAATTTT TCTTGCATGA CAAATCTGGA GGTTTACTGG TGAGAGAGCC AATGGGCATT
Rhesus   ATGTGTCAAT TTCGCACACG GAAAGAGCAC ACGTCCACCA TCTTTGGTGG GGGGTGTCTT TTGCTTCACT GGCTTCACTG GCAGTCGGGC ACTGAATTTC TCTTGCATGA CAAATCTGGA GGTTTACTGG TGAGAGAGCC AATGGGCATT
Clustal  **** ** ** *** ****  ********** ********** ****  **** *** ****** ********** ***          *  * *** *********  ********** ********** ********** ********** **********

Human    TTTTCCTGGA AAGAGTACAG CTCCATACCC AGTCCTAACC CAACAGTGAT -------ATT TATCACTTTG GGGCAGGGCT GTATAGAGTG TGTGTGTGTG TGTGTGTGTG TGTGTGTGTG TGTGTGTGTG TGTGTGCGGG TTGGGGTGGT
Rhesus   TTTTCCTGGA AAGAGTACAG CTCCATATGC AGTCCTAACC CAATAGTGAT TCCAGGAATT TATCACTTTG GGGCAGGGCT GTATAGAGTG TGTGCGTGTG TGTATGTGTG TGCGTGCGCA TGCGCGTGCG TATGTGGGGG TTGGGGTGGT
Clustal  ********** ********** *******  * ********** *** ******        *** ********** ********** ********** **** ***** *** ****** ** *** *   ** * *** * * **** *** **********

Human    GTTGGGCCAT CTCTGGCCTG TTACTAAGGT AACTAGGACT ATTTGTGTTC CAGCAGTCAT AGCCTGTGAT TGTGGGTGCA TCAGTTCTCT GCCTAGATCT CTTGTTACCT TGTCTGCACA TCAAGGGAGG GAGTTGAGCA CAGATACTTG
Rhesus   GTTGGGCCAT CTCTGGCCTG TTACTAAGGT AACTGGGACT ATTTGTGTTC CACTAGTCAT AGCCTGTGAT TGTGGGCACA TCAGTTCCCT GCCTAGATCT CT-ATTACCT TGTCTGCACA TCAAGG---- GAGTTGAGCA CAGATACTTG
Clustal  ********** ********** ********** **** ***** ********** **  ****** ********** ******  ** ******* ** ********** **  ****** ********** ******     ********** **********

Human    TCAAGGGCCA TTGTAG
Rhesus   TCAAGGTCCA TTGTAG
Clustal  ****** *** ******
```

ENSG00000205913：

```
Human    ATGTCTATGG CTGCCTGTCC TGAGGCTCCG GAGCCCTCAT TTCTTCGTGA AGTCCC---- -------CAG CTCCCCTGCC TCCACTCAAT GGCACCGGCC CTGCAACTTT AGGCAG---- -GTCGAAGCC AACCCAAGGA AAGAACCTAA
Rhesus   ATGGCTACGG CTGCCTGTCC TGAGCCTCCC GAGCCCTCAT TTCTTCGTGA AATCCTGTTT CCCCACTCAG CTCCCCCGCC TCCACTCAGT GGCACCGGCC CTGCAGCTTC AGGCGGCAGG CGTCAAAGGC AACCCAAGGA AAGAACCTAA
Clustal  *** *** ** ********** **** ****  ********** ********** * ***               *** ****** *** ******** * ********** ***** *** **** *      *** *** * ********** **********

Human    GAACCTCGTT TGGAGGGATG TCAGCTTGGG CCAGACCAGC CGCACCCCGC GGGGCTCAGG CTTGGAACTG GTGAGGGTGT GTGGTGGGGG TATGCAGAGG GATAAGACCG TGGTAGAGGA GAGGGTTGGT GAGGAGAGAG AGAGAGAGAG
Rhesus   GAACCTCGTT TGGAGGGATG TCAGCTTGGG CCAGCCCAGC CGCACCCCGC AGGGCTCAGG TTTGGAACTG GTGAGGGTGT GTGGTGGGGG TGTGCAGAAG GGTAAGACCG AGGTAGAGG- ----GTTGGG GAGGGGAGA- ---------G
Clustal  ********** ********** ********** **** ***** **********  *********  ********* ********** ********** * ****** * * ********  ********      *****  **** ****          *

Human    AGAGAGAGTC TGGGGGGAGC GGGCAAGCAT GGGGAGATGA
Rhesus   AGAGAAAGTC TGGGGGGAGG GGGAAAGCTT GGGGAGATGA
Clustal  ***** **** *********  *** **** * **********
```

ENSG00000206028:

```
Human    ATGGAGAGGA TTCTTCCCCA GCTTCCTTCC AGAG---GAC ACAAAAGCTC AGAGCTCCAC AGTCTAGAGT CTAGACCAAC AGGCCTCCAC ACTCACGTCC CAGAGATTTC CCTGGTCCCA CCTACTCCCA GTGGCAACCA GACTTCTGCA
Rhesus   ATGGAGAAGA TTCTTCCCGA GCTTCCTTCC AGAGAAGGAC ACAAAAGCTC AAAGCTCCAC AGTCTAGTTT CTAGACCCAC AGGCCTCCAC ACTCACGTCC CAGAGGTTTC CCTGGTCCCA CCTACTCCCA GTGGCAACCA GACTTCTGCA
Clustal  ******* ** ******** * ********** ****   *** ********** * ******** *******   * ******* ** ********** ********** ***** **** ********** ********** ********** **********

Human    CATAGGAGAG ATGTCATACT CAGAGTCCAG CCTCCCACAT CCACAGGACC ACCTCTTCCT CCTCTGAGTC TTGTTAATAG GGCCATCCCC TGCCTTAGAC CTGGCCCAGT GGACTCTGAT CTTACA---- GCCAATATGG GGCAGCAAAG
Rhesus   CAGAGGAGAG ATGTCATACT CAGAGTGCAG CCTCCCCCAT CCACAGGACC ACCTCCTCCT CCTCTGAGTC TTGTTAATAG GGCCATCCCC TGCCTTAGAC CTGGCCCAGT GGACTCTGAT CTTACAAACA GCCAATATGG GGCAGCAAAG
Clustal  ** ******* ********** ****** *** ****** *** ********** ***** **** ********** ********** ********** ********** ********** ********** ******     ********** **********

Human    TGGGACATCT GTCTACAGGG CCAGTAGCCC CAGGTCATCT GCTTGCCAAA AGGAGGGGGA CCAGCCCCCG GGGGGAGCCC AGAGCTCGGC AGGGCTGGGG TTAGTAAGAA GAGAAAACAG GGTTAGTAGG GGCTGGGTTA GTAAGTCAGA
Rhesus   TGGGACATCT GTATACAGGG CCAGTAGTCC CAAGTCATCT GCTTGCCAAA AGGAGGGGGA CCAGCCCCCG GGGGGAGCCC AGAGCTCGGC AGGGCTGGGG TTAGTAAGAA GAGAAAACAG GGTTAGTAGG GGCTGGGTTA GTAAGTCAGA
Clustal  ********** ** ******* ******* ** ** ******* ********** ********** ********** ********** ********** ********** ********** ********** ********** ********** **********

Human    GCACAGCACC AGCGGACAGG GCACCTCAGC AGACACACAC AGGAGTCGCT AA
Rhesus   GCACAGCACC AGCGGACAGG GCACCTCAGC AGACACACAC AGGAGTCGCT AG
Clustal  ********** ********** ********** ********** ********** *
```

ENSG00000205965:

```
Human   ATGTGGTGGC ATTTGTGCAC TCACAGTGCT GTGCACCCAC CCACACCGTC TAGTTTCAAA AGGCATTCAT CTCCCCAGAA GAAACCTCCC GTCCTCATTA AGCAGTTACC CCTCCTTGGT ATCCCCCAAG CCCCTCTC-- ----------
Rhesus  ATGTGGTGGC ATTTGTGCAT TCACAGTCCT GTGCAACCAC TCACGCCATC TACTTTCAAA ATGTTTTCAT CTCCCCAGAA GAAACCTCCC ATCGTTGCTA AGCAGTTACC CCTCCTTGGT ATCCCCCAAG CCCCTCGTTT AATGAAAGGG
Clustal ********** *********  ******* ** ***** ****  *** ** ** ** ******* * *  ***** ********** **********  ** *   ** ********** ********** ********** ******

Human   ----CTGGGG TCCGAAGAGG GACTTGCCAG TGAGCGGAGC TCTGATAATA AGGAATCAGG CACCCA-CTG CTGGTCCAGG CCTGGGTTGG TTTTCCACCC AGCAGAGGTG GCAGAGCCAG GAGGGTCTGG GAGCGCTACA GGGGAGCCCC
Rhesus  GAAACTGAGG CCCGGAGAGA GACTTGCCAG TGGGCGGAGC TCTGAGAA-- -GGAATCAGG CACCCATCTG CTGCTTCAGT CCTGGGTTGG TTTTCCACCC AGCAGAGGTG ACAGAGCCGG GAGGTTCTGG GAGCGCCACA GGGGAGCCCC
Clustal     *** **  *** ****  ********** ** ******* ***** **    ********* ****** *** *** * ***  ********** ********** **********  ******* * **** ***** ****** *** **********

Human   ATGCTTGCCG CCGGAGCCCT GCCCCGCCCC GAGCTTCCCC ACCAGGGGGC AGCAGAGAGC TTTCCAGAAC CCGCCGCGGG GCTGGAGGGA AGCAGTGGCT CAGAGCTGCT GACAAACCTC AT----GTTG ACCCCAGACC GCTGTCTCTG
Rhesus  ATGCTTGCAG CCAGAGCCCT GCCCC----- GAGCCTCCCC ACCAGGGGGC AGCAGAGCGC TTTCCAGAGC CGGCCGCGGG GCTGGCGGGA AGCAGTGGGT CAGACCCGCT AACAAGCCTC ATTCATGTTG ACCCCAGATC GCTGTCTCTG
Clustal ******** * ** ******* *****      **** ***** ********** ******* ** ******** * * ******** ***** **** ******** * **** * ***  **** **** **     **** ******** * **********

Human   TGGGTTGGGC TTGGGAATTG GAGAGGAGGC CGCATGATTG GAAACATGAA GACGGCACGG CCTGGCTGGA GCAGCGGGAA GCGTCGTCAC GGTCACTGA
Rhesus  TGGGTTGAGC TTGGGAATT- ----GGAGGC CACATGATTG GAAACATGAA GACGGCTCAG CCTGGCTGGA GCAGCGGGAA GCGCCATCAC GTTTACTGA
Clustal ******* ** *********      ****** * ******** ********** ****** * * ********** ********** *** * **** * * *****
```

ENSG00000206096:

```
Human   ATGGAAGGTG ATTGTCTCAA GGTGGTCTGC TCTTCGCAAC TCTCCTTCTT TCTGGTAACC TCGCCCAGCA TCCCTCTAGG TCGAGGGAGG GGTGGTGTTG GCCTCATTGC TTTTAACCTG GATTCCTGTA CCATCCCTCA TGGTTCTCTC
Rhesus  ATGGAAGGTG ATCGTCTCAA GGTGGTCTGC TCTTCGCAAC TCTCCTTCCT TCTGATAACC TCGCCCAGCA TCCCTCTAGG TTGTAGGAGA AGTGGTGTTG GCTTCAGGGC TGCTAACCTG GATTCCTGTA CCATCCCTCA TGGTTCTCTC
Clustal ********** ** ******* ********** ********** ******** * **** ***** ********** ********** * *  ****   ********* ** ***  ** *  ******* ********** ********** **********

Human   CTACAAACCT TTGCAAAGAA TCCCTCCCCA CACGAGCAGA ATTTTAGTGC ATGCTCTCTG TGTTCTTCTA AGGTCCTCTT AGGGAATAGA CAATTCTGTA GGCACTTTCA TTGCAAACAG AATCCCAGTG AAGAAATGGT AGCGTCAGTC
Rhesus  CTACACACCT TTGCAAAGAA ACCC-----A TACTAGCAGA ATTTTAGTGC ATGCCCTCTG TGTTCTTCCA AGGCCCTCTT AGGGAATAGA CAATTCTGCA GGCACTTTCG TTGCAAACAG AATCTCAGTG AAGAAATGGT AGCATCAGTC
Clustal ***** **** **********  ***     *  ** ****** ********** **** ***** ******** * *** ****** ********** ******** * *********  ********** **** ***** ********** *** ******

Human   CAAGATACTA ATCAACATGG CAATCTTCAC TACAGGATGA GAACTGTTTT CCCTTTGCCC CAGGTAGGAC ATAGGCCGCC TTAG
Rhesus  CAAGATATTA GTCAACACAG CAATCTTCAC TACAGGATGA GAACTGTTTT CCTTTTGCCC CAGGTAGGAC ATAGGCTGCC TCAG
Clustal ******* **  ******  * ********** ********** ********** ** ******* ********** ****** *** * **
```

ENSG00000206110:

```
Human   ATGTTCTTCT CCATTCAGCT TCATGGAGAG ACTTTCCCAG GTTTCAGCCC ACGCTACGAA GCTAGACAGG AGAGTGGCCC CCACCCGCCC TGTTTGGAAC CCAGCTGGGG CATGCGGTGT TTGGAACACA TGGGCAGATT GGGGAGAGGC
Rhesus  ATGTTCTTCT CCATTCAGCT TCATGGAGAG GCTTTTCCGG GTTCCAGCCC ATGCTAGGAA GCTACACAGA AGAGTGGCCC C-ACCCGCCC TGTTTGGAAC CTAGCTGGGG CGTGCGGTGT TTGAAGCACG TGGGCAGATT GGGGTGAGGC
Clustal ********** ********** **********  **** ** * *** ****** * **** *** **** ****  ********** * ******** ********** * ******** * ******** *** * ***  ********** **** *****

Human   CAGAAGAGCC TCATCCAGAG CCAAGGACAA GCCTTGAGGC TGAAGAGCTC ACCAAGGCCA CTACAAGAGA GCAAAAAAAA GAGCCTCAGA GACTCCTCGA TAAATAACCA GAACAGAGAG CAAAGGTGGA GAGCAGGAGG GCAAGGCACT
Rhesus  CAGAAGAGCC TCTTCCAGAG CCAAGGATGA GCCTTGAGGC TGAAGAGCTC ACCGAGGCCA CTACAAGACA GAAAGAAAAA GAGCCTCAGA GACTCCTCAG TAAATAACCA GAACAGAGAG CACAGGTGGA GAGCAGGAGG GCGAGGCACT
Clustal ********** ** ******* *******  * ********** ********** *** ****** ******** * * ** ***** ********** ********   ********** ********** ** ******* ********** ** *******

Human   GAGACTTTAG AAGCTTCCAG GGAGAAGAAG AGTCAGATGC TTGAAGATGC CTCCTCAATG CTGCAAAGCT CTGAGGGAAA GTGGCTTTGA
Rhesus  GAGACTCCAC AAGCTTCCGG GGAGAAGGAG AGTCACATGT TTGAAGACGC CTCCTCGATG CTGCACAGCT CTGAGGGAAA GTGGCTTTGA
Clustal ******  *  ******** * ******* ** ***** ***  ******* ** ****** *** ***** **** ********** **********
```

ENSG00000206113:

```
Human   ATGGGGAGGT GCTCCTGGCA CCCAGAATGT GTGTCAGGCC AGGCTCTGGT GAAAGAA-GC CTTGGCCGGG ACCCGGGATA TGACATCCAC ACTGCGGTTC CATCCTCAGT CAACACAGAT GAGAAGGGTC AGCCCTGGAG CGCCCCCATG
Rhesus  ATAGGGAGGT GCTCCTGGCA CCCAGAATGT GTGTCAGGCC AGGCTCTGGT GAAAAAAAGC CTTGGCCAGA ACCCGGGATA TGACATCCAC GCTGAGGTTC CATCCTCAGT TAACACAGAC GAGAAGGGTC AGCCCTGGAG TGCCCCCATG
Clustal ** ******* ********** ********** ********** ********** **** ** ** ******* *  ********** **********  *** ***** **********  ********  ********** **********  *********

Human   CCCCACCCCC ACTTTGGGGG GCATCTTATC TCGGGAAATG GGTCCTCCAT CCCCCAGGCG CCCCAGGGCT GTGGCAGTGA GGGTGCGCAA GGCCACAACT TGTGCTGTAT TTGTGGTAAC CGAGACTTGG GAATCACTGA CTGGGTCTCC
Rhesus  CCCCACCCCC ACTTTGGAGG GCATCTTATC ACGGGAAATG GGTCCTCCAT CCCCCAGGGG CCCCAGGGCT GTGGCAGTGA GGGTGGGTGA GGTCACAGCT TGCGCTGTAT TTGTGGTAAC CGAGACTTGG GAATCACTGA CCGGGTCTCC
Clustal ********** ******* ** **********  ********* ********** ******** * ********** ********** ***** *  * ** **** ** ** ******* ********** ********** ********** * ********

Human   GACAGAGGCA GGGGCTGGCC TGGGCAGTGA GGCTCCCGGG GAGCCGAGGG CTGCAGGCTT CTGTACACAC CTCCTTCATC TTCCTACCCA CACCTGGAAG AAGTCACAGG CCCACCTGGA GGCTGCCCAA CTCCTCAGCT CCTGTCCTCC
Rhesus  GACAGAGGCA GGGGCTGGCC TGGGCAGTGA GGCCCCTGGG GAACCGAAGG CTGCAGGCAT CCATACACAT CTCCTTCATC TTCCTACCCA CACCTGGAAG AAGTCACAGG CCCACCTGGA GGCTGCCCAA CTCTTCAGCT CCTGTCCTCC
Clustal ********** ********** ********** *** ** *** ** **** ** ******** * *  ******  ********** ********** ********** ********** ********** ********** *** ****** **********

Human   CATCCCTGGG TTTTCCTCAC AGCTCCATCC TGGTCCTGGC ACCCCCTGCA ACCTCGGCAG CCCTGCCCCA CTCAGGGGCC TCCCTGTCAC CTGGTCTCAG CTCCCACCAC GAGGTGCTGA TCTCACATCC ACCACCTGTC AGTGGCATGT
Rhesus  TGTCCCTGGG TTTTCCTTAG AGCTCCATCC TGGTCCTGGC AACCCCTGCA ACCTTGGCAG CCCCACCCCA CTCAGGGGCC TCCCTGTCAC CCAGTCTTGG CTCCCACCCC CAGGTGCTGA TCTCACATCT GCCACCTGTC AGTGGCAGGT
Clustal   ******** ******* *  ********** ********** * ******** **** ***** ***  ***** ********** ********** *  ****  * ******** *  ********* *********   ********* ******* **

Human   GCCTGGGGAA CAGGAGCTTG GATTGCAGGG CCCCTGCCTC TAG
Rhesus  GCCCAGGGAA CAGGAGCTTA GATTGCAGGG CCCCTGCCTC CAG
Clustal ***  ***** *********  ********** **********  **
```

ENSG00000212693:

```
Human   ATGTATGACC ATGGGTATGA TACCTCCAGA AATGTTAACA ACTTATTCTT CTGCCATGAG TACCCCTCAT CAGGGTTGGT TTCAATGACA GGTTTTGGTG ATGTTCCTGA CCATATGAAG TGGTTTATGT TTAGAAACAT TCAAATTGAG
Rhesus  GTGTATGACC ATGGGTATGG TACCTCCAGA AATGTTAACA ACTTATTCTT CTGCCATGAG TACCCCTCAT CAGGGTTGGT TTCAGTAATA GGTTTTGGTG ATGCTCCTGG CCACATGAAG TGGTTTATAT TTGGAAACAT CCAAGTTGAG
Clustal  ********* *********  ********** ********** ********** ********** ********** ********** **** * * * ********** *** *****  *** ****** ******** * ** *******  *** *****

Human   GGACATCATT TACAGCATCG AGTGTGTCAG TTATACATGC ATTCATCATT AAATATCCTA GGTTTTATGA CAGACATTGA AATTATCACT CAGATGTGTT TAACAGCAAA AAATTCCCAG CACATCCTGG CAAAGGCTTT TATTTCCAAC
Rhesus  GGACATCATT TACAGCATCA AGTGTATCAC TGATACATGC CTTCATCATT AAATAGCCTA CGTTTTATGA CAAACACTGA AATTATCACT CAGATGTGTT TAACAGCAAA AA-TTCCCAG CACATCCTGG TAAATGCTTG TGTTTCCAAC
Clustal ********** *********  ***** ***  * ********  ********* ***** ****  ********* ** *** *** ********** ********** ********** ** ******* **********  *** ****  * ********

Human   CGTTGCATTC TTCATCTCTG CCTCCCATTG CCCACTGAAT GCTTTGCTTT CTGTGCATCA AGACAGAGTT CTAAAACCAG AAAACATCCA TCTTGA
Rhesus  CATTTTATTC TTCATCTCTG CTTCCCATTG CCCACTGAAT GCTTTGCTTT CTGTGCGTCA AGACGGAGTT CTAAAACCAG AAAACATCCA CCTTGA
Clustal * **  **** ********** * ******** ********** ********** ****** *** **** ***** ********** **********  *****
```

ENSG00000212736:

```
Human   ATGCTAAACT GGTTGGCCCA AATCCAACAG ATTGCAAAGA GTGGCAGGGC CTGGCAGCTG TCTGGAAGCC CTTCATCTTA CAGACAAGGA AGTGGTGTCA AATGGACAGT AAAGGTGAAT CACATACTCA GCTATAGCCT GCTCCCAGCT
Rhesus  ATGCTAAACT GGGTAGCCCA AATCCAACAA ATTGCAAAGA GTGGCAGGGC CTGGCAGCTG TCTGGAATCC CCTCATCTTA CAGACAAGAA AGTGGAGTCA AATGGAGATT GAAGGTGAAT CGCATACCCA GCTATAGCCT GCTCCCAGCT
Clustal ********** ** * ***** *********  ********** ********** ********** ******* ** * ******** ******** * ***** **** ****** * *  ********* * ***** ** ********** **********

Human   GGGGCACTCC AGGCCCCTCC TCTGGTCACT ACTGTAG-AA ATTAACTGCA TGTGTCTCAA TCTGCGAGTG CAGCTGCTTC TAGATGGTGG CACCTTCCAG AGCTTGGTTC GCCCTGTGTC TACCCAACAA GTACAGATGC CATCCCGGTG
Rhesus  GGGGTGCTCC AGGCCCCTCC TCTGGTCACT ACCATAGGAA ATTAGCTGCA TGTGTCTCAA TCTGCGAGTG CAGCTCCTTC TGGATGGTGA CACCTTCCCC AGCTTGGTTC ACCCTGTGGC TACCCAA--- GTCCAGACGC CATCCCGGTG
Clustal ****  **** ********** ********** **  *** ** **** ***** ********** ********** ***** **** * *******  ********   **********  ******* * *******    ** **** ** **********

Human   CTGTGATCTT CCAGCCATTT CTCCATTTCT GTCACAGCCC AGAAAGTGA
Rhesus  CTGTGATCTT CCAGCCACTT CTCCATTTCT GCCACAGCCC AGAAAGTGA
Clustal ********** ******* ** ********** * ******** *********
```

ENSG00000214112:

```
Human   ATGGAAAAGT GGAAACTTGG CAGACAGATC CAGCCTCCCT GGCCACTGGC CCATGCTCGT GGCTCCTGGA TGGCGCTGCC -ACGTTCTGA GCAGCTTGGG ACAGGTGGAG ATCAGGACTG GCAGCTGCAA GGACACACCA GAGCCACAGA
Rhesus  ATGGAAAAGT GGAAACTTGG CAGACAGATC CAGCCTCCCT GGCCATTGGC CCATGCTCGT GGCTCCTGGA TGGTGCTGCC CACGTTCTGA GCAGCTCGGG ACAGGTGGAG ATCAGGACTG GCAGCTGCAA GGACAGACCA GAGCCACAGA
Clustal ********** ********** ********** ********** ***** **** ********** ********** *** ******  ********* ****** *** ********** ********** ********** ***** **** **********

Human   AACTAAAGAG AATTTCCAAA AGGAGTCTAT GGTGAAGTCT CTGAGGATGC AAAGAAGACA AGGAGAATGA
Rhesus  AACTAAAGAG AATTTCCAAA AGGAGGCTAT GGTGAAGTCT CTGATGATGC AAAGAAAACA AGGAGAATGG
Clustal ********** ********** ***** **** ********** **** ***** ****** *** *********
```

ENSG00000214130：

```
Human   ATGGCCATGG CCCACGCAGG GCTCTGCGGG TGGAGAGGGG AAGTCAGCCT AGACTCCAGA GAGAAACCCT GGGGCAACCC GAAAGGCCTG AGGAAAACTG GAATTGGGGT TAGGCATGAG GGGAGGGAGT CTCTGGGGAA AACGGGCCGG
Rhesus  ATGGCCATGG CCCAAGCAGG GCTCAGAGGG TGGAGAGGGG A-GTCAACCT AGACTCCAGA GAGAAACCTT GGGACAACCC TAAAGGCCTT CTGAAAACTG GAACTGGG-T TAGGCACGAG GGGTGAGAGA CTCTGGGGAA AACCGGCCGG
Clustal ********** **** ***** **** * *** ********** * **** *** ********** ******** * *** ******  ********   ******** *** **** * ****** *** *** * ***  ********** *** ******

Human   GCTGTGCGGA GCCCTGAGGG CTCCTGCGGC TGCAGCGCTG CAGGCCGCCC GCGTCTCGCC CCGCCCCGCC GCTGGGCGTC CCCGCCAGGA GCCCACCCGC GGGCGGCAGC TTTTCTCCAT GCTGCCCAGG GAAGTTCGAT GCCTGGTGCT
Rhesus  GCT-TGCGGA GCCCTGAGGG CTCCTGCGGC TGCAGCGCTG CAGACCGCCC CTGTCTCGCC CCGCCCCGCC GCTGGGCGTC CCCGCCAGCA GCCCACCCGC GGGCGGCAGC TTTTCTCCAT CCTGCCCAGG GAAGTTCGAT GCCTGGTGCT
Clustal *** ****** ********** ********** ********** *** ******   ******** ********** ********** ******** * ********** ********** **********  ********* ********** **********

Human   GGGATGCGCC AGCGCTTCTG TTCGCTTCTG GCAATCCTGG CGTCTCCCAA TGAGAGGGCT CTCAAAATGA AGCTTTTAAT AAACTCCAGA GTAAGGAACT CGGGATTGTG CGGCAAAGGC CGCGCATTGC ACTTTGTGAG CAATCGGTAA
Rhesus  GGGATGCGCC AGCGCTTCTC TTCGCTTCTG ACAATCCTGC CGTTTCCGAT TGAGGGGGCT CTCAAAATGA AGCTTTTAAT AAACTCCAGA GTAATGAACT CGGGATTG-- CGGCAAAGGC CGCGCATTGC AATTTGTGAG CAATCGATAA
Clustal ********** *********  **********  ********  *** *** *  **** ***** ********** ********** ********** **** ***** ********   ********** ********** * ******** ****** ***
```

ENSG00000214780：

```
Human   ATGAACTCTG TGTTCAGACA GCCTGGCTCC AACTTCAGCT TCCCCATTTA TCAGCCGTCT GCCTCGGTAA CCCATGGCCT CAGTTTCTTC TGTGAAGTGA GCCCTCAAAT GCTTACCCCT CAAGATTATC GTGGGAAGCA A-CAAGGGCA
Rhesus  ATGAACACTG TGTTCAGACA GCCTGGCTCC AACTTCAGCT TTCCCATGTA CCAGCCATCT --------AA CCCATGGCCT CAGTTTC--C TGCGAAGTGA GCCCCCAAAT GCTTACCCCT CAAGATTATC GTGGGAAGTA AGCAAGGGCA
Clustal ****** *** ********** ********** ********** * ***** **  ***** ***         ** ********** *******  * ** ******* **** ***** ********** ********** ******** * * ********

Human   CAGAGGATGC CCTGGGTGCA TGGTGGCCAT GGACACATGG CCGGCCGTGC TTCCTGAGCT CCCTGGCTTT TGGGGAGAAG GGAGTGGAGG CCAGGCTTGG GGTGCCAGGG GTGAACCTTT GCTGCAGAAG GCAAACTGTT TGCTTGAGGC
Rhesus  CAGAGGAAGC CCTGGGTGCA TGGTGGCCAT GGACACATGG CCGGCCATGC TTCCTGAGCT CCCTGGCTTC TGCGGGGAAG GGAGTGGAGG CCAGGCTTGG GGTGCCAGGG GTGAACCTTT GCTGCAGAAG GCAAACTGTT TGCTTGAGGT
Clustal ******* ** ********** ********** ********** ****** *** ********** *********  ** ** **** ********** ********** ********** ********** ********** ********** *********

Human   TGTGAGGCTG TTCCTAAACT ACAGGCCCCT GGGGGCAGAG GGAGGAGAAC TAACATTTGG TAAGCGCCTG CTGGTGCCCA CTGCTGTATC ATGTGCCTAT GATTGCGTGA GCTCATCAGC CTTCCTGGGT ATCCAGCTCC TGGGAGACCC
Rhesus  GGTGAGGCTG TTCCTAAACT ACAGGCCCCT GGGGGCAGAG GGAGAAGAAC TAACATTTGG TGAGCGCCTG CTGGTGCCCG CTGCTGTATC ACGTGCCTAT GGTTGTGTGA GCTCATCAGC CTTCCCAGGT ATCCAGCTCC TGGGAGACCC
Clustal  ********* ********** ********** ********** **** ***** ********** * ******** *********  ********** * ******** * *** **** ********** *****  *** ********** **********

Human   CACAAGGAGC CCTGGCCACT GCTACCGTCA GGATTTTAAT GGCACACTGA GGCTCAGAGA GGGAAAGGGG CTTGTCCAGA GCCACACAGG GACTCTGTGG GCAGGGCCAC TCCAGGAAGG GACCCAGGGC CAGACCTGA
Rhesus  CACAAGGAGC CCTGGCCACT GCTCCTGTCA GGATTTTAAT GGCACACTGA GGCTCAGAGA GGGAAAGCGG CTTGTCCAGA GCCACACAGG GACTCTGTGG CCAGGGCCAC TCTAGGGAGG GACCCAGGGC CAGGCCTGG
Clustal ********** ********** *** * **** ********** ********** ********** ******* ** ********** ********** **********  ********* ** *** *** ********** *** *****
```

ENSG00000215071：

```
Human   ATGCAGATAT GCTTCTTAGC CTTGTGGGGT GGAACT---- ATGGCTGTAG CTACTTCTGC TGAAACTGCC CTTAGGACAT CATATGCATC TTTGAAAGCC AAATACATAA GTGAACACAT CTGCGTGTAT GTGTATACAC -CATACATGC
Rhesus  ATGCAGATAC GCTCCTTAGC CTCGTGGGGT GGAACTTACC ATGGCTGTAG CTACTGCTGC TGAAACTGCC CTTAGGACAT ---ATGAATC TTTGAAAGAC AAACACATAA GTGAATACAT CTGCATGTAT GTGTATACAC GCATACACGC
Clustal *********  *** ****** ** ******* ******     ********** ***** **** ********** **********    *** *** ******** * *** ****** ***** **** **** ***** **********  ****** **

Human   ACACATTCAT ACGCATGCAT ATACGTGTGC ATGTTTTAGT TAACAGAAGT CCTTGGGGGC TGGACAATAT TTTGGCTCCA AGGGATGTGA GCAACTATAA AGTCATGAGA CTGAGTTTCT TGTGCGGCTC CAGCGGCTCT GAAAGCCCCT
Rhesus  ACACATTCAT GCACATATGT ATACGTGTGC ACGTTTTAGT TAATGGAAGT CCTTGGGAGC TGGACAATGT TTTGGCTCCA AGGGATGTGA GCAATCATAA AGTCATGAGA CTGGGTTTCT TGTGCGGCCC CAGCAGCTCT GAGAGCCCCT
Clustal **********  * ***   * ********** * ******** ***  ***** ******* ** ******** * ********** ********** ****  **** ********** *** ****** ******** * **** ***** ** *******

Human   CTGAAGGGAG TCCACCAGCG CACAGTGTTA CATTCACCAG GCCAAGGGGT CTGCAGAGGT GCTGGTGTTG A
Rhesus  CTGAAGGGAG TCCACCAGCG CG--GTGTTA CA-------- -----AGGGT CTGCAGAGGT GCTGGTGTCG A
Clustal ********** ********** *   ****** **               **** ********** ******** * *
```

ENSG00000215458：

```
Human   ATGCCATGCA GACTTCTACA TCAAAGGGAA ACAAGGTCAG GAGGGCCGAG GGGTCCACGG CACTCCGCAC CCACGGGGCC AGGCTGGCAC AACGCCCCAA CGCTGCAATC CTGGGAAGAG TCACACGCGC CCTCCCGGGA CCCACGTGAC
Rhesus  ATGCCATGCA GCCTTCTATG TCAAAGGGAA ACGAGGTCGG GAGGACCGAG GGGTCCACAG CACTTCGCAC CCACGGGGCC AGGCTGGCAC A---CCCCAA CGCTGCAACC CTGGGAAGAG TCACACGTGC CCTCCCGGGA CCCACATGAC
Clustal ********** * ******   ********** ** ***** * **** ***** ******** * **** ***** ********** ********** *   ****** ******** * ********** ******* ** ********** ***** ****

Human   CATCAAGGGA G-----TGTG GAG--GACAC ATCCCTCGGG GGTGACGCCC CTGCAGATGG AGTTTCCCCA TCTGTGCCAC CCTTGCAAGG CCTGGGAAAG GCTGCGGGGC CAGGCGGCAC CGGGCAAGCT GAGTGCCAGG TGGTGGTTGC
Rhesus  TGTCGAGGGA GCCTGGTGTG GGGCAGACAC ATCTCTCAGG GGTGACGCCC CTGCAGATGG AGTTTCCCCA TCTGTGCCGC CCTTGTGAGG CCTGGAAAAG GCTGCAGGGC CAGGCGGCAC CGGGCAAGCT GAGTGCCAGG CAGTTGTTGC
Clustal ** ***** *     **** * *  ***** *** *** ** ********** ********** ********** ******** * *****   *** ***** **** ***** **** ********** ********** **********    ** *****

Human   AACACACCGG GCAAATCTGA AACCATGGG- ACGGACGAGC TGCACCCCTG GGAAAGAGAA CAGAGGAAGG CCGGTTATCA ACGTCCTCCT GTGCCTCAGT TTCCAGAAAC AAGCCAGACT CAGTGCCTCA GGGAGACCCC GTGTGGCCTC
Rhesus  AACATGCTGG GAAAATCTGA AACCCTGGGG ACGGACGAGC TGCACCCCTG GGAAAGAGGA CAGCGGGAGG CCGGTTATCA ACGTCCTCCT GTGCCTCAGT TTCCAGAAAT AAGTCAGACT CAGTGCCCCA GGGAGGCCCC GTGTGGCCTC
Clustal ****  * ** * ******** **** ****  ********** ********** ******** * *** ** *** ********** ********** ********** *********   *** ****** ******* ** ***** **** **********

Human   CGGAGAGCAC GGGACCCGCC CTTTGTGCAG GTGAGGAAAC TGAACCCCAG AGCAGTGAGG GACTGGCCTG GGGACCCTGG GCTCAGCCCT GGGCGCCTTC CCTCTGCCCA TCTCAGAC-- GGGCACAGCC AGCACCGCCT CACCCCAACG
Rhesus  TGGAGAGCGC AGGACCTGCC CTTTGTGCAG GTGAGGAAAC CAAACCCCAG AGCAGTGAGG GACTGGCCTG GAGACCCTGG -CTCAGCCCC AGGTGCCTTC CCTCTGCCCA TCTCAGACAG GGGCACAGCC AGCACCGCCT CACCCCA-CA
Clustal ******* *  ***** *** ********** **********   ******** ********** ********** * ********  ********   ** ****** ********** ********   ********** ********** ******* *

Human   CGCCTCCAGA CTGGCCCTCC AGGGGCCCCC AGGCACAATC CTCTCACTTT CTTCCTCAAG CCCCTGCCTT CCCCCTTCGC ACTGTGACCC AGGGGCTGCT AGCAGCTGGG CTGGGCTCCA AAGCTTGAAG CTGCTTCTCC AGG-----TC
Rhesus  CGCCTCCAGA TTGGCCCTC- -AGGGGCCTT GGGCACACTC CTCTCTCTCT CCTCCTCAAG CCCCCACCCT TCCCCTTCGC ACCATGACCC AGGGGCTGCT AGCAGCTGGG CGAGACTCCA AGACTTGAAG CTGCTTTTCC AAGCTGCCTC
Clustal **********  ********    *** **    ****** ** ***** ** * * ******** ****  ** *  ********* **  ****** ********** ********** *  * ***** *  ******** ****** *** * *    **

Human   TCTTTCCAGG GGGCGGCGGA TGGCTGCTCA CTCAGGGACG CCAACACCAA CAGGAAGGGA CCCATGCACG GATCTGACTT CCCTCCCAGG CTCTGCCTTC ACTCAATGTC CCTGTGGGGC AGCTGGGGCT CCCGGCCGCC CTCCCCTGCC
Rhesus  TCTTTCCAGG GGGCGGCAGG CGGCTGCTCA CTCAGGGACG CCGGCACCAA CAGGACGGGG CCCATGTGCA GGTCTGACTT TCCTCCCAGG CTCTGCCTTC ACCCAATGGC CCTGTGGGGC AGCTGGGGCT CCCGGCTGCC CTCCCCTGCC
Clustal ********** ******* *   ********* ********** **  ****** ***** ***  ****** *   * ********  ********* ********** ** ***** * ********** ********** ****** *** **********

Human   CACAGCCGTG AGGTTGCTTC CTAG
Rhesus  CACAGCCGAG AGGCTTCTTC CCAG
Clustal ******** * *** * **** * **
```

ENSG00000215494：

```
Human   ATGCCTTCCC AGCCGCACCT CAGTGCGTGT TCTGTTGAAT CTCCGGGTGC TGCAGCCAGG TTGACACAGC GACACCTCAC AGGTGTTGCT CGTGGAGGTG GGCAGACCGG TACATTCCAT ATGGGGTTAC TGCCCAGCAG ACAAGAGTGT
Rhesus  ATGCCTTCCC AGCCGCATCT CAGTTCATGT TCTGTTGAAT CTCTGGGTGC TGCGGCCAGG TTGACACAGC GACACCTCAC AGGC-TTGCT TATCGAGGTG GGCAGACTGG TACATTCCAT ACGGGGTTAC TGCCCAATAG ACAAGAGCGT
Clustal ********** ******* ** **** * *** ********** *** ****** *** ****** ********** ********** ***  *****   * ****** ******* ** ********** * ******** ******   ** ******* **

Human   GCTCCTGTCT CAGCCTTGAC CCTCACAACC ACCATGGGGC GGCTATTTAG TGTGCACTGG CAAATCTGGG GGCCCTGTTC TCCCTGCATC GTTTACCCCA AAGGTGATAG TGAACAATCT GCTTCCAAGT CGAAACAGGC TTTCCCATTG
Rhesus  GCTCCAGTCT CAGCCCCGTC CCTCGCAACC ACCGTGGGGC GGCTATTTAG TGTGCACTGG CAAATCCGGG G-CCGTGTTC TCCCTGCATC GTTTACCCAA AAGGTGACAG TGAACAATCT GCTTCCAAGT CGAGACAGGC TTTCCCATTG
Clustal ***** **** *****  * * **** ***** *** ****** ********** ********** ****** *** * ** ***** ********** ******** * ******* ** ********** ********** *** ****** **********

Human   CTATTGAGAA AGGGAATGGA ATGGCCTCTG TCCG------ -------CAT GGCTG----- GAATCC---- ---------- ---------- ---TCCTCCC CTGCCCCCTG CATGCCTCTC TGGAGACGCA ACTCATTCTG GGAATCCCGC
Rhesus  CTATGGAGAA AGGGAATGGA ATGGCCTCTG TCCGTGTGGT GGTCCTTCAT GGCTGACGAT GAGTCTGCCT GAGATGAGTC TGCGTGGCTG GAATCCTCTC CCGCCCCCTG CATGCCTCTC TGGAGACGCA ACTCGTTCTG GGAATCCTGC
Clustal **** ***** ********** ********** ****              *** *****      ** **                                 ***** * * ******** ********** ********** **** ***** ******* **

Human   CCGAGTCCTT GCAGGATTAG GGGGCTTTGC TCCATGAGAC CCCACGGGCT TTGA
Rhesus  CCAAGTCCTT GCTGGATTAG GGGGCTTTGC TCCGTGAGAC CCCAAGGGCT TTGA
Clustal ** ******* ** ******* ********** *** ****** **** ***** ****
```

ENSG00000215848：

```
Human   ATGAATGGCG GCCCCCAGAA GAGATGTAAG GTTGAGCACA GGAGCAGGGG TGGTAGAGAG GCCTCAGCAC TGGGCTTAGT TCCCAGACTA CTTAGAGGAC TGTGGGTAAT AAGAAAAGTA CTGGAGGAAT CAGAATGGGG GAATATCAGC
Rhesus  AGGAATGGCG GCCCCCAGAA AAGATGTAAG GTTGAGCACA GGAGCAGGGG TGGTAGAGAG GCCTCAGCAC GTGGCTTAGT TCCCAGAGGA CTTAGAGGAC TGTGAGTAAT AAGAAAAGTA CTGGAGGAAT CAGAATGGGG -AATATCAGC
Clustal * ******** **********  ********* ********** ********** ********** **********   ******** *******  * ********** **** ***** ********** ********** **********  *********

Human   ATGAGGAGAG AGAGAGTAAG ATGTGGCCAG GCAGTATCAT CTGCTAGTTT GAGGGTCTCT CTGGGGCCTG TGAAAGGTGA CTGTGTACTT GAGGTGATGC TCCACCTTCT CTCCTCAGCC CAGCACTCT- ---------- ----------
Rhesus  AGGAGGAGTG AGAGGGTAGG ATGTGGCCAG GCAGTATCAT TTGCCAGTTT GAGGGTCTCT CTGGGGCCTG TGAAAGGTGA CTGTGTACTT GAGGAAATGC TCCACCTTCT CTCCTCAGCC CAGCACTCTA GTTTCCATCT TTATAACTCT
Clustal * ****** * **** *** * ********** **********  *** ***** ********** ********** ********** ********** ****  **** ********** ********** *********

Human   GGTGAAACTG CATCCTCTCA GAGGCCACTC ACAGCCCCCT CAACAAAGAC CACTGGCCCT TCCCAAGCCC AGTCCTCCTT GACCTGTATG GAAAATGTTA TGCCATTTTC TCAGTCTTTT CCTTTGGCCT TCTGTTCTTC TGTTACATCC
Rhesus  GGTAAAACTG CACCCTTTCA GAGGCCACTC ATAGCCTCCT CAACAAAGAC CTCTGGCCCT TCCCAAGCCC AGTCCTCCTT GACCT--ATG GAAAATTTTA TGCTATTTTC TCAGTCTTTT CCTTTGACCT TCGGTTATTC TGTTATATCC
Clustal *** ****** ** *** *** ********** * **** *** ********** * ******** ********** ********** *****  *** ****** *** *** ****** ********** ****** *** ** *** *** ***** ****

Human   ATGATCATCT GTTCTTGGTT CCATTCATTC AGTCCTCCGG ATGGACCAGT TCCTTAA
Rhesus  GTGATCATCT GTTCTTGGTT CCATTCATTC AGTCCTCCAG ATGGACCAGT TCCTGGT
Clustal  ********* ********** ********** ******** * ********** ****
```

ENSG00000218478：

```
Human    ATGTACTTTT GTGGCCTTGG TGTCCGATGG GGCTGGGGGA GAGTGCTCTC CACTGACCCA GCAGCACACC CATGTGCAGT GCGCCTGCAT CTGTGTGGGG GCAGCCACAC CCCTTGGCTG CTGCTTCCTT GGGCTGCCTT TCTGGGGGCA
Rhesus   ATGTACTTTT GTGGCCTTGG TGTCCAACGG GGCTGGGGGA GAGTGCTCTC CACTGACCCA GCGGCACACC GATGTGCAGT GCGCATGCGT CTGTTTGGGG GCAGCCACAC CCCTCGGCTG CTGCTTCCTT GGGCTGCCTT TCTAGGGGCA
Clustal  ********** ********** ***** * ** ********** ********** ********** ** *******  ********* **** *** * **** ***** ********** **** ***** ********** ********** *** ******

Human    TGTGACTGGA CCTACGAGGT CTGCACTGAG CTCCATTTGA ATGATACCTT TCCTATCCCA TTTCCCCCAC GGAAGCACCG CTTCAGGGTT ATTCAGTCCT CTGCCTCATG GCTGAAATTG CTCATCTCGT CTGCAGATGT CTACTATCCT
Rhesus   TGTGCCTGGA CCTACGAGGT CTGCACTGAG CTCCATTTGA ATGATACCTT CCCCATCCCG TTTCCCC-AT GGAAGCACCG CTTCAGGGTT ATTCAGTCCT CTGCCTCATG GCTGAAACTG CTCATCTCGT CTGCAGATGT CTACTATCCT
Clustal  **** ***** ********** ********** ********** **********  ** *****  ******* *  ********** ********** ********** ********** ******* ** ********** ********** **********

Human    GTCTACCTAA TGCACTATTA TGTATTGATT CTCCATGAGA CAGAGAGAGA GAGAGACTAT CAGATAGTTT ACACCCAAAG GGTAGGTTTT TGTATATTTT TCCAGCCTTT TTTATTAAGG GGAAGGGGAG AGTTTAAAAA CCCAAACCGT
Rhesus   GTCTACCTAA TGCACTATTA TGTATTGATT CTCCATGAGA CAGAGAGAG- ---AGACTAT CAGATAGTTT ACACCCAAAG GGTAGGTTTT TGTATATTTT TCCAGCCTTT TTTATTAAGG GGAAGGGGAG AGTTTAAAAG CCCAAACCGT
Clustal  ********** ********** ********** ********** *********     ******* ********** ********** ********** ********** ********** ********** ********** *********  **********

Human    TGTGGTTTTA AGGTGTTTCA TTTTTAA
Rhesus   TGTGGTTTTA AGGTGTTTCA TTTTTAA
Clustal  ********** ********** *******
```

ENSG00000221891：

```
Human    ATGGAAGGAT GCGCGGTGCG GCGGGGCAGC TGTCCTCTTC TCCCCGGACC CAGCGCCTGG AGAGCCAGCC CTGCAGGGTG GGCTGGGCGA GCCAAACTGC GTTCCTGGTG CAGGGCTTCG GGTCTCCCTA ACAGACCTTA TACGCTGACC
Rhesus   ATGGAAGGAT GCGTAGTGCG GCGTGGCAGC TGCCCTCTTC TCCCCGGACC CAGCGCCTGG AGAGGCAGCC CTGCAGGGTG GGCTGGGCGA ACTAAACTGC GTTCCTGGTG CAGGGCTTCG GGTCTCCCTA ACATACCTTA AACGTTGAAC
Clustal  ********** ***  ***** *** ****** ** ******* ********** ********** **** ***** ********** **********  * ******* ********** ********** ********** *** ******  *** *** *

Human    GGCGGCCGCC ATGGCAGTGT CTCTTTGCTC AGACATCCAG GGACGACCAC ATTCGTCCAA CAGCGGTCGC TCCACCAATC CTGGGAGAAG CGAATCGTTT TCTCCGCGTG CCCTGTCAGC CGCTCATGGT GCCCAGAGAG GAA----TTT
Rhesus   AGCCGCCGCC TTGGCAGTGT CTCTTTGCTC AGAGATCCTG AGACGACCAC ACTCGTCCAA CAGCAGTCGC TCCACCAAGC CTGGGGAAAG CGAATCGTTT TCTCCGCGTG CCCTGTCAGC CGCTCATGCT GCCCAGAGAG GAAGGAATTT
Clustal   ** ******  ********* ********** *** **** *  ********* * ******** **** ***** ******** * *****  *** ********** ********** ********** ******** * ********** ***    ***

Human    TAGTGGCAGC ATTCCGGCTG TCACGCCACC GAAATTGCCA GGCCACTCCA AGTCAGAAGG ACCACCAGGA AAAGTCAGGA AGAGAACCAC CATCAGGTCC CAGCCTCTTT TTGTGACAAG GACTAGAGGG TTTG---GGT CTGCAGTGGG
Rhesus   TAGTGGCAAC ATTCCGGCTG TCACGGCACT GAAATTGCCA GGCCACTCCA AGTCAGAAGG ACCACCAGGA AAAGTCAGGA AGAGAATCAC CATCAGGTTG CAGCCTCTTT TTGTGACAAG GACTAGATGG TTGGTTTGGT CTGCAGTGGG
Clustal  ******** * ********** ***** ***  ********** ********** ********** ********** ********** ****** *** ********   ********** ********** ******* ** ** *   *** **********

Human    CTGGCT-CCC GCTGGGCTCA CCTGTCCTAT AG
Rhesus   CTGTCTTCCC GCTGTACTGG CCTCTCCTGT AG
Clustal  *** ** *** ****  **   *** **** * **
```

ENSG00000221899：

```
Human    ATGAATACGT ACACCCGCAG CGCCTCCTTC ACTCCGAAGA CTTTCACTTT CCCCCTTGCA CAAAACCGAA CATCCCACCC CACCACCACC TCCAAGCTAA AATACCTGGG CCGCCCCTCC CCACGAAGGG ACGCGCTCCA GGCGAATGGG
Rhesus   ATGAATATGT ATATCCGCAG CGCCTCCTTC ACTCCGAAGA CTTTCACTTT CCCCCTTGCA CAAAACCGAA CATCCCACCC CACCCCCACC TCCAAGCTAA AATACCTGGG CCACCCCTCC CCACGAAGGG ACGCGCTCCA GGCGAATGGG
Clustal  ******* ** * * ****** ********** ********** ********** ********** ********** ********** **** ***** ********** ********** ** ******* ********** ********** **********

Human    GAGGGCCCGG AAGTCGGGCC TGGGACAGAG GAGCCGGCCC ACCGCCTCCC GGTCGGAAGC GGCAGTGCAG GTGAGGCGGC GGCCAGCCTG GGGACTGCGC GGGTAAGGAG CGGGAGGGCG GAATGCACCG CGAAGTTGGC GGGGGACGGC
Rhesus   GAGGGCCCGG AAGTCGGGCC CGGGACGGAG GAGCCGGCCC ACCGCCTCCC GGTCGGAAGC GGCAGTGCAG GTGAGGCCGC GGCCAGCCTC GGGACTGCGC GGGTAAGGAG CGGGAGGGCC GACTGCACCG CGAAGTTGGC GGGGGACGGC
Clustal  ********** **********  ***** *** ********** ********** ********** ********** ******* ** *********  ********** ********** *********  ** ******* ********** **********

Human    CACGCACGCC TCGCCTCGGC CTTCCTGCAC CGTAGGG--- --CCGCCCTC CCGCCACCCC TGGGCACCTG TCGACCACGT CTGGGCGAGG GCGGGTCGGC TCCCGCCCTC GGGCCCTCAC C--------- ------GCGG CGGGGACGCC
Rhesus   CGCGCACGCC TCGTCTCGGA CTTCCTGCCC CGTAGGGGCT GGCCGCCCTC CCGCCACCCC AGGGCACCTG CCGACCACGT CTGGGCGGGG GCGGGCCGGC TCCCGCCCTC GGGCCCTCAC CCCACCCCGG CCAGCCGCGG CGGGGACGTC
Clustal  * ******** *** *****  ******** * *******      ******** **********  *********  ********* ******* ** ***** **** ********** ********** *                **** ******** *

Human    GGGTCAGCGA AGGGTTAAGG GACTCGCTCC CTCCTCTCGG CTCCCTCACC CTTGGAAAGT CCCCGAAATG A
Rhesus   GGGTCAGCGA AGGGTTAAGG GACTTGCTCC CTCCTCTCGG CTCCCTCACC CTTGGAAAGT CCCCGAAATG A
Clustal  ********** ********** **** ***** ********** ********** ********** ********** *
```

ENSG00000221953：

```
Human    ATGGGCCTGT GCACCCTCCA GCCCCTGGGC CCTCCTCGAA AATCCTCCAC ATCCTGCGGG ACCTGGACCG CCAGCGGCCT CCCAAGCCTG GGCCATTTGC CCCGTCGCCT CCGCCTGGCA TTCGACTTCT TAGAACCGCG GGCACGAGGG
Rhesus   GTGGGCCTGT GCACCCTGCA GCCCCTGGGC CCTCCTGGAA AATCCTTCGC ATCCTGTGGG ACCTGGACCG CCAGCGGCCT CCCAACCCTG GGC-ATCTGC CCC-TCGCCT CCGCCTGGCA TTGGACTTCT GAGAACCCTG GGCACGAGGG
Clustal   ********* ******* ** ********** ****** *** ****** * * ****** *** ********** ********** ***** **** *** ** *** *** ****** ********** ** *******  ******  * **********

Human    CAGCGTGGGG GCAGTGGTGG CTGTCAGTCC ACGCGGGCCG CCGAGAGGAC GCGCCCACCC CACCCTCCAA ACGCCGTCCT CCTGCTTCAG CCAGAGCCGT CCCTGACATG GGCCCAGGGA CGCG--GCTG CAGAGGCCAC TTCCGG----
Rhesus   CAGCGTGGGC GCAGGGGAGG CTGTCAGTCC AGGCCGGC-G CTGAGAGGAC GCGTCCACCC CACCCTCCAA ACGCCGTCCT GCTGCTTCAG CCAAAGCCGT CCTGGAGACG GGCCCAGGGA CTCCCCGCTG CAGCCTCGAG CTCCGGGGTG
Clustal  *********  **** ** ** ********** * ** *** * * ******** *** ****** ********** **********  ********* *** ****** **  ** * * ********** * *   **** ***   * *   *****

Human    ------TCAC TCCCCGCTGC GGCCTCGCG- CTCCGGGAGC CGCACCCTCC GCTGCGCCAG CTC----CGA CCGCAGTCTT CGGGAGCAGA AGCAGCGCCG CGCGGGCCCT GACCCCACAC CCT-CCCCGG CGCCTCCGCC TGCAGGACCC
Rhesus   GCCCCGCGGC TCCCAGGGGT GGCCCCGCGG CTCCAGGAGT CACACACTCC GCCGCGCCAG CTCGGACCGA CCGCAGACTT CGCGAGCAGA AGCAGCGCCG CGCGGGCCCT GACCCCACAC CCTGCCCCGG CGCCTCCGCC TGCAGGACCC
Clustal           * **** *  *  **** ****  **** ****  * *** **** ** ******* ***    *** ****** *** ** ******* ********** ********** ********** *** ****** ********** **********

Human     CGGCCCTCCC CGGGCTCGCT GGGTCCCTCC GCCCCGGCCG CTCCCCGCAC CGCGCGCGGT GCCTACGAGC TCCAAGGCGG CGCCAGCCAA GACGGTCCCG GCCAAGCGGC GGTGGGTGCG AC-------- ---------- ----------
Rhesus    ------TCCC CGGGCTCGCT GGGTCCTTCC TCCCCCGCCG CTCCCCCGAC CGCGCGCGGT GCCGACG-GC TCCAAGGCGG CGCCAGCCAA GACGGTCCCG GCCACGCAGC GGTGGGTGCG GCGGGTGCGG CGGGTGCGGC GGGTGCGGCG
Clustal         **** ********** ****** ***  **** **** ******  ** ********** *** *** ** ********** ********** ********** **** ** ** **********  *

Human     --------CC CCACCACGGG CCCCGGCACC GGCGGGGAGG GCGCGCTCCT AGGCTGCGGG AGCGGACGGA CCCCTCCGAC TTCGGCCAC- ---CTGGCGC CG-------- ---------- -CCGCCTCCT CCCCGCCGAA GTCCCTCCCG
Rhesus    GGTGCGGCCC GCACCACGGG CCC-GGCA-- GGCGGGGAGG ACGCGCTCCC AGTCTCCGGC AGCAGACAGA CCCCTCCGAC TCCGACTCCG ACTCCGACTC CGACTCCGAC TCCGACTCCG GCCGCCTCCT CCCCGCCGAG GTCCCTCCGG
Clustal           **  ********* *** ****   **********  ********  ** ** ***  *** *** ** ********** * ** *  *     * * * * **                      ********* *********  ******** *

Human     GGGCAGCCGC CGCAAACTTT CCGGAGCGGG AGCGACTGTG A
Rhesus    GGGCGGCCGC CGCACACTTT CCGGAGCGGG AGCGACTGTG A
Clustal   **** ***** **** ***** ********** ********** *
```

ENSG00000221990：

```
Human    ATGCCTGCTG TCTTTATGTT GGCCTCGTCG TCCGCACTGC AGTGTGGCAG GGGCGTCCCT CGTTTCCCGC GGACTGAGGT GGGCGCCGGG CATTCAGTAA ACGAAGAAAC CAAAGCGGAG AAGGTTGGGA ATCAAACGTC TGTCATACCT
Rhesus   ACGCCTCATG TCTTTATGTT GGCCTTGCCG TCCGCACTGC AGTGTGGCAG GGGCGTCCCA CCTTTGCCGC GGACTGAGGT GGGCGCCGAG CATTCAGTAA ACGAGGAAAC GAAAGCGGGG AAAGTTGGGA ATCAAGCGTC TGTC-TGCCT
Clustal  * ****  ** ********** ***** * ** ********** ********** *********  * *** **** ********** ******** * ********** **** *****  ******* * ** ******* ***** **** **** * ***

Human    GCCACCAGCA GACAAGCGGC TTTGGGTACC TCCTGGACCC AAAGACGAAC TCAGCCCCTC CAGGAGCGCA GTCATTGGCA CCCTCGTGGG AACAATGCCA GTGGGATGGG TGGCCACCGG ATGTTTCCGG GGCCCCTCAG AGGCCCAGCA
Rhesus   GCCACCAGCA GACTAGCGGC TTTGGGTACC CCTTGGACCC AAACACGAAC TCAGCCCCTG CAGGAGCACA GTCATCGGCA CCCACGTGGG AACAGTGCCC -TGGGATGGG TGGCCACGGG ATGTTTCTGG GGCCCCTCAG AGGCCCAGCG
Clustal  ********** *** ****** **********  * ******* *** ****** *********  ******* ** ***** **** *** ****** **** ****  ********* ******* ** ******* ** ********** *********

Human    GCGCAGGTCT TGGAGAATGA ATGTGGGTCA CTGGGCCGC- GCTGCAGAGG GCCGGTCCTG A
Rhesus   GCGCGGGTCT TGGAGAATGA ATGTGTGTCA CTGGGCCGCT GCTGCAGAGG GCCGGTCCTG A
Clustal  **** ***** ********** ***** **** *********  ********** ********** *
```

ENSG00000223857:

```
Human     ATGTCTGG CGGACACAGC TCCCCGGAAC CTCCACGCCC ATGGCCACTA GACAGAGGGA GTCTTCCTTC ACCTCCTGCT TTTCCACCTG GAATTGCGAC GCGGGCGACG AGGGCGTGGG CTGCACCTGC GAAGATGCTT CCTTGTGCAA
Rhesus    GTCCCTGG CGGACACATC GCCCTGGGAC CTCCACGCCC ATGGCCACTA GGCAGAGGGA GTGTTCCTTC ACCTCCTGCT CTTACACCTC GAGCTGCAAC GCGGACGACG AGGGCATGCG CAGCACCTGC GAAGATGCTT CCCTGTGCAA
Clustal    *  **** ******** *  *** ** ** ********** ********** * ******** ** ******* **********  ** *****  **  *** ** **** ***** ***** ** * * ******** ********** ** *******

Human     GAAGCGCCTG TCGGGCGCGG GATTCGGGGC TGGCATCTGG GACGCGGGCT GAGGTGGGAG GCGGGCCTGC ATCTGAAGAA TACGCTCGGA GGCTGGCAGG TTGCTGCCCC CGCCTCGCAC GACCCTCGCT TCCCACCTGT GAAATGCACA
Rhesus    GAAGTGACTG CCAGGCGCGG AGTTCGGGGC CGGCATCGGG AGCACGGGCC TAGGTGGGAG GCGGGCCTGC GTCTGAAGAA TGCGCTTGGA GGCTGGCAGG TTGCAGCTCC CGCCTCGCAC GACCCTCGCT TCCCAACTGT GAAATGCACA
Clustal   **** * ***  * *******   ********  ****** **   * *****   ********* ********** ********* * **** *** ********** **** ** ** ********** ********** ***** **** **********

Human     GAACAGGGCT TCATTTATTT AACGAATCGT TTCTGAGCTC CTGCTGTGAG CCAGGCTTGG AGCAAGCCTG GGTACTGTGG ATGGGAGCAG GCCCCTGA
Rhesus    GAACAGGGCT TCATTTATTT AACGAATCGT TTCTGAGCTC CTGCTGTGAG CCGGGCATGG AACAAGCGCC TGAACCAGGG AGACGGGGTC TTGGCGTT
Clustal   ********** ********** ********** ********** ********** ** *** *** * *****     * **   ** *   * *        *
```

ENSG00000224013：

```
Human    ATGATTGGGA AGGTGGAAGG TGGAGACGCG GTCCTTA--- --GGTGGTCG AGCGCATTTT CTCTTCCTCA -GAGTCCAGG AAGGAGCGCG ATGGAGAAGT GGGGCGCACG GCGGGTTTCG GGTCCGCCCC TGCCCTTCCG ACTCCTCCGA
Rhesus   CAGATTGAGA AGGTGGAAGG TGGAGACGCG GGCCTTAACT TAGGTGGTCG AGCGCACTTT CTCTTCCTCA AGAGTCCAGG AAGGAGCGCG ATGGAGAAGT GGGGCTCACG GCGGGTTTCG GGTCCGCCCC TGCCCTTCTG ATTCCGGCGA
Clustal    ***** ** ********** ********** * *****      ******** ****** *** **********  ********* ********** ********** ***** **** ********** ********** ******** * * ***  ***

Human    CCACGTCTGC CTTTGGCTGA AAGCAAATAT GGCTCCGTGT GCGGTTTCTG CAACAAAGGC TGGGCGGGCC GGGTTTCCCT AGGGATGGGG ACCGCCTCCC CGGGGAGTCG TGGGGGTGAG CCACCGGGGC CTCCGAGAGA ATCGCTGGTG
Rhesus   CCACGTCTGC CTTTGGCTGA AAGCAAATAT GGCTCCGAGT GCAGTTTCTA CAACAAAGGC TGGGCGGGCC GGGTTTCCCT AGGGATGGGG ACCGCCTCCC CTGGGGGTCT TGGGAGTGAG CCACCGGGGC TTCCGAGAGA ATCGCTGGAG
Clustal  ********** ********** ********** ******* ** ** ******  ********** ********** ********** ********** ********** * *** ***  **** ***** **********  ********* ******** *

Human    TCGCTTCGCA CCCAAGGGAC ACATCTCGGG TTAGAAAGGA GAAACGACGT GGATCTAAAG GCGAAGCCCA TGCTGAGGAC TTTTCAAAAT GGCCTCTTAT CGGCCCCACT CACAGGAGCT TCAGGCTTCG GTGGCCCTGT AGGACAACCC
Rhesus   TCGCTTCGCA CCCAAGGGAC ACATCTCGGG TTAGAAAGGA GAAGCGACGT GGATCTAAAG GCGAAGCCCA TGCTGAGGCC TTTTCAAAAT GGCCTCTTAC CAGCCACACT CACAGGAGCT TCAGGCTTCG GCGGCCCTGT AGGACAACCC
Clustal  ********** ********** ********** ********** *** ****** ********** ********** ******** * ********** *********  * *** **** ********** ********** * ******** **********

Human    TGGGGTGTCA TTGGAGAGAA GCTGACGGGG TTAATGGCGG GTGACAGGTG A
Rhesus   CGGGGTGTCA TTGGAGAAAA GCTGACGGGG TTGATGGCGG GTGACAGGTG A
Clustal   ********* ******* ** ********** ** ******* ********** *
```

ENSG00000224376：

```
Human    ATGCCTGCCA TCCCCAAACA TGGGCTCAAA ACCCCCTTCC CAGGGACGGC TGTGTCCACC CCTCAGCAGG AGGAGGAGAC CCCACAGCAC CGGGTGAGAG GATTAAGTGA GCCAGTCATG CATGGGCCGA GAACCAGGTT TGGCATATTG
Rhesus   ATGCCTGCCA TCCCCAAACA TGGGCTCAAA ACCCC-TTCC CAGGGACAGC TGTGTCCACC CCTCAGCAGG AGGAGGAGAC CCCGCAGCAC CAGGTGAGAG GATTAAGTGA ACCAGTCGTT CATGGGCGGA GAACCAGGTT TGGCATATTG
Clustal  ********** ********** ********** ***** **** ******* ** ********** ********** ********** *** ****** * ******** **********  ****** *  ******* ** ********** **********

Human    TAG
Rhesus   TAG
Clustal  ***
```

ENSG00000225021：

```
Human    ATGAACTCGT TGACCGTCAT GAGGGTCTGG GACCATTCCT CATCTGTGTA CCTCGAACCA ACTTCCACTG GGACAGTTCG GCAGCCAGCG ATCTCCTGGA TATACTCCAA ACTAGCAGAA AGACACACAT TGGCATCGTC ACTGCTAATA
Rhesus   ATGAACTCGT TGACCGTCAT GAGTGTCTGG GACCATTCTT CGTCTGTGTA CCTCGAACCA ACTTCCACCG GGACGGTTCG GCAGCCAGCG ATCCCTTGGA TATACTCCAG ACTAGCAGAA AGACACACAT TGGCGTCATC ACTGCTAATA
Clustal  ********** ********** *** ****** ******** * * ******** ********** ******** * **** ***** ********** *** * **** *********  ********** ********** **** ** ** **********

Human    TCTGTTGGGC ATAGTACTAA GTGTGCTTTG CATGTGGTCC CCTTTCATCC TCGGAAAACC TCACGTGCAG AGACAAGGAC TGTCATCCCC AGCTTCACTG ATGAGGAACC GG-CATGGAG AGGTGGGGCT CCCTGCTCAA GGCTGTGTGG
Rhesus   TTTTTTGGGC ATAGTACTAA GTGTGCTTTG CATGTGGTCC CCTTTCATCC TCGGAAAATC TCATGTGCAG AGACGAGGAC TGTCATCCCC AGCTTCACTG ATGAGGAAAG GGGCACGGAG AGGCGGGG-- -CCTGCTCAA GGCTGTGCAG
Clustal  * * ****** ********** ********** ********** ********** ******** * *** ****** **** ***** ********** ********** ********   ** ** **** *** ****     ********* *******  *

Human    CTCATACACA GTGCTGTGCA GCTTTGCAGA CAGCGCCGGT AAGTCTGGTT GGCTGGGCTG CTCTAGTTCC CTGCTTGCCA ACAGCCTCCT CACTTCTTTC TTTGCTCATT TTGTCACTAA GCTGAGAAAC CTTTGA
Rhesus   CTCCTACGCA GTGCTGCAGA ACTCTGCAGA TAGCGCCGGT AAGTCTGGGT GGCTGGGCTG CTCTGGTTCC CTGCTTGCCA GCAGTCTCCT CACTTCCTTC TTTGCTCATT TTGTCACTCA GCTGAGAAGC CTTTGA
Clustal  *** *** ** ******   *  ** ******  ********* ******** * ********** **** ***** **********  *** ***** ****** *** ********** ******** * ******** * ******
```

ENSG00000225860：

```
Human    ATGCCTCAGT GGCTGGTGGC GTCCATCATT CCTGGGTGTG TCTGTGAGAG TGTTTCCAGA GGAGACTCAC ATGTGAGCCA GCGGGCTGAG AAGGAGACCC GTTCTCACTG TGACTGTGCA CTGTCCAATC AGCTCAAGGC CAGGCTGGGG
Rhesus   ATGCCTCAAT GGCTGGTGGA GTCCATCATT CCTGGGTGTG TCTGTGAGAG TGTTTCCAGA GGAGATTCAC ATGTGAGTGA GCGGGCTGAG AGGGAGACCC GTTCTCACT- -GACTGGGCA CCATCCAATC AGCTCAAGGC CAGGCTGGG-
Clustal  ******** * *********  ********** ********** ********** ********** ***** **** *******  * ********** * ******** *********   ***** *** *  ******* ********** *********

Human    ACAAACAGGC AGAAGAAGGA GGATTCTCTC TCCCACCTTT CTGGAGCAGG ATGCCTTTTC TCCTTGGACA TCAGACTACA GGGTCTTTGG CTTTTGGATT CTAGGACTTG TACCAATGGC CTCCCGGGGC CCTCAGGCCT TCAGCCTCCA
Rhesus   ACAAACAGGC AGAAGAAGGA GGATTCTCTC TCTCCGCTTT CTGGAGCAGG ATGCCTTTTC TCCTTGGGCA TGAGACTACA GGGTCTTTGG CTTTTGGATT CTAGGACTTG TACCAATGGC CTCCCGGGGC CCTCAGGCCT TCAGTCTCCA
Clustal  ********** ********** ********** ** *  **** ********** ********** ******* ** * ******** ********** ********** ********** ********** ********** ********** **** *****

Human    ACGAAGGTCT GTGCTGTTGG CC-TCCCTGA TTCTGAGGCT TCTGGACTTG GACTGAGGCA TGCTACGGGC TTCTCTGATT CTCCAGCTTG TGGATGGCCT ATCATGGGAC TTCTCCACCT CTGTAATCAC AAGGGCCAAT GCCCCCTAAT
Rhesus   GCAGGGGTCT GTGTTGTTGG CCCTCCCTGA TTCTGAGGCT TCTGGACTTG GGCTGAGGCA TGCTACTGGC TTCTCTGATT CTCCAGCTTG TGGATGGCCT ATTATGGGAC TTCTCCGCCT CTGTAATCAC ATGGGCCAAT GCCCCCTAAT
Clustal   *   ***** *** ****** ** ******* ********** ********** * ******** ****** *** ********** ********** ********** ** ******* ****** *** ********** * ******** **********

Human    ACCTTTCTTT TCATATATCC TACTGGTTCT GTCTGCCTGG GGAACCTTGA CTAATACAGA TATGGAGCAT TTGAAATGA
Rhesus   GCATTTCTTT TCATACATCC TACTGGTTCT GTCTGCCTGG GGAACCTTGA CTAGTACAGA TACGGAGCAT TTGAAATGA
Clustal   * ******* ***** **** ********** ********** ********** *** ****** ** ******* *********
```

ENSG00000225917：|

```
Human        ATGCAGCCGG GCTGCGCGGT CCCGCAGAGC GGGCGCCTCC GGGGCTCGAG TCGGGGACCC GGCAGGAGCG GGCCGGGGAT GGCGGCGGCG GGCGGCGGGT CGGCCGTGGA GCCCCGGAGA GGCGGCCGCC ACCACCGCTG CCCGCAGTCT
Rhesus       ATGCAGCCGG GCTGCGCGGT CGCGCAGAGC GGGCGCCTTC GGGGCTTGGG TCTGGGACCC GGCAGGAGCG GGCCGGGGAT GGCAGCGGCG GGCGGCGGGT CGGCCGTGGA GCCCCGGGGA GGCTGCCGCC CCGGCCGCTG TCCGCAGTCT
Clustal Co   ********** ********** * ******** ******** * ****** * * ** ******* ********** ********** *** ****** ********** ********** ******* ** *** ******  *  ******  *********

Human        GCGAGGTGTC CGGCGGTCCG CTCAGGGCCT CTTTTGCAGA CTCGGTGCCC GGAGTGCGCC GGCGCCGCCC GCCAGGTCTT GGCACTGCTG GCGCCG---- --------CC GCGGCGGCGG GGGCCCGGCT CCAGATCACC GACCCCCACG
Rhesus       GCGAGGTGTC CGGCCGCACG CTCAGGGCCC CTTTTGCAGA CTGGGTGCCC GGAGTGCGCC GGCGCCGCCC GCCAGGTCTT GGCACCGCTG GCGCCGGCGC GGGCGCGGCC GCGGCGGCGA GGGCCCGGCT CCAGATCCCC GACCCCCGTG
Clustal Co   ********** **** *  ** *********  ********** ** ******* ********** ********** ********** ***** **** ******             ** *********  ********** ******* ** *******  *

Human        AGCGCCGGCC CTGCCTGCCC GCTATTTTGG TTCCATCTCT TCTGCATATT TATGCTTGCC TGTTACATTC CCTGGCCTCT GAGTGTCCTT TGGCAGTTTC CAAAGCATTT GGGCAAGAAG GAGCTTGGGC AGCTTCACAA CAGGTGGGGA
Rhesus       AGCTCCGGCC CTGCGTGCCC GCTATTTTGG TTCCATCTCT TCTGCATATT TATGCTTGCC TGTCACATTC CCTGGCCTCT GAGTGTCCTT TGGCAGTTTC CAAAGCACTT GGGCTAGAAG GAGCTTGGGG AGCTTCACAA CAGGTGGGGG
Clustal Co   *** ****** **** ***** ********** ********** ********** ********** *** ****** ********** ********** ********** ******* ** **** ***** *********  ********** *********

Human        CGCGCAGGGG AGGGGCCGCG GCCGGACCGG CAGAGT--TT CAAGGGCTGG CGCGCCGGCG GACTTTCCAC ACTGTCTTTC GGGACTGGGG TTAGGAGACA GCCCTTGGCC ACCACTGTGT CCTTACTTTG TCGCTTTACT TGGGGCTGGA
Rhesus       CGCGCAGGGG AGGGGCCGCG GCCGGACCAG CAGAGCGCTT CAAGGGCTGG CGCTGGGACG GACTTTCCAC ACTGTCTTTA GGGACTGGGG TTAGCAGACA GCCCTTGGCC ACCACTGTGT CCTTACTCTG TCGCTTTACT TGGGGTTGGA
Clustal Co   ********** ********** ******** * *****   ** ********** ***   * ** ********** *********  ********** **** ***** ********** ********** ******* ** ********** ***** ****

Human        CCTCCCCTTT CCAGCTCTGG CGTGTGACCC CGCAACGAGC GTGGTGTCGA GACCACCACC AGCTGGGGCG GTGGGAGCGA AGGTGCCCCA GGCTCGGGGA AGGTGGAAAC CTTTTCCCCG CTCCCCAAGT GATTGTTGCG CGCTCCGGTG
Rhesus       CCTCCCCTTT CCAGCTCTGG CGTGTGACCC CGCACCGAGC GTGGTGTCAA GACCACCACC AGCTGGGGCC GTGGGAGCGA AGGTGCCCCA GGCTCGGGGA AGATGCGGAC CTTTTACCCT CTCGGCAAGT GATTGTTGCG CGCTCTGGTG
Clustal Co   ********** ********** ********** **** ***** ******** * ********** *********  ********** ********** ********** ** **   ** ***** ***  ***  ***** ********** ***** ****

Human        AGGGGACAGT GGTACCTCGA TTTCCCGTGT GCCAGCTTCG GGACTCAGCT GTGCTTTTGT GTCTCTTTGA TTAG
Rhesus       AGGGGACAGT GGTACCGCGA TTTCCCATGT GCCAGCTTCG GGACTCAGAT GTGCTTTTGT GTCTCTTTGA TTAG
Clustal Co   ********** ****** *** ****** *** ********** ******** * ********** ********** ****
```

ENSG00000230294：

```
Human    ATGACATCCA ACCTGGGCTC TCCTGGCCCC CAGAGAAGAG CTGCCCAGTG CATGTGCTGG CTGCACCGTG ACGTCCCCTA TGAACGAGGC CCACAGGCAG TGGCTGGCTG GGGGGATGCC CCTGCCCTGA GCTGGAGTCT CCCCAGGTGG
Rhesus   ATGACATCCA -CCTGGGCTC TCCTGGCCCC CAGAGAAGAG CAGTCCAGCG CATGTGCTGG CTGCACCACA ATGTCTCCTA TGAACAAGGC CCACAGGCAG TGGCTGGCTG GGCAGACGCC CCTGCCCTGA GCTGGAGTCT CCCCAGGTGG
Clustal  **********  ********* ********** ********** * * **** * ********** *******    * *** **** ***** **** ********** ********** **  ** *** ********** ********** **********

Human    CTGCTTCTTC CCCACCCCGA GGGTCACAGC CCAGCCCAAG CTCCAAGGGA ATGGAGCAGC TGCCTGCACC AAGCTTCCCT CCTCTGGAGA AAGCACCTCC TCTTCTCCCT CACGTGCTCC CTGATGACCG CCAGGCTCCT TGACATGGTT
Rhesus   CTGCTTCTTT CCCACCCGGA GGGTCACAGC CCAGCCCAAG CTCCAAGGGA ATGGAGCAGC TGCCTGCACC AAGCTTCCCT CCTCTGGAGA AGGCGCCTCC TCTTCTCCCT CACGTGCTCC CTGATGGCCA CCATCCTCCT TGACATAGTT
Clustal  *********  ******* ** ********** ********** ********** ********** ********** ********** ********** * ** ***** ********** ********** ****** **  ***  ***** ****** ***

Human    CCCAGGACCA GCCTCTCCCA GGGTCC-CTA CTCCCAGTAC CCCAGTCCTC ACCATTTGTG A
Rhesus   CCCAGGACCA GCCTCTCCCG GGGTCAACTA CTCCCAGTAC CCCAGTCATC ACCATCTGT- -
Clustal  ********** *********  *****  *** ********** ******* ** ***** ***
```

ENSG00000233290：

```
Human    ATGAGAAATT CCGAAGCACA CATTAGAAGC AATGACGGTT ATCAGCCTAG CAGAGGCCGA TGGGCTTCCT CGGATCCTGC CATGGCACCA AAGGAATGCG AGAAAATGAT TGGCTGGGGA GCTCTTCCAC ATTGTCAGGA ACGCTGGAAA
Rhesus   ATGAGAAATT CCGAAGCACA CATTAGAAGC AATGATGGTT ATCAGCCTAG CAGAGGCTGA TGGGCTTCCT CGGATCCCGC CATGGCACCA AAGGAATGCC AGAAAATGAT TGGCTGGGGA GCTCTTCCAC ATTGTCAGGA GCACTGGAAA
Clustal  ********** ********** ********** ***** **** ********** ******* ** ********** ******* ** ********** *********  ********** ********** ********** **********  * *******

Human    AA-GGCTGAA AGAGAATGA
Rhesus   AAAGGCTGCA AGAGAATGA
Clustal  ** ***** * *********
```

ENSG00000235766：

```
Human    ATGCTCTCGT GCTGCTTGGA GCCAGCAGCG TGGGACTGTG GGGCCGAGGG CAGGGATGGG AGAGAAGAGA TGGTTCTGGG CTGGAAGCGA GACAGGGGGA CCACTCCCCG CACCCTCCCC GCCAGCCCCA GTGCGGGGAC GCCTCTCTGG
Rhesus   GTGCTCTCGT GCTGCTTGGA GCCAGCGGTG TGGGGCTGTG GGACCGAGGG CAGGGATGGG AGAGAAGAGA TGGTTCTGGG CTGGAAG-GA GACAGAGGAA CCGCCTCCTG CACCCTCCCC ACCAGCCCCT GCGCTGGGAC GCTTCTCTGC
Clustal  ********* ********** ****** * * **** ***** ** ******* ********** ********** ********** ******* ** ***** ** * ** *  ** * **********  ********  * ** ***** ** ******

Human    GGTGCAGGGC ACGTGCTTGG GGACGCTGGC GAGAGCCCCT TACCTTCACA TCCGTGTCCG AATCGCTGGA GCTGCTGCTG GAGTCGGAAG AGCTGTGGTG TCCTTGCTGG ATGGAGGTGC GGCAGTGAGG CGGCGCCCCT TACCCAGCCC
Rhesus   GGTGCAGGGC ACGGGCCTGG GGACGCTGGC GAGAACCCCT TACCTTCACA TCCATGTCCG AGTCGCTGGA GCTGCTGCTG GAGTCGGAAG AGCTGTGGTG TCCTTGCTGG ATGGAGGTGC GGCAGTGAGG CGGTGCCCCT TACCCAGCCC
Clustal  ********** *** ** *** ********** **** ***** ********** *** ****** * ******** ********** ********** ********** ********** ********** ********** *** ****** **********

Human    CCTGAAGTTG GAGGCCTAAG GCAGGACCCT GGGGTCAGGG GCAACCCCAG CCTTCCCGCC CCTCCGCAGC CGGTGATGAG GCGACTTACC TTTGGACCCG GACCTGCCCC TGCCTCCGAC CGGCCCTGA
Rhesus   CC-GAAGTTG GAAGCCCAAG GCAGGACCCT GGGGTCAGGG GCAACCCCAC CCCTCCCGCC CGTCCGCAGC CGGTGATGAG GAGACTTACC TCTGGACCCG GACCTGCCCC CGCCTCCGAC CGGCCCTGA
Clustal  ** ******* ** *** *** ********** ********** *********  ** ******* * ******** ********** * ******** * ******** **********  ********* *********
```

ENSG00000236081:

```
Human    ATGGGCTCC GCTCCTTCTC ACTCCCAGTC CTCTGGTGCA TGCCCCCTTC CTGGCTGAAG AACCTGCACC AGCCGCCCCT CCGCCTGGGA AGCTCC---- --CTCCTGTC ATTCACTCCG AGACGCAGCA GCGTTGCCCC AAGAGCCCTC
Rhesus   ACCCGCTCC GTGCCTTCTC GCTCCCTGTC C-CCGATGCA CACCCCCTTC CTGGCTGAAG ACCCTGCACC AGCCACCCCT CCACCTGGGA AGCTCCGGCT CCCTCCTGTT GTTCAGTCCA AGATGCAGCA GCATCACCTC AAGAACCCTC
Clustal  *   ****** *  *******  ***** *** * * * ****   ********* ********** * ******** **** ***** ** ******* ******       *******   **** ***  *** ****** ** *   ** * **** *****

Human    CCTGCTCTGC ACCCCGAATT CACTCTCAGC CCCCACCTAG TTTAA
Rhesus   CCTGCTCTGC ACCCCGAATT CACTCTCAGC CCCCACCTGG CTTAA
Clustal  ********** ********** ********** ******** *  ****
```

ENSG00000236314：

```
Human    ATGGGGGCTC AGTCGGGGCC CCTTCCACAG ACAGAGCCGT GGCCCCTCAA GGTGGACAGT GAGGCCGCTG CTCCTCCAGC TGCCTCTGTC TCATCACAGG CCAGAAGTGG AAAGGGTTTC AAATCCAAGC CCCCGGGTTC CTGTGTGCGT
Rhesus   ATGGGGGCTC AGTCGGGGCC CCTTCCACAG ACACAGCCCT GGCCCCTCAA GGTGGATGGC GAGGCCGCTG CTCCTCCAGC TGCCTCTGTC TCATCGCAGG CCAGAAGTGG AAAGGGTTTC AAACCCAAGC CT--GTGTTC CCGTCTGTGT
Clustal  ********** ********** ********** *** **** * ********** ******  *  ********** ********** ********** ***** **** ********** ********** *** ****** *   ***** * ** ** **

Human    TCTACGCAGC AAGGCTTCAA AGTTTCAAAC TGGTTTGGAA CTCAACCAGG TCCTCCAAAG GAAACCAAGG CAGGCACAGG ATGGGGGCAG GCTGGCTTGG CAGGCAGCTG TGCGGATCCA CCCAGAGTGC CTTGGGAGGC AGTGGGGTTG
Rhesus   TCTATGCAGC AAGGCTTGAA AGTTTCAAGC TGGTTTGGAA CTCAACCAGG TCCTCCAAAG GAAACCAAGG CAGGCACAGG ATGGGTGCAG GCTGGCTTGG CAGGCCAATG TGCCGATCCA CCCAGAGTGC CCTGGGAGGC AGTGGGGTTG
Clustal  **** ***** ******* ** ******** * ********** ********** ********** ********** ********** ***** **** ********** *****    ** *** ****** ********** * ******** **********

Human    GCTGTGGCTG CTGTGAGCTG CAGCCAGGCC TCTCCCGCGT CCCCAGGGTC TGGAAGGAGG GCCTCTGCAC ATT-TTATAA GCCACAGACT GCGAAGGCCT GGAGCCCCAC CTTCCACCCA T-AACAAAGA AAGGGATAGT TTCAGAACTG
Rhesus   GCTCTGGCTC CTGTGAACTG CAGCCAGGCC TCTCCCG-GT GCCCAGGGTC CGGAAGGAGG GGCT-TGTAC AATGTTATCA CCCGGGGACT GCGAAGGCCT GGAGCCCTAC CTTCCACCCA TTAACAGAGA AAGGGATAGT TTCAGAACTG
Clustal  *** *****  ****** *** ********** ******* **  *********  ********* * ** ** ** * * **** *  **    **** ********** ******* ** ********** * **** *** ********** **********

Human    CTTTTATTTG GATAACTGAC TGA
Rhesus   CTTTTATTTG GATAACTGAC TGA
Clustal  ********** ********** ***
```

ENSG00000240057:

```
Human    ATGCGAGGCG GGGGACTTA CGAAAGGCGG AGCTACGCGA GAGCACGTCC AGCGACCGGA -TCTGGAACA GTTCCTCGTT CTTTCTCTGG CTCTGGATCT GCGTCCCTGA CAGTCTTAAA GAGGACAGAC CTTTCATTCA TGAGGATGAC
Rhesus   TTGCGAGGCG GGTAGATCTA GGAAAGGCGG AGCTACGCGA GAGCACGTCT AGCGACCGGA GTCTGGAACA GTTCCTCCTT CTTTCTCTGG CTCTGGATCT GCGTCCCTGA CAGTCTTAAA GAGGACAGAA CTTTCATTTA TGAGGGTGAC
Clustal  ********* **  **  **  ********* ********** *********  **********  ********* ******* ** ********** ********** ********** ********** *********  ******** * ***** ****

Human    CCTCAACTTG GCCCAGTCCA TGTTTCCTCA GACTCAAGCC TTGCATTCCT GGCAGAAAGA CTGAAGAAAT GA
Rhesus   CCTCAACTTG GCTCAGTCAA TGTTTCCTCA GACTCAAGCC TTGCATTCCT GGCAGAAAGA CTGAAGAAAT GA
Clustal  ********** ** ***** * ********** ********** ********** ********** ********** **
```

ENSG00000243926：

```
Human    ATGGAGGGTC ACTTTGTTCC GAGAAGACCC AGACACCCGA GGGAGGATGT GAAACGGAAC GACCCGTTTC GAGTAATAAC AGGATGGAGA TGGAGTTTCA TGACAAAAAT GAAGGTGAAT ATGATGTGGA AAATACAAAG ATGGAAGCAA
Rhesus   ATGGTGGGTC ACTTTGTTCC GAGGAGACCC AGGCACCCGA GGGAGGATGT GAAACGGAAC GACCCGTTTC GAATAATAAC AGGATGGAGA TGGAGTTTCA TGACAAAAAT GAAGGTGAAT ATGATGTGGA AAATATAAAG ATGGAAGCAA
Clustal  **** ***** ********** *** ****** ** ******* ********** ********** ********** ** ******* ********** ********** ********** ********** ********** ***** **** **********

Human    CAAGCTAAGG ATGCTGGGGC AGAAAGAT-- --GGAACCTG GGTTACTCAT GA
Rhesus   CAAACTAAGG ATGCTGGGGC AGAAAGATAG AAGGAAACTG GGTCACTCAT AA
Clustal  *** ****** ********** ********      **** *** *** ******  *
```

ENSG00000244291：

```
Human    ATGCTCGCCA GCCCCGCCCG GCCCACATTG CGCATGCTTG CCAACCACGC CCTGTCCACA CCACACTGCG CATGCTCGCC GGCCCCCGCC CCGCGCACGG CGTCAGCGTC TAGACGCAGG TGCGTTCCCG TCGAGGCTCG CGCAGCGGGC
Rhesus   ATGCTCGCCA GCCCCGCCCG GCCCACACTG CGCATGCTTG CCGGCCACGC CCGGTCCACA CCACATTGCG CATGCTCACC GGCCTCAGCC CCGCGCCCGC CGTCAGCGTC GAGACGCAGG TGCGTGCCAG TCGAGGCTCG CTTTGCGGGC
Clustal  ********** ********** ******* ** ********** **   ****** ** ******* ***** **** ******* ** **** * *** ****** **   **********   ********* ***** ** * ********** *   ******

Human    GTTTTTGGGG ATCGCCTGGC GGGGGTGTTC GGGAGCCGCG GGCTGAAGCA CGGGGGCGTG CAGGCGCCCA GGCCTCGTGT AGTGA----- -------GGG CCGAGCCCCG CGCCGGCTTC GCTGTAGTCC GCAGCCCCCG GCGGCTTTGT
Rhesus   GTGTTTGGGG ATTGCCTGGC AGGG-TGTTT GGGCGCCGCG GGCTGACGCG CAGGGGCGTG CAGCCGCCCA GGCCTCATGC AGTGACTCGT GTCGTGAGGG CCGAGTCCCG CGCCGGGTTC GCTGTAGTCC GCAGCCGCTG GCGGCTTTGT
Clustal  ** ******* ** *******   *** ****  *** ****** ****** **   * ******** *** ****** ****** **  *****            *** ***** **** ****** *** ********** ****** * * **********

Human    GGGCGCAGCC ACGCTCCTCA GCCGCCCGCC CACCTGGGCC TGGGCCCCGG TTGTTTCCCT GCGGTGGCTG TGGTCGTTCC CGTTCCAGGG TCTCGAGCGC ACCGCCCCTT CGCTGCGCTG TTAGTCGAGG GCAGTTTTCT GGGGGATCCC
Rhesus   GGGTGCAGCC ACGCTCCTCA GCCGCCCGCC CACCTGGGCC TGGGACCCGG TCGTTTCCCT GCG-TGTCTG TGGTCGTTCC CGTTCCAGGG TCTCGAGCGC ACCGCCCCTT CGCTGCGCTT TTAGTCGAGG GCAGTTTTCT GGGGCATTCC
Clustal  *** ****** ********** ********** ********** **** ***** * ******** *** ** *** ********** ********** ********** ********** *********  ********** ********** **** ** **

Human    CCGATCCCCC CGCGCCGCTC AGGCGTCTTG GCTCGCGGAA GCGCAGGCGC CGACTGCCTG GCGTCGTCCG TGACTCCTGG CCCTTCTCTG TGGATCCCTT TGCTTCTCGT TGCGGGTTGT GTTTCCTGCT TCGTGGGCCT GGCCGTTTGC
Rhesus   CCGGTCCCCC TGTGCCTCTC GGGCGTCCTG GCCCGCGGAA GCGTGGGCGC CGACTGCCTG GCGTCGTCCG TGACTCCTGG CCCCTCTCTG TGGATCCCGT TGCTTCTCGC TGCGGGTTGT GTTTCCTGTT TCGTGGGCCT GGCCGTTTGC
Clustal  *** ******  * *** ***  ****** ** ** ******* ***   ***** ********** ********** ********** *** ****** ******** * *********  ********** ******** * ********** **********

Human    GTTTGGATGC AGGCCCGGGT GAGCCCTGCG TGGCCCGCTG GCCTTTTCCT GCTCCCTAGG TGA
Rhesus   GTT-GGATGC GGGCCCCGGT GATCCCTGCG TGGCCCGCTG GCCTTTTCCT GCTCCCGAGG TGA
Clustal  *** ******  ***** *** ** ******* ********** ********** ****** *** ***
```

ENSG00000247796:

```
Human    ATGGGCCGTG GTGGTGAGAG GAAGCAATCA GTATGGAAAT GGAGAAATGG CTTACCATGC ATGTCCTTCT CCCCAGGAGG GAGGTCGTGG TGCAGGCGAG CAGACGCCAG ACTCC--TGG ACACTTGCCG CCTGCGTTAT GGAGGCTTAG
Rhesus   ATGGGTCTTG GCGGTGAGAG CGAGCAATCA GTATGAAAAT GGAGAAAGGG TTGACCATGT ATGTC---CT CCCCAGGAAG GAGGTCGTGG TGTAGGCGAG CAGAGGCCAG ACTCCCGTGG ACACTTGCCG CCTGCGTTAT GGAGGCTTAG
Clustal  ***** * ** * ********    ******** ***** **** ******* **  * ******  *****   ** ******** * ********** ** ******* **** ***** *****   *** ********** ********** **********

Human    CTAATAACCT TGCAGGGGTC TTCAGGCTTT TCCACTGGCT CACCTTTAGG CTTCTGACAC TTTTACCTAC GATTGAAAAA AGACGAGATT CCATGAGACT AGAAATGTAG
Rhesuse  CTAATAACCT TGCAGGGGCC TTCATGCTTT TCCACTCGCT CACCTGTAGG CTTCCGACAC T--TACCTAC GATT-AAAAA AGATGAGATT CCATGAGACT AGAAATGTAG
Clustal  ********** ******** * **** ***** ****** *** ***** **** **** ***** *  ******* **** ***** *** ****** ********** **********
```

ENSG00000258376：

```
Human    ATGGGGAAAT CGGCCAGGAG GGGCTGGGGA AGCTGGTGCC TTCGCCCGCG CCCCGTGAGC TTTACCTCCG TGCGACAAGA GCGGTGGCTC CGGGCGGGGC CCACGCAGCT GGAGCCTCCA TCTCTCCTGC GGGACCAGAG CACCGGGAAG
Rhesus   GTGGGGACGT CGGCCGGGAG GGGTTGGGGT AGCTGGTGCC CTCGCCCGCA CCCCGTGGGC TTTACCTCAG TGCGACAAGA GCGGTGGCTC CGGGCGGGGC CCACACAGCT GGCGCCTCCA TCTCTCCTGC GGGACCAGAG CACCGGGAGG
Clustal   *****   * ***** **** *** *****   **********  ********   ******* ** ******** * ********** ********** ********** **** ***** ** ******* ********** ********** ******** *

Human    ACCTGA-
Rhesus   ACCCGGA
Clustal  *** *
```

ENSG00000260456：

```
Human    ATGCGTGCGA GCCGGTCCCC ACCGTCCCCG CGGCGTTGTC ACCATCATCA TGAGGCCACT GGAGCAGCCT CAGGCGCTGC TGCCGGGGGG CCGGGCGCGG GGTGCGTCGG GCTCTGCAGG TTGGCACTCA CACCCAGCGC GCAGGATGGC
Rhesus   ACGCGTGCGA GCCGGTCCCC ACTGCCCCCG CGGCGTTTTC ACCATCATCA TGAGGCCACT GGAGCAGCCT CGGGCGCTGC TGACGGGGAT CCGGGCGCGG GGTGCGTCGG GCTCTGCAGG TTGGCACTCA CACCCGGCGC GCAGGATGGC
Clustal  * ******** ********** ** * ***** ******* ** ********** ********** ********** * ******** ** *****   ********** ********** ********** ********** ***** **** **********

Human    AGGAATAGCA CATTTCAAAC CTACAAGAAA GAAGTGTGCC TCCCCCGTCA TTCGATGCAC CCTGGCCCCT GGGCCATCTG CTGTGAATGC CAGACCAGAT TCGGGGGCCG CCTGCCTGTG TCCAGGGTGG AAGCAGCACT GCCTTACTGG
Rhesus   AGGAATAGCC CATTTCAAAC CTACAAGAAA GAAGTGTGCC TCCCAGGTCA TTCGATGCAC CCTGGCCCCT GGGCCGTCTG CTGTGAGTGC CAGACCAGAT TCGGGGGCCG CCTACCTGTG CCCAGGGTGG AAGCAGCACT GCCTTACTGG
Clustal  *********  ********** ********** ********** ****  **** ********** ********** ***** **** ****** *** ********** ********** *** ******  ********* ********** **********

Human    GTCCCTCTGT CCCTGAGACC CCGAAAGCAG CACCCCTGCT GGATGCATGC TGCTGGCACA ACTGCTGGCG GATCTGCGGT GATGAGTGCC TGTTGTCCAA GTTCCAGCAG CTCCAGGCCC CCTACCAGGA CCAGCTACCG GCTCCTGCAG
Rhesus   GTCCCTCTGT CCCTGAGACC CCGAAAGCAG CACCCCCGCT GGATGCATGC TCCTGGCACA ACTGCTGGCG GATCTGCGGT GACGAGCGCC TGCTGCCCAA GTGCCAGCAG CTCCAGGCCC CCGACCAGGA CGAGCTACCG GCTCGCCCAG
Clustal  ********** ********** ********** ****** *** ********** * ******** ********** ********** ** *** *** ** ** **** ** ******* ********** ** ******* * ******** ****   ***

Human    CGCGTCTGCT GCCCCTCGGC CTCCTGA
Rhesus   TGTGTCTGCT GCGCCTTGCC CTCCTGA
Clustal   * ******* ** *** * * *******
```

ENSG00000261644：

```
Human    ATGTCCTTTA TGACCTTACA GCTCACCTGG AACCCTCCAC CTAGGGCCCG AGGCAGCACA TCCTCTCGTG CCCGGGCACT TCCAGGCATT GCCCTGAGTA CTGGGCACTT CACACAGTGG GCTGGACCAC TCACCGGAGT GAATAAGGAG
Rhesus   ATGTCCTTTA TGACCTTACA GTTCACCTGG AACCCTCCAC CCAGGGCCTG AGGCAGCGCA TTCTCTTGTG CCCAGGCACT GCCAGGCATT GCCCTGAGTA CTGGGCGCCT CACACAGTGA GCTGGACCAC TCACCCGAGT GAAGAAGGAG
Clustal  ********** ********** * ******** ********** * ****** * ******* ** * **** *** *** ******  ********* ********** ****** * * *********  ********** ***** **** *** ******

Human    GCTTGA
Rhesus   GCTTGA
Clustal  ******
```

ENSG00000180838:

```
Human    ATGGGGAGGG ACAGATGGGT GGAGGAGTGG AAGATGGAGG ATGGGGAGGG ACAGATGAGT GGAGGAGTGG CAGATGGAG- ---------- --GAGGATGG CTGGAG---- ---------- ------GGAC AGAT------
Rhesus   ATGGGGAGGG ACAGATGGGT GGAGGAGTGG CAGATGGAGG ATGGGGAGGG ACAGATGGGT GGAGGAGTGG CAGATGGAGA GATGGGGACG AAGACGGTGG ATGAGGATCG GGGCGAGGAC ACCGGTGGGC AGATAGAGGG
Clustal  ********** ********** **********  ********* ********** ******* ** ********** *********              ** * ***  **  *                            ** * ****

Human    ----GCTG-- ---------- GGTGATTGTT GGGGGTGCAG GTGCGGGACG GGTCGGGGAC AGGTG-GAAG GAAAACAGCA CACAGATGCG GA--CAGA-T GGCCAGAAAG ATT-AATAGT TCAGTGGCAG AGACCTAGGC C--TGGCGGC
Rhesus   GATGGCTGAG GGACAATTGT GGTGATTGTT CGGCG-GCAG GTTGGGGACG GGTGAGGGAT GGGTGTGAAG GAAAACAGCA CATAGGTGTG GGGCCAGAAT GGCCAGAAGG AATGAATAGT TCAGTGGCAG AGACCTAAGG CACTGGCATC
Clustal      ****              ********** ** * **** **  ****** ***  ****   **** **** ********** ** ** ** * *   **** * ******** * * * ****** ********** ******* *  *  ****  *

Human    TGTCGTCCCC CCACTCACGG ATGCGCGCGG TCCAGCACTG CTCCCCCAGC TGGGTGCAGT TCTCCACCTG CAGGCAGTC- CTCGTTGCTC ACCTGGG--C TTTGCAGGAG TAGCACAGCA G---GGCAGT GCCTGTGGGG TGGAGTGGGA
Rhesus   TTTCGTCTCC CCA-TCACGG ATGCGC-TGG TCCCGCTCTC CTCCGGC--C TGCGTGCAGT TCTCCACATT CAGGCAGTCT CTCGTTGCTC ACCTGGGGCC TTTGCAGGGG TAGCACAGCA GCAGGGCAGT GC-TGTGGGG TGGAGTGGGA
Clustal  * ***** ** *** ****** ******  ** *** ** **  ****  *  * ** ******* ******* *  *********  ********** *******  * ******** * ********** *   ****** ** ******* **********

Human    GGGGCCTCAG AGGCTGCCCC ATAG
Rhesus   AGGGGCTCAG AGGCTGCCAC -TAG
Clustal   *** ***** ******** *  ***
```

ENSG00000253929:

```
Human    ATGCAGTGTC CAAGGTGGAG AAAGGTCAGA GTGGATCCAG AGGAGCCAAG AGAAGACGTC CAGCATGGTG ACCTGGGCTC AAGTCAAGGT CCTTATTTCT CTGCTGAACC ATCACATTCC TTATACAGAG TGGGACAGCA TTGGCTGAGT
Rhesus   ATGCAGTGTC CAAGGTGGAG AGAGGTCAGA GTGGATCCAG AGGAGCCAAG AGAAGATGTC CGGCACAGTG ACCTAGGCTC AAGTCAAGGT CCTTATTTCT CT-CTGAACC ATCACAGTCC TTGTGCAGAG TGAGGCAGCA CTGACTGAGT
Clustal  ********** ********** * ******** ********** ********** ****** *** * ***  *** **** ***** ********** ********** ** ******* ****** *** ** * ***** ** * *****  ** ******

Human    TCTACTAGCA AATGCCGGAG ATGTGGTTCC TGTTGGCCTT TTAACTGA
Rhesus   TCTACTAGCA AATGCTGGAG ATGTGGTTCC TGTTGGCCTT TTAACTGA
Clustal  ********** ***** **** ********** ********** ********
```

ENSG00000257135:

```
Human    ATGTGCTGGC CACGTGGGCC CCGCCGGGCC GACTCCGGAC CCTGCTGCCG CCCATTCCTC CGGTGC-AAG GGACCTGGAA GTGATCCCGG TGCCGCTCTG CTCCCACCAG CTAGGGAGCA GAGGCGCGGG AGGGAAGGGG CGTCCCGGGG
Rhesus   GTGTGCTGGC CACGTGGGCC CCGCCGGGCC GACTC--GAC CGTGCTGTCG CCCGCCGCTC CAGTGCTAAG GGACCTGGAA GAGATCCCGG TGCAGCTCCG CTCCCACCAG CGAGGGAGCA GAGGCGCGGG AGGGAAGGGG CGTCCCGGGG
Clustal   ********* ********** ********** *****  *** * ***** ** ***    *** * **** *** ********** * ******** *** **** * ********** * ******** ********** ********** **********

Human    GTCGCCAGCA CGCTGCGGGC AGGGCTGGCA GCCGCCTCCA CGCCCCCGCG CTGGAAGCCT CGTCTTTGTA ACATCGAGGT ATCCTGTAAC TAGATGGGTC CTGGCCTGCG GAGGTAGTTT CCCCTGTGAA GCCAGGGACT CAGCCAGGAG
Rhesus   GTCGCCAGCA CGCTGGGGGC ACGGCTGGCA GCCGCCCCCA CGC------- -TGGAAGCCT CGTCTTTGTA ACATCC-GGT GTCCTGTAAC TAGATGGGTC CTGTCCTGCG GAGGTGGTTT CTCCTGTGAA GCCAGAGATT CACCCAGGAG
Clustal  ********** ***** **** * ******** ****** *** ***         ********* ********** *****  ***  ********* ********** *** ****** ***** **** * ******** ***** ** * ** *******

Human    CGTGAGGGGA CCCGGATCCT GA
Rhesus   CGTGAGGGGA GCCGGGTCCT GA
Clustal  **********  **** **** **
```
