# Supplementary material for: De novo genes with an lncRNA origin encode unique human brain developmental functionality
Source: Nat Ecol Evol. 2023 Jan 2;7(2):264–78. doi: 10.1038/s41559-022-01925-6 (PMC9911349; doi:10.1038/s41559-022-01925-6)
Supplement: Supplementary file 2 — Reporting Summary [file 41559_2022_1925_MOESM2_ESM.pdf]

Corresponding author(s): Chuan-Yun Li

Last updated by author(s): Sep 19, 2022

Reporting Summary

Nature Portfolio wishes to improve the reproducibility of the work that we publish. This form provides structure for consistency and transparency in reporting. For further information on Nature Portfolio policies, see our Editorial Policies and the Editorial Policy Checklist.

Statistics

For all statistical analyses, confirm that the following items are present in the figure legend, table legend, main text, or Methods section.

n/a	Confirmed	
☐	☒	The exact sample size (*n*) for each experimental group/condition, given as a discrete number and unit of measurement
☐	☒	A statement on whether measurements were taken from distinct samples or whether the same sample was measured repeatedly
☐	☒	The statistical test(s) used AND whether they are one- or two-sided<br>*Only common tests should be described solely by name; describe more complex techniques in the Methods section.*
☐	☒	A description of all covariates tested
☐	☒	A description of any assumptions or corrections, such as tests of normality and adjustment for multiple comparisons
☐	☒	A full description of the statistical parameters including central tendency (e.g. means) or other basic estimates (e.g. regression coefficient) AND variation (e.g. standard deviation) or associated estimates of uncertainty (e.g. confidence intervals)
☐	☒	For null hypothesis testing, the test statistic (e.g. F, t, r) with confidence intervals, effect sizes, degrees of freedom and P value noted<br>*Give P values as exact values whenever suitable.*
☐	☒	For Bayesian analysis, information on the choice of priors and Markov chain Monte Carlo settings
☐	☒	For hierarchical and complex designs, identification of the appropriate level for tests and full reporting of outcomes
☐	☒	Estimates of effect sizes (e.g. Cohen's *d*, Pearson's *r*), indicating how they were calculated

Our web collection on statistics for biologists contains articles on many of the points above.

Software and code

Policy information about availability of computer code

Data collection	No software was used for data collection in this study.
Data analysis	TopHat2 (version 2.1.1), Ribotaper (version 1.3.1a), HISAT2 (version 2.0.5), Stringtie (version 1.3.4d), Tomtom (version 5.0.5). The in-house codes in this study can be found at GitHub via URL: https://github.com/ZhangJiePKU/DenovoProject

For manuscripts utilizing custom algorithms or software that are central to the research but not yet described in published literature, software must be made available to editors and reviewers. We strongly encourage code deposition in a community repository (e.g. GitHub). See the Nature Portfolio guidelines for submitting code & software for further information.

Data

Policy information about availability of data

All manuscripts must include a data availability statement. This statement should provide the following information, where applicable:

- Accession codes, unique identifiers, or web links for publicly available datasets
- A description of any restrictions on data availability
- For clinical datasets or third party data, please ensure that the statement adheres to our policy

High-throughput sequencing data from this study have been submitted to the NCBI Sequence Read Archive (SRA) (https://www.ncbi.nlm.nih.gov/sra/) under accession number PRJNA750575.

Field-specific reporting

Please select the one below that is the best fit for your research. If you are not sure, read the appropriate sections before making your selection.

☒ Life sciences ☐ Behavioural & social sciences ☐ Ecological, evolutionary & environmental sciences

For a reference copy of the document with all sections, see nature.com/documents/nr-reporting-summary-flat.pdf

Life sciences study design

All studies must disclose on these points even when the disclosure is negative.

Sample size	We applied population genetics analyses of a population of 652 humans and 572 macaques in this study, following previous experience in population genetics studies with sufficient statistical power.
Data exclusions	No data were excluded in this study.
Replication	At least three independent experiments were performed to investigate the function of the de novo genes in organoids. All attempts at replication were successful.
Randomization	This is not relevant to this study. As this is a comparative study between wild type and transgenic hESCs.
Blinding	The investigators were blinded to the wild type and transgenic hESCs group in the downstream computational analyses.

Reporting for specific materials, systems and methods

We require information from authors about some types of materials, experimental systems and methods used in many studies. Here, indicate whether each material, system or method listed is relevant to your study. If you are not sure if a list item applies to your research, read the appropriate section before selecting a response.

Materials & experimental systems

n/a	Involved in the study
☐	☒ Antibodies
☐	☒ Eukaryotic cell lines
☒	☐ Palaeontology and archaeology
☐	☒ Animals and other organisms
☒	☐ Human research participants
☒	☐ Clinical data
☒	☐ Dual use research of concern

Methods

n/a	Involved in the study
☒	☐ ChIP-seq
☒	☐ Flow cytometry
☒	☐ MRI-based neuroimaging

Antibodies

Antibodies used	lamin-B (Bioworld, AP6001), SOX2 (R&D, AF2018), PAX6 (Thermo Fisher, 42-6600), CTIP2 (Abcam, ab18465), NEUN (Abcam; ab177484)
Validation	lamin-B: https://www.bioworlde.com/Primary-Antibodies/25204.html, SOX2: https://www.rndsystems.com/cn/search?keywords=%20AF2018, PAX6: https://www.thermofisher.cn/cn/zh/antibody/product/PAX6-Antibody-Polyclonal/42-6600, CTIP2: https://www.abcam.cn/ctip2-antibody-25b6-ab18465.html, NEUN: https://www.abcam.cn/neun-antibody-1b7-neuronal-marker-ab104224.html

Eukaryotic cell lines

Policy information about cell lines

Cell line source(s)	HEK293T, LLCMK2
Authentication	The genome and transcriptome of these two cell lines were acquired to confirm the identity.
Mycoplasma contamination	All cell lines were tested negative for mycoplasma contamination.
Commonly misidentified lines (See ICLAC register)	No commonly misidentified lines used in this study.

Animals and other organisms

Policy information about studies involving animals; ARRIVE guidelines recommended for reporting animal research

Laboratory animals	Macaca mulatta, rhesus macaque, male, 7.3 years old.
Wild animals	The study did not involve wild animals.
Field-collected samples	This study did not involve samples collected from the field.
Ethics oversight	All samples used in this study were obtained and manipulated from the internationally-accredited animal facility (Association for Assessment and Accreditation of Laboratory Animal Care, AAALAC) at the Institute of Molecular Medicine, Peking University. The present study was approved by the Animal Care and Use Committee of Peking University (IMM-LiCY-1, IMM-HeAiB-1).

Note that full information on the approval of the study protocol must also be provided in the manuscript.
