# Supplementary figures and images for: De novo genes with an lncRNA origin encode unique human brain developmental functionality
Source: Nat Ecol Evol. 2023 Jan 2;7(2):264–78. doi: 10.1038/s41559-022-01925-6 (PMC9911349; doi:10.1038/s41559-022-01925-6)

Figure 1b

Human brain-laminB

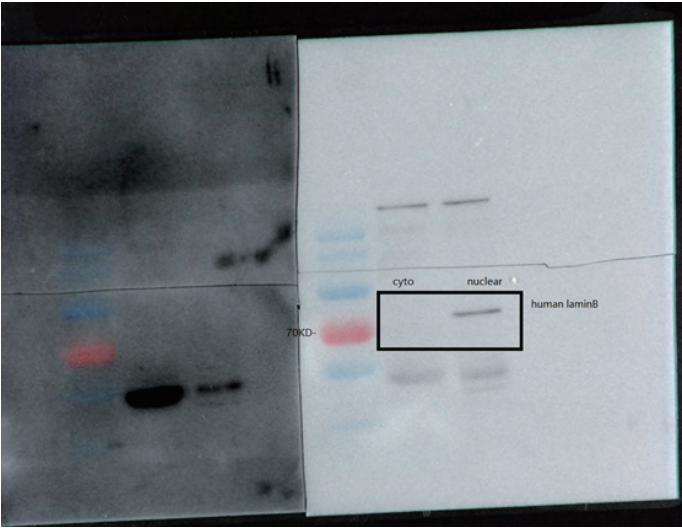

Human brain-tubulin

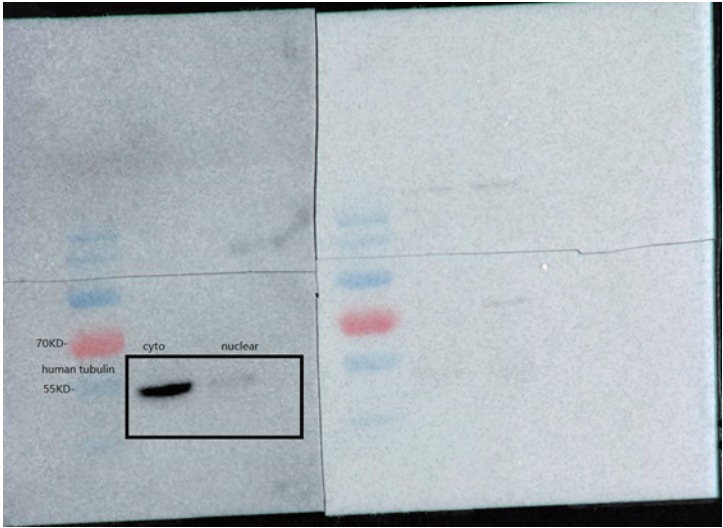

Macaque brain-laminB

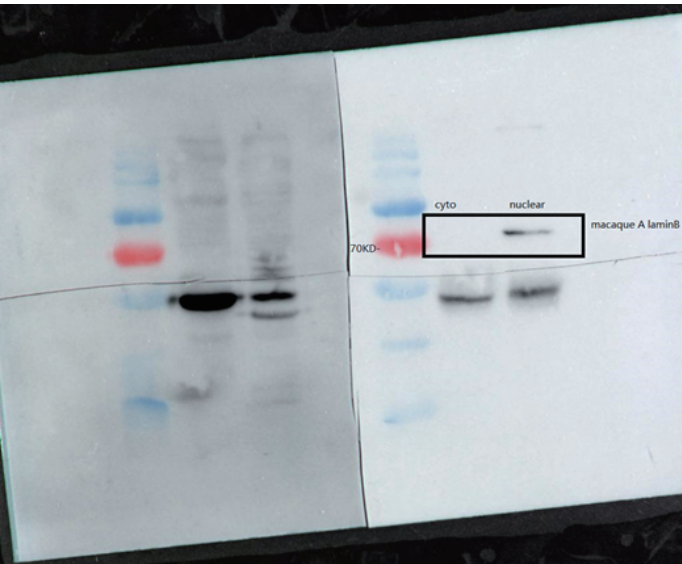

Macaque brain-tubulin

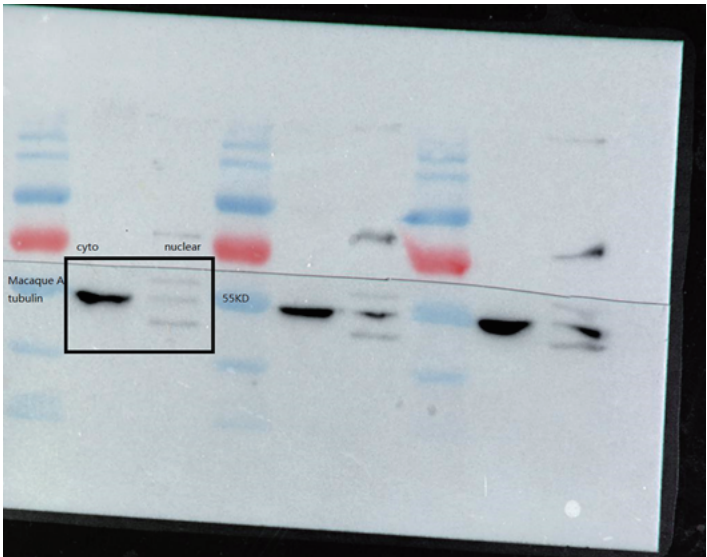

Supplement: Supplementary file 4 — Unprocessed western blots for Fig. 1. [file 41559_2022_1925_MOESM4_ESM.pdf]

Extended data Figure 2a

HEK293

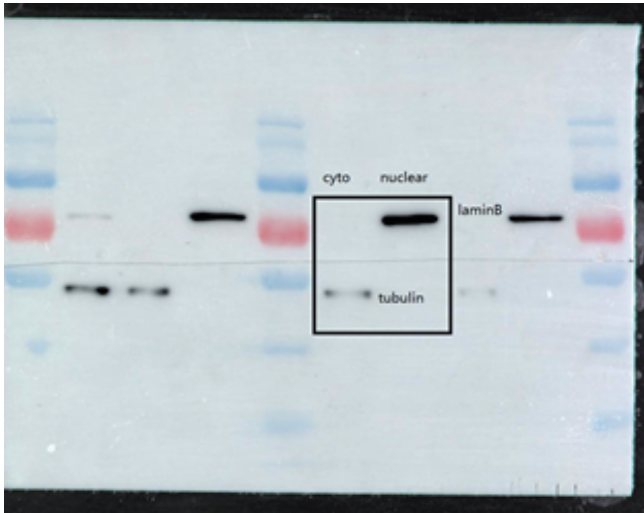

LLCMK2

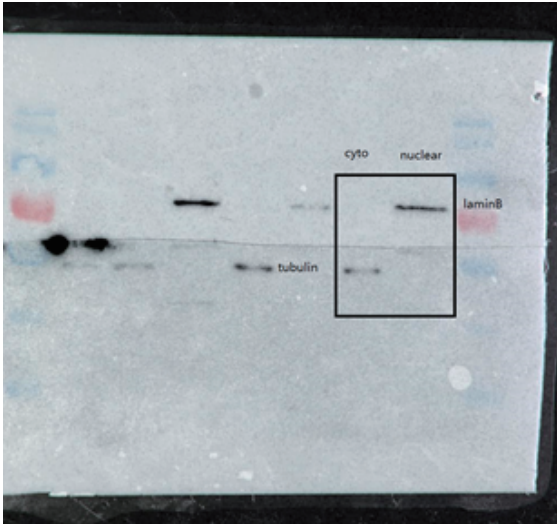

Supplement: Supplementary file 5 — Unprocessed western blots for Extended Data Fig. 2. [file 41559_2022_1925_MOESM5_ESM.pdf]

Extended data Figure 7e

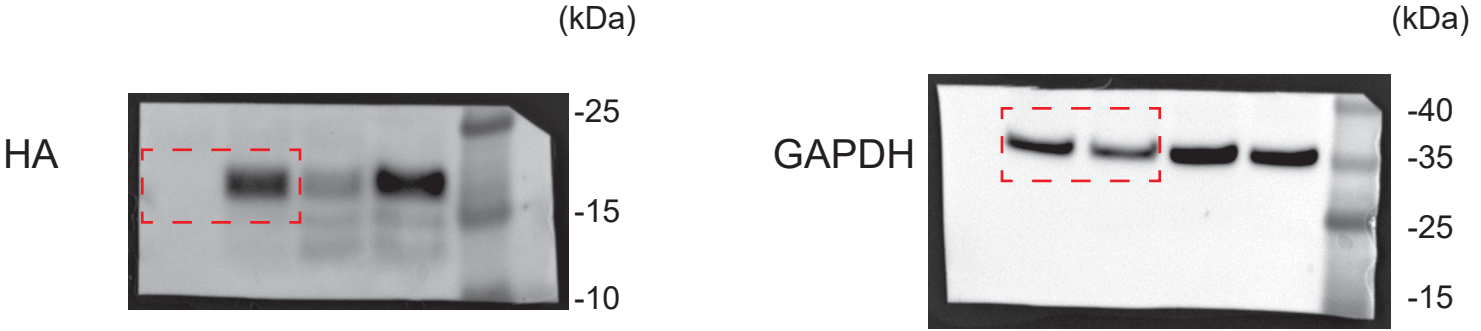

Supplement: Supplementary file 6 — Unprocessed western blots for Extended Data Fig. 7. [file 41559_2022_1925_MOESM6_ESM.pdf]
